# Interactions between SQUAMOSA and SHORT VEGETATIVE PHASE MADS-box proteins regulate meristem transitions during wheat spike development

**DOI:** 10.1093/plcell/koab243

**Published:** 2021-11-02

**Authors:** Kun Li, Juan M Debernardi, Chengxia Li, Huiqiong Lin, Chaozhong Zhang, Judy Jernstedt, Maria von Korff, Jinshun Zhong, Jorge Dubcovsky

**Affiliations:** Department of Plant Sciences, University of California, Davis, California 95616, USA; Howard Hughes Medical Institute, Chevy Chase, Maryland 20815, USA; Department of Plant Sciences, University of California, Davis, California 95616, USA; Howard Hughes Medical Institute, Chevy Chase, Maryland 20815, USA; Department of Plant Sciences, University of California, Davis, California 95616, USA; Howard Hughes Medical Institute, Chevy Chase, Maryland 20815, USA; Department of Plant Sciences, University of California, Davis, California 95616, USA; Howard Hughes Medical Institute, Chevy Chase, Maryland 20815, USA; Department of Plant Sciences, University of California, Davis, California 95616, USA; Department of Plant Sciences, University of California, Davis, California 95616, USA; Institute for Plant Genetics, Heinrich Heine University, Düsseldorf 40225, Germany; Cluster of Excellence on Plant Sciences “SMART Plants for Tomorrow’s Needs”, Heinrich Heine University, Düsseldorf 40225, Germany; Institute for Plant Genetics, Heinrich Heine University, Düsseldorf 40225, Germany; Department of Plant Sciences, University of California, Davis, California 95616, USA; Howard Hughes Medical Institute, Chevy Chase, Maryland 20815, USA

## Abstract

Inflorescence architecture is an important determinant of crop productivity. The number of spikelets produced by the wheat inflorescence meristem (IM) before its transition to a terminal spikelet (TS) influences the maximum number of grains per spike. Wheat MADS-box genes *VERNALIZATION 1* (*VRN1*) and *FRUITFULL 2* (*FUL2*) (in the *SQUAMOSA*-clade) are essential to promote the transition from IM to TS and for spikelet development. Here we show that SQUAMOSA genes contribute to spikelet identity by repressing MADS-box genes *VEGETATIVE TO REPRODUCTIVE TRANSITION 2* (*VRT2*), *SHORT VEGETATIVE PHASE 1* (*SVP1*), and *SVP3* in the *SVP* clade. Constitutive expression of *VRT2* resulted in leafy glumes and lemmas, reversion of spikelets to spikes, and downregulation of MADS-box genes involved in floret development, whereas the *vrt2* mutant reduced vegetative characteristics in spikelets of *squamosa* mutants. Interestingly, the *vrt2 svp1* mutant showed similar phenotypes to *squamosa* mutants regarding heading time, plant height, and spikelets per spike, but it exhibited unusual axillary inflorescences in the elongating stem. We propose that *SQUAMOSA–SVP* interactions are important to promote heading, formation of the TS, and stem elongation during the early reproductive phase, and that downregulation of *SVP* genes is then necessary for normal spikelet and floral development. Manipulating *SVP* and* SQUAMOSA* genes can contribute to engineering spike architectures with improved productivity.

## Teaser

Functional characterization of developmental genes reveals ways to modify the wheat spike architecture to increase the number of grains and improve productivity.

##  

**Figure:**
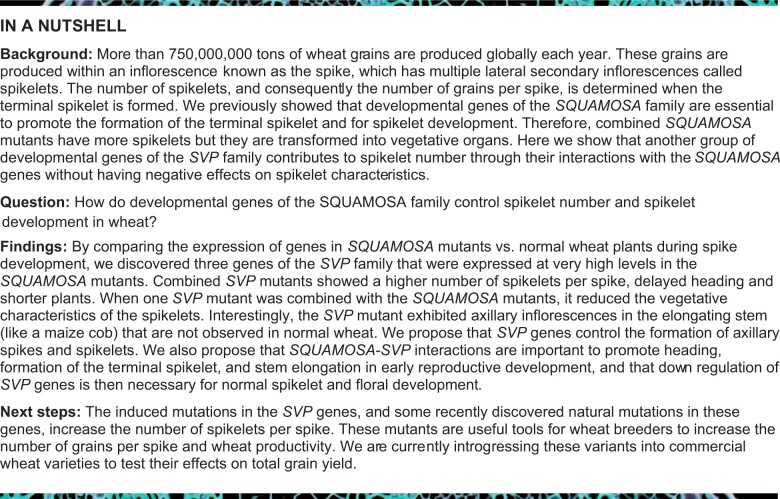


## Introduction

Each year more than 750,000,000 tons of wheat (*Triticum aestivum* L.) grains are produced around the world, providing one-fifth of the calories and protein consumed by the human population (FAOSTAT, 2017). These wheat grains are produced in an inflorescence called the spike, which is generated by the inflorescence meristem (IM). The IM first produces multiple axillary meristems, called spikelet meristems (SMs), each subtended by a suppressed leaf ridge. Then, the SMs differentiate into sessile spikelets on the spike axis (rachis) and the IM transitions into a terminal spikelet (TS), resulting in a determinate inflorescence.

The spikelet is the basic unit of the grass inflorescence (Kellogg, 2001) and, in wheat, it comprises two basal sterile bracts (glumes) and an indeterminate number of florets. Each floret has a bract called lemma with an axillary floral meristem (FM) that generates a two-keeled structure called palea, two scales called lodicules, three stamens, and a terminal ovary (Clifford, 1987). In wheat, the SM produces an indeterminate number of FMs on an axis called rachilla, with only the most basal florets surviving to set grains (Sakuma et al., 2019).

Variation in the activity and maturation rate of meristems has profound effects on inflorescence architecture and crop productivity (Park et al., 2014; Liu et al., 2021b). In wheat, the timing of the transition from the IM to TS determines the number of spikelets per spike, which, together with the number of fertile florets per spikelet, determines the maximum number of grains that a spike can produce. Because these are important components of grain yield, a better understanding of their regulatory mechanisms will be useful to engineer more productive wheat plants.

Significant progress has been made in understanding the pathways regulating grass inflorescence development, particularly in rice (*Oryza sativa* L.) and maize (*Zea mays* L.). A complex gene network involving several members of the MADS-box gene family regulates the identity shifts of different meristems in these species (Callens et al., 2018; Wu et al., 2018; Chongloi et al., 2019). During floral development, MADS-box proteins act as tetrameric complexes, and different protein combinations result in the specification of different organ identities, as has been documented in the ABCDE model of flower development in Arabidopsis (*Arabidopsis thaliana* (L.) Heynh.) (Theissen et al., 2016) and rice (Wu et al., 2018), which is defined by the five classes of homeotic genes involved, named A, B, C, D and E .

In wheat, there is currently limited knowledge of the role of these genes in spike development. We have recently shown that wheat MADS-box meristem identity genes *VRN1* and *FUL2* from the *SQUAMOSA*-clade are essential for the transition of the IM to a TS (Li et al., 2019). In the *vrn1 ful2* loss-of-function mutant, the inflorescence remains indeterminate and fails to produce TS, whereas in the single *vrn1* or *ful2* mutants the transition to TS is delayed, leading to an increased number of spikelets per spike.

In addition, *SQUAMOSA* genes are essential for spikelet identity specification. In the *vrn1 ful2* mutant, the axillary meristems in the spike develop into vegetative structures resembling tillers, some of which have residual flower organs. When the loss-of-function mutations in *FUL3* homeologs (the third member of the *SQUAMOSA*-clade) were combined in a *vrn1 ful2 ful3* mutant, the spike axillary meristems generated fully vegetative tillers and the leaf ridges were de-repressed and formed leaves (Li et al., 2019). These results demonstrated that *VRN1*, *FUL2*, and *FUL3* have redundant and essential roles in SM identity, spikelet development, and repression of the lower leaf ridge.

The wheat *SQUAMOSA* genes also regulate the initiation of reproductive development and affect both heading time and plant height. *VRN1* is a key gene affecting flowering in wheat (Yan et al., 2003). Spring wheat varieties carrying dominant *Vrn1* alleles do not have a vernalization requirement, whereas winter wheat varieties with the functional but recessive *vrn1* allele require several weeks of vernalization to acquire flowering competence (Yan et al., 2003; Fu et al., 2005; Kippes et al., 2018). The transition from a vegetative meristem (VM) to an IM is delayed in the *vrn1-*null mutant, further delayed in *vrn1 ful2* and the most greatly delayed in *vrn1 ful2 ful3*, which indicates redundant roles of these three genes in the regulation of the initiation of the reproductive phase (Li et al., 2019). Functional redundancy was also observed for plant height, with the *vrn1 ful2 ful3* mutant being shorter than any other mutant combinations (Li et al., 2019).

In this study, we aimed to identify the gene network regulated by *VRN1* and* FUL2* during the early stages of spike development in wheat. In particular, we wanted to identify the specific genes responsible for the reversion of spikelets to tillers in the *vrn1 ful2* mutant. By comparing the developing spike transcriptomes of *vrn1 ful2* (spikelets transformed into tillers) and *vrn1* mutants (normal spikes), we identified three MADS-box genes of the *SHORT* *VEGETATIVE* *PHASE* (SVP) clade that were upregulated in the *vrn1 ful2* mutant. These genes include *SVP1*, *VEGETATIVE* *TO REPRODUCTIVE TRANSITION 2* (*VRT2*, synonymous *SVP2*; Kane et al., 2005), and *SVP3* (Schilling et al., 2020). Gene names, synonyms, accession numbers, and orthologs in rice of the MADS-box genes analyzed in this study are provided in Supplemental Table S1.

Here, we show the complementary and overlapping roles of *SQUAMOSA* and* SVP* genes in the early reproductive phase, and their antagonistic effects during spikelet and initial floral development. We also show that constitutive expression of *VRT2* promotes leafy glumes and lemmas, likely by the downregulation of multiple MADS-box genes of the *SEPALLATA* (*SEP*) clade, which are known to be involved in floral development. Finally, we describe a complex network of interactions among wheat proteins from the SQUAMOSA, SVP, and SEP clades. The *SQUAMOSA* genes promote the downregulation of *SVP* genes, and this facilitates the interactions between SQUAMOSA and SEP proteins that are critical for normal spikelet and floral development.

## Results

### Quant-Seq analysis of developing wheat spikes in *vrn1* and *vrn1 ful2* mutants identifies genes regulated by *VRN1* and* FUL2*

**Figure 1:**
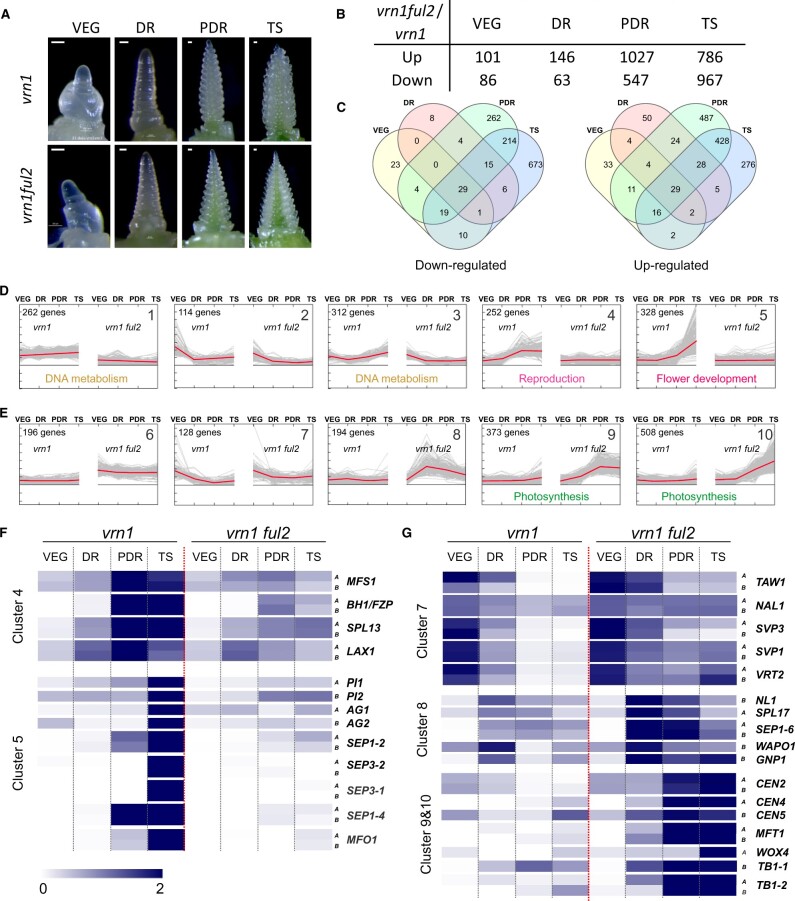
DEGs in developing spikes of *vrn1* and *vrn1 ful2* mutants. A, Representative images of vegetative (VEG) and reproductive apices (DR = double ridge; PDR = post double ridge; TS = terminal spikelet) from *vrn1* (upper row) and *vrn1 ful2* (lower row) collected for Quant-seq analysis. Bars = 100 μm. B, Number of DEGs between *vrn1* and* vrn1 ful2* identified at each stage. C, Venn diagrams showing the nonredundant DEGs in comparisons among the different developmental stages. Downregulated (left) and upregulated (right). D and E, Mean normalized expression patterns for clusters of nonredundant DEGs in *vrn1* and *vrn1 ful2* samples. Red lines represent the mean of all DEGs in the cluster, light gray lines show individual DEGs. Significantly enriched GO terms were identified only for Clusters 1, 3, 4, 5, 9, and 10, and are presented below the curves. F, Heat map showing mean normalized expression of selected genes for Clusters 4 and 5. G, Heat map showing the relative expression patterns of selected genes for Clusters 7, 8, 9, and 10. In the scale, 0–2 indicates mean normalized expression. A full list of DEGs for the 10 clusters is presented in Supplemental Data Set S1, and a description of DEGs with known functions in inflorescence development in Supplemental Table S3.

During early spike development, the IM in the *vrn1* mutant produces axillary meristems that acquire SM identity and develop into spikelets, whereas in the *vrn1 ful2* mutant, the IM produces axillary VMs that later develop into tillers subtended by a bract (Li et al., 2019). To identify the genes and pathways that repress the vegetative program and activate the spikelet identity program, we compared the transcriptomes of developing apices of these two mutants at four developmental stages covering the early steps of spike development: vegetative (VEG), double ridge (DR), post double ridge (PDR) and TS (Figure 1A). The average number of unique reads per sample and other transcriptome statistics are summarized in Supplemental Table S2.

In the comparisons between developing spikes of *vrn1* and* vrn1 ful2* mutants at the VEG and DR stages, we found 187 differentially expressed genes (DEGs; 86 downregulated and 101 upregulated) and 209 DEGs (63 downregulated and 146 upregulated), respectively (Figure 1B; Supplemental Data Set S1). These numbers greatly increased in the PDR and TS stages to 1,574 and 1,753 DEGs, respectively (Figure 1B).

We then performed a cluster analysis of the 1,399 upregulated and 1,268 downregulated nonredundant DEGs (Figure 1C) based on their expression profiles across the four developmental stages. This analysis resulted in five clusters for each of the two sets, which included at least 10% of the up- or downregulated genes (Figure 1, D and E; Supplemental Data Set S1). Clusters 4 and 5 included genes that were upregulated in *vrn1* but not in *vrn1 ful2* at PDR (Cluster 4) or TS (Cluster 5). The gene ontology (GO) analysis of these clusters revealed an enrichment of genes involved in early reproductive development in Cluster 4 and in flower development in Cluster 5, including four genes of the *SEP* clade (Figure 1, D and F). These results are consistent with the reproductive fate of the SM in *vrn1* relative to *vrn1 ful2*, where they develop into tiller-like structures. Additional genes from these clusters with known roles in inflorescence development are described in Supplemental Table S3.

We observed the opposite profiles in Clusters 9 and 10, which included genes upregulated in *vrn1 ful2* but not in *vrn1* between DR and PDR (Cluster 9) or at TS (Cluster 10) (Figure 1, E and G). The GO analysis of these clusters revealed an enrichment for genes involved in photosynthesis (Figure 1E), which is consistent with the vegetative fate of the *vrn1 ful2* spike axillary meristems. These clusters also include florigen antagonists *CENTRODIALIS 2* (*CEN2*), *CEN4*, and *CEN5* (Figure 1G). Cluster 8 showed a peak at the DR stage and included several genes previously shown to be involved in the regulation of spikelet number per spike (SNS; Figure 1G; Supplemental Table S3).

Because we were particularly interested in negative regulators of SM identity, we also analyzed genes from Cluster 7, which were highly downregulated between DR and PDR and at TS in *vrn1* but not in *vrn1 ful2* (Figure 1, E and G; Supplemental Table S3). This cluster included three MADS-box genes of the *SVP-*clade, confirming a previously published quantitative reverse transcription polymerase chain reaction (qRT-PCR) result showing significantly lower *VRT2*, *SVP1*, and *SVP3* transcript levels in *vrn1* relative to *vrn1 ful2* at the TS stage (Li et al., 2019). Of the three wheat genes in the *SVP*-clade, we prioritized the functional characterization of *VRT2* and *SVP1* because of their higher expression levels relative to *SVP3* at the PDR and TS stages (Figure 1G), and also because of their closer evolutionary relationship relative to *SVP3* (Supplemental Figure S1).

### Identification and combination of loss-of-function mutants for *VRT2* and *SVP1* in tetraploid wheat (*Triticum turgidum* L. subsp. *durum* (Desf.) Husn.)

**Figure 2:**
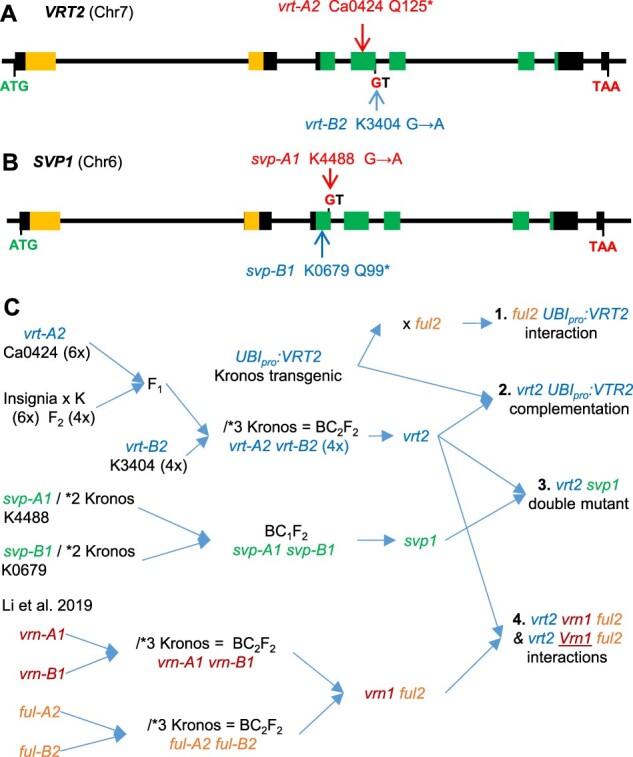
Selected *VRT2* and *SVP1* mutations and crosses with *vrn1* and *ful2* mutants, and transgenic *UBI_pro_:VRT2* plants. A–B, Locations of the selected mutations in *VRT2* (A) and *SVP1* (B) are shown in the gene structure diagram. Thick horizontal lines represent introns. Exons are represented by rectangles, with those in orange encoding the MADS domain and those in green encoding the conserved K domain. Ca = hexaploid wheat Cadenza and K = tetraploid wheat Kronos. GT is the mutated splice site. For both genes, the A genome mutants are indicated above the gene structure diagram and the B genome mutants below. C, Reference map of the crosses used to generate *vrt2* and *svp1* loss-of-function mutants and the higher-order mutants used in this study: 1. Interaction between a transgene with constitutive *VRT2* expression (*UBI_pro_:VRT2*) with the *ful2* mutant. 2. Complementation of *vrt2* with *UBI_pro_:VRT2*. 3. Generation of a *vrt2 svp1* double mutant. 4. Interactions between *vrt2* and *vrn1 ful2* mutants generated by Li et al. (2019). *Vrn1 =* heterozygous for *Vrn-A1*. The symbol “/*N” indicates the number of crosses to Kronos recurrent parent performed to reduce background mutations.

We selected truncation mutations for the A and B genome homeologs of *VRT2* and *SVP1*, which are summarized in Figure 2, A and B, respectively (for more detail see “Materials and methods”). To generate the *VRT2* loss-of-function mutant, designated hereafter as *vrt2*, we combined the premature stop codon mutation Q125* in the A-genome homeolog (*vrt-A2*) with a splice site mutation in the B-genome homeolog (*vrt-B2*; Figure 2A). This *vrt-B2* mutation results in splice variants with premature stop codons or a large deletion in the middle of the protein (Supplemental Figure S2). To generate the *SVP1* loss-of-function mutant, designated hereafter as *svp1*, we intercrossed an *svp-A1* mutant carrying a splice site mutation that generates splice variants with premature stop codons (Supplemental Figure S2) with an *svp-B1* mutant carrying the premature stop codon Q99* (Figure 2B). We generated PCR markers for each of these four mutations to trace them in the different crosses and backcrosses (Supplemental Table S4).

The selected *vrt2* and *svp1* mutants are likely loss-of-function mutants (or severely hypomorphic mutants) because the encoded proteins have truncations that eliminate more than half of the conserved K domain or, for one of the alternative splice forms of *svp-A1*, a protein with a large deletion including parts of the MADS and K domains (Figure 2B; Supplemental Figure S2). Figure 2C presents the crosses and backcrosses used to generate *vrt2*, *svp1*, *vrt2 svp1*, and the higher-order mutants described in other sections of this study.

### The *vrt2* and *svp1* mutations delay heading time, reduce plant height, and increase number of spikelets per spike

**Figure 3:**
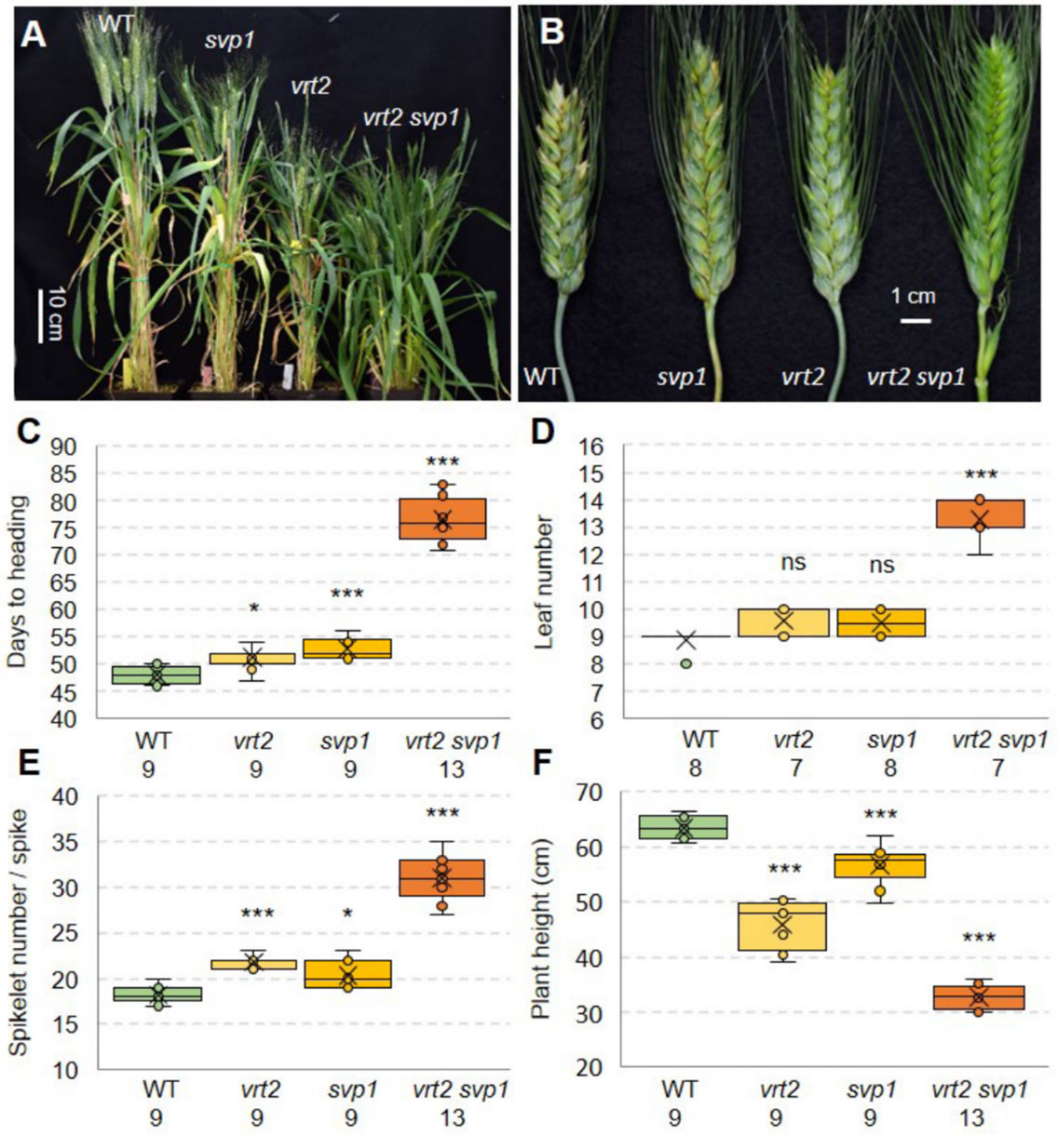
Effects of individual and combined *vrt2* and *svp1* mutants on important agronomic traits. A–F, Visual and quantitative phenotypes of WT, *vrt2*, *svp1*, and *vrt2 svp1* mutant plants. A, Plants 80 days after planting. Bar = 10 cm. B, Spikes (note the axillary spikelet in the first node of *vrt2 svp1*). Bar = 1 cm. C, Days to heading. D, Leaf number. E, SNS. F, Plant height (cm). C–F, The number of plants analyzed is indicated below the genotypes. *P < 0.05, ***P < 0.001 for differences with WT using Dunnett’s test (Supplemental Data Set S2). Box-plot features are explained in the “Statistical analyses” section of “Materials and methods.”

Plants homozygous for *vrt2* or *svp1* mutations were shorter, flowered later, and had higher SNS than the wild-type (WT), and all these effects were magnified in the *vrt2 svp1* mutant (Figure 3, A and B). Statistical analyses of these traits showed that *vrt2* and *svp1* mutants headed significantly later than WT in two independent experiments (2.9–4.8 days, Figure 3C;  Supplemental Figure S3, A and B; Supplemental Data Set S2), indicating a small but consistent effect of both genes as promoters of heading time. In *vrt2 svp1*, the delay in heading time (29 days) was much larger than the sum of the individual gene effects, reflecting a highly significant interaction between these two genes (Supplemental Table S5). Mutations in the individual A- and B-genome homeologs of each gene showed no significant differences in heading time (Supplemental Figure S3).

The late heading time of the *vrt2 svp1* mutant was correlated with a highly significant increase in leaf number (4.4 more leaves than WT, *P* < 0.001, Figure 3D), which indicates that part of the delay in heading time was caused by a delayed transition of the shoot apical meristem (SAM) from the vegetative to the reproductive stage. Using qRT-PCR, we detected reduced expression levels of the flowering promoting genes *VRN1* and* FT1* and higher levels of the flowering repressor *VRN2* in the fifth leaf of the *vrt2 svp1* mutants compared with the WT (Supplemental Figure S4; Supplemental Data Set S2).

Both *vrt2* and* svp1* produced significantly more spikelets per spike than the WT under controlled environmental conditions, with larger differences between the *VRT2* alleles (3.6–4.9 spikelets) than between the *SVP1* alleles (2.1–2.9 spikelets; Figure 3E;  Supplemental Figure S3, C and D). The increase in SNS in the *vrt2 svp1* mutant relative to the WT (13 spikelets or ∼70% increase) was larger than the added differences of the individual mutants, indicating a highly significant interaction (Supplemental Table S5). Mutants for the A- and B-genome homeologs of both genes showed significant effects on SNS under controlled environments, and these effects were validated for *vrt2* in the field (Supplemental Figure S3, C, D, and G; Supplemental Data Set S2).

We also observed significant reductions in plant height in *vrt2*, *svp1*, and *vrt2 svp1* mutants (Figure 3F), and detected a significant interaction between the two genes (*P* *=* 0.0032, Supplemental Data Set S2). The effect of *vrt2* on plant height (−17.5 cm) was stronger than that of *svp1* (−6.8 cm, Figure 3F), and both were determined mainly by reductions in peduncle length (Supplemental Figure S3, E and F; Supplemental Table S5). In a field experiment, the effect of *vrt2* on plant height was even stronger than in the controlled environment experiments (−28.4 cm; Supplemental Figure S3, H and I; Supplemental Data Set S2).

In summary, these results indicate that *VRT2* and* SVP1* have overlapping functions during early reproductive development in wheat, accelerating the transitions from VM to IM and from IM to TS, and promoting elongation of the peduncle.

### The *vrt2 svp1* mutant has axillary spikes at the nodes of the elongating stems

**Figure 4:**
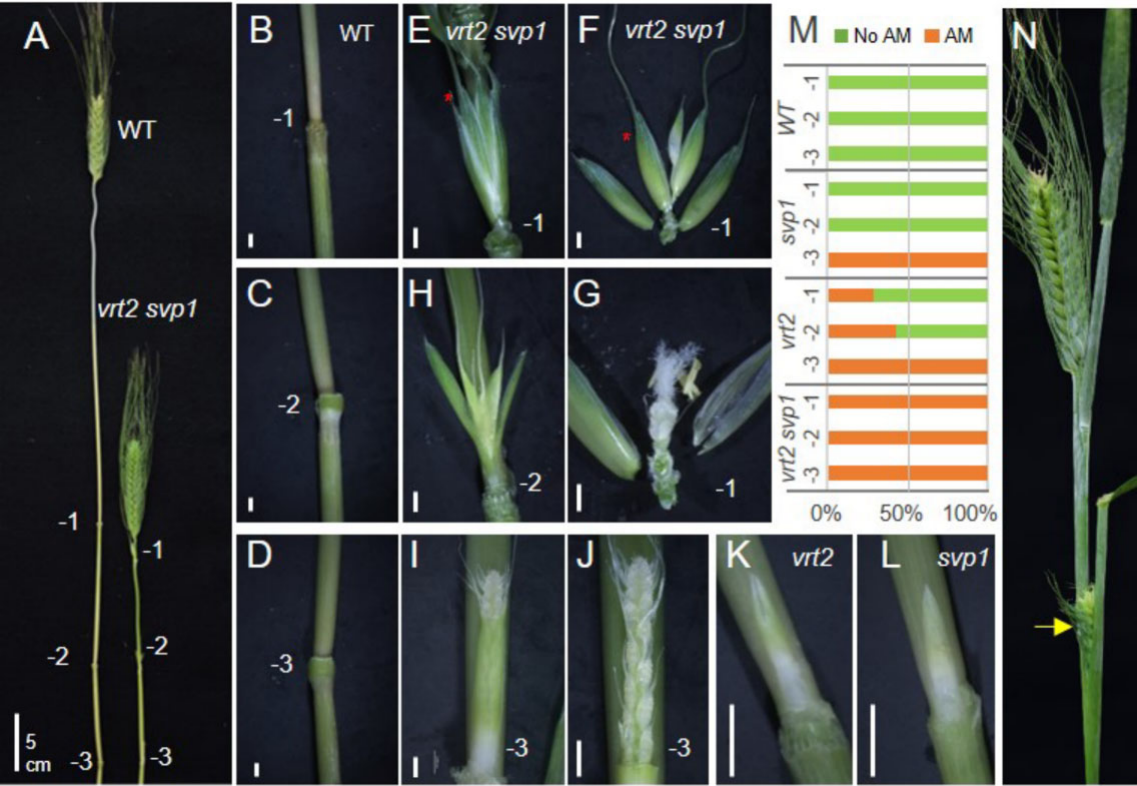
Axillary inflorescences in *vrt2, svp1*, and *vrt2 svp1*. A, Comparison of internodes in WT and *vrt2 svp1* (−1 is the node below the peduncle, and −3 is the most basal node). B–D, Detail of the three nodes in WT. E–J, Nodes in *vrt2 svp1.* E, Spikelet in node −1. F, Dissection of the spikelet showing glumes and three florets. G, Dissection of floret one (red star in (F)) showing normal floral organs. H, Spikelet in node −2. I, Axillary spike in node −3 surrounded by a bract. J, Same axillary spike without the bract showing lateral spikelets. K, Axillary spike in *vrt2* surrounded by a bract*.* L, Axillary spike in *svp1* surrounded by a bract*.* M, Proportion of plants with axillary spikes or spikelets in each of the three nodes below the spike (*n* = 7). Green = axillary meristem (AM) absent or not developed, Orange= axillary meristem developed into a spike or spikelet. N, The yellow arrow points to an axillary spike emerging from its subtending leaf in *vrt2 svp1*. Bars in (A) = 5 cm, (B–L) = 2 mm.

A surprising characteristic of the *vrt2 svp1* mutant was the presence of axillary spikelets or spikes subtended by leaves in the nodes of the elongating stem below the peduncle (Figure 4A). Although axillary inflorescences are common in some species from other grass subfamilies including the Bambusoideae, Andropogoneae, and Panicoideae (Stapleton, 1997; Vegetti, 1999), WT wheat does not have axillary spikelets, spikes, or tillers in the nodes of the elongating stem (Figure 4, B–D).

We observed a gradient in the development of the axillary buds, with those in node −1 closest to the spike developing into a single spikelet (Figure 4E), those in node −2 into one or two spikelets, and those in node −3 into normal spikes with multiple spikelets (Figure 4, I, J, and N). The single axillary spikelets in node −1 showed normal spikelet and floral characteristics (Figure 4, F and G). The axillary spikes in node −3 were initially enclosed by one bract (Figure 4I). Upon removal of these bracts, we observed normally developing spikes (Figure 4J), which were delayed in their development relative to the corresponding apical spikes at the same time point (Figure 4A). These unusual axillary spikelets and spikes were also observed in some nodes of *vrt2* (Figure 4K) and *svp1* (Figure 4L) individual mutants, but at lower frequencies (Figure 4M) and were usually less developed than in the *vrt2 svp1* mutant. In *vrt2 svp1* mature plants, some axillary spikes were able to emerge from the subtending leaves (Figure 4N, yellow arrow).

### Expression patterns of *VRT2* and *SVP1* in wheat inflorescences correlate with mutant phenotypes

To further characterize *VRT2* and* SVP1* genes, we performed *in situ* hybridization at different stages of spike development. We also included *VRN1* and* FUL2* in our *in situ* hybridization analyses to compare the expression patterns of *SVP* and *SQUAMOSA* genes. In Kronos, *VRN1* and* FUL2* expressions were detected in leaf primordia and in the SAM. During inflorescence development, both genes were expressed in the IM, in the emerging SM and in the subtending vegetative ridge (Supplemental Figure S5) supporting their roles in spikelet development, repression of the vegetative ridge, and determinacy of the IM (Li et al., 2019). Similar expression patterns were observed in diploid *Triticum* *monococcum* L. (Supplemental Figure S5, D and I).

**Figure 5:**
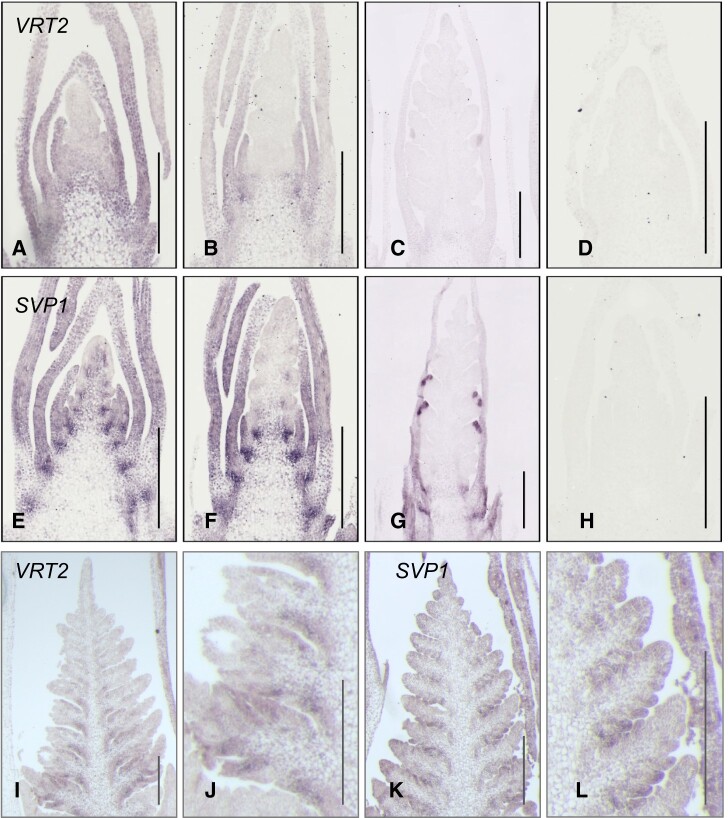
*In situ* hybridization analysis of *VRT2* and *SVP1* in developing Kronos inflorescences*.* A–H, WT Kronos, early spike development stages before TS formation. A, D, E, H = early DR; B, F = DR and C, G = TS. I–L, Kronos *vrn1 ful2* mutant inflorescence with lateral VMs. The developmental stage is equivalent to TS in WT, but without TS. J and L are amplified regions from I and K, respectively. A–D and I–J, *VRT2.* E–H and K–L, *SVP1.* D and H, Control sense probes for *VRT2* and *SVP1*, respectively. A–H, *Triticum monococcum* probes. I–L, Kronos probes. Primers for the probes are described in Supplemental Table S4. Bars = 500 μM.

In agreement with the expression profile observed in the Quant-Seq analysis for the *SVP-*clade genes (Figure 1G), the *in situ* hybridizations with *VRT2* and* SVP1* revealed a progressive decrease of signal in the IM and SM with spike development (Figure 5, A–C and E–G). When spikelets reached the stamen primordia stage, we observed a strong signal in the stamen primordia for *SVP1* (central spikelets develop earlier in wheat; Figure 5G), but not for *VRT2* (Figure 5C). This latter result is consistent with the expression pattern reported for the *SVP1* homolog *OsMAD22* in rice (Pelucchi et al., 2002; Sentoku et al., 2005).

Both *SVP1* and* VRT2* showed hybridization signal in leaf primordia below the developing spikes, with a stronger signal at the base of these organs. Similar expression profiles were detected in Kronos (Figure 5, A–C and E–G) and *T. monococcum* (Supplemental Figure S6)*.* In contrast to WT Kronos, the *vrn1 ful2* mutant showed ectopic expression of *VRT2* and *SVP1* at later stages of spike development, a result consistent with the Quant-Seq data (Figure 1G). This ectopic expression was concentrated in the spike axillary organ primordia that then develop into tiller-like structures (Figure 5, I–L).

Similar to *VRT2* and *SVP1*, the *CEN2*, *CEN4*, and *CEN5* genes showed higher expression levels after DR in *vrn1 ful2* than in *vrn1* in the Quant-Seq analysis (Figure 1G). Therefore, we investigated the expression profiles of *CEN2* in WT and *vrn1 ful2* mutant by *in situ* hybridization. We selected *CEN2* because it was expressed at higher levels than *CEN4* and *CEN5* in the developing spikes. In the WT Kronos, *CEN2* showed strong hybridization signals at the base of the leaves of the early developing spikes, similar to *VRT2* and *SVP1* (Supplemental Figure S7, A–D). In the *vrn1 ful2* mutant, we observed ectopic expression of *CEN2* in the spike axillary organ primordia, similar to what we observed for *VRT2* and *SVP1* (Figure 5, I–L; Supplemental Figure S7E), suggesting that both *SVP* and *CEN* genes may contribute to the leafy characteristics of the spike axillary meristems in this mutant.

### Constitutive expression of *VRT2* alters spike development

**Figure 6:**
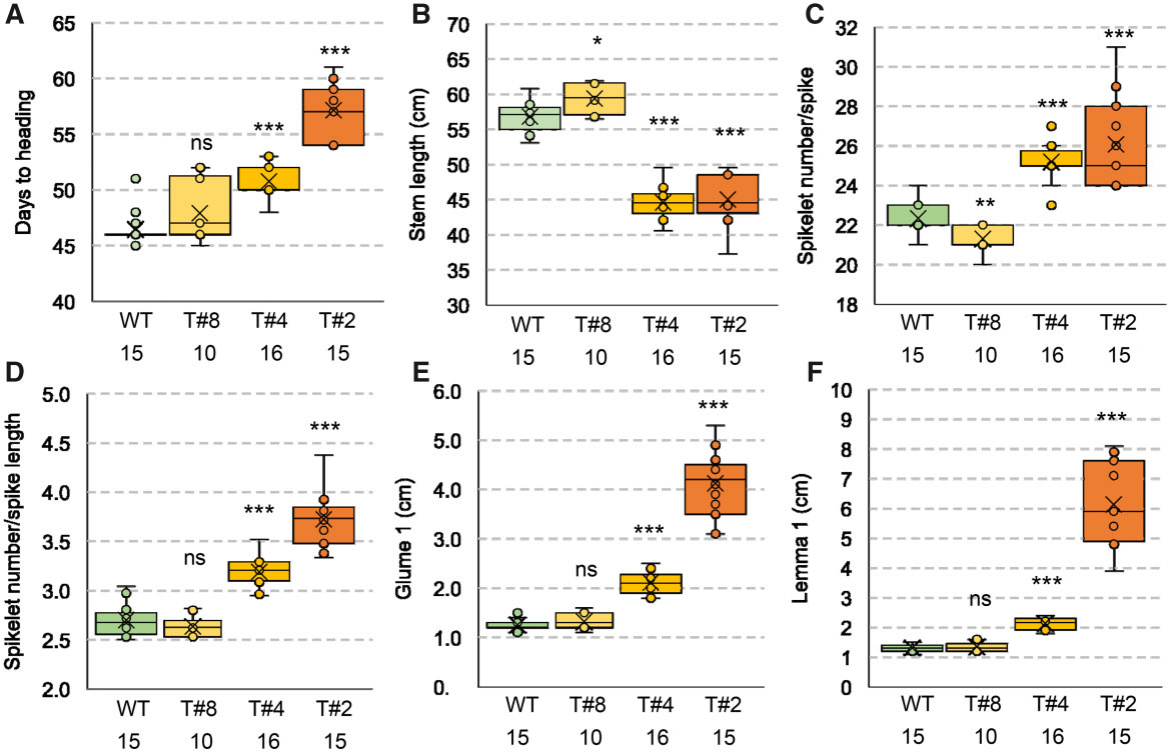
Phenotypic characterization of Kronos lines constitutively expressing *VRT2*. Three independent *UBI_pro_:VRT2* events with weak (T#8), intermediate (T#4), and strong phenotypes (T#2). A, Days to heading. B, Stem length (without spike). C, SNS. D, Spikelet density (spikelet number/spikelet length in cm). E, Glume 1 length. F, Lemma 1 length. A–F, The number of plants analyzed is indicated below the genotypes. *P *<* 0.05, **P *<* 0.01, *** *P* *<* 0.001 in Dunnett’s test versus WT control (Supplemental Data Set S2). Box-plot features are explained in the “Statistical analyses” section of “Materials and methods.”

**Figure 7:**
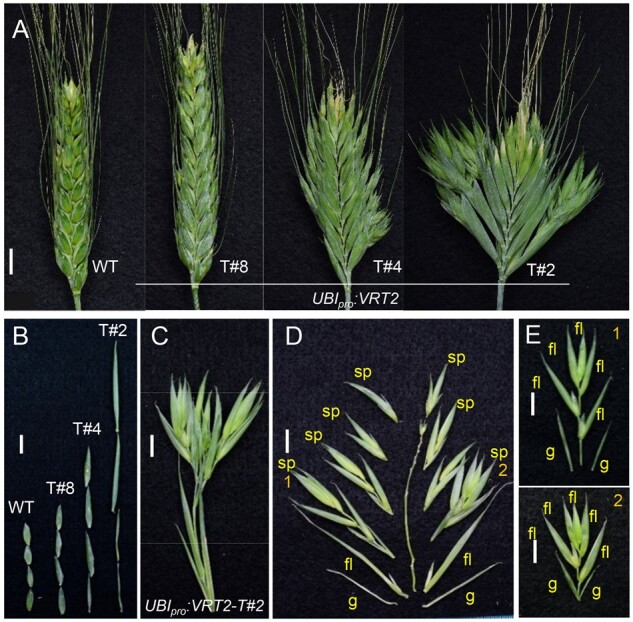
Spikes and spikelets changes in Kronos lines constitutively expressing *VRT2*. Three independent events with weak (T#8), intermediate (T#4), and strong phenotypes (T#2). A, Spike phenotype. B, Aligned glumes showing difference in length. C, Basal “spikelet” from *UBI_pro_:VRT2* event T#2. D, Dissection of the basal “spikelet” shows a determinate branch with multiple spikelets. E, Detail of spikelets 1 and 2 in (D), each with glumes, florets and an elongated rachilla. Bars = 1 cm. g = glume, fl= floret and sp= spikelet.

**Figure 8:**
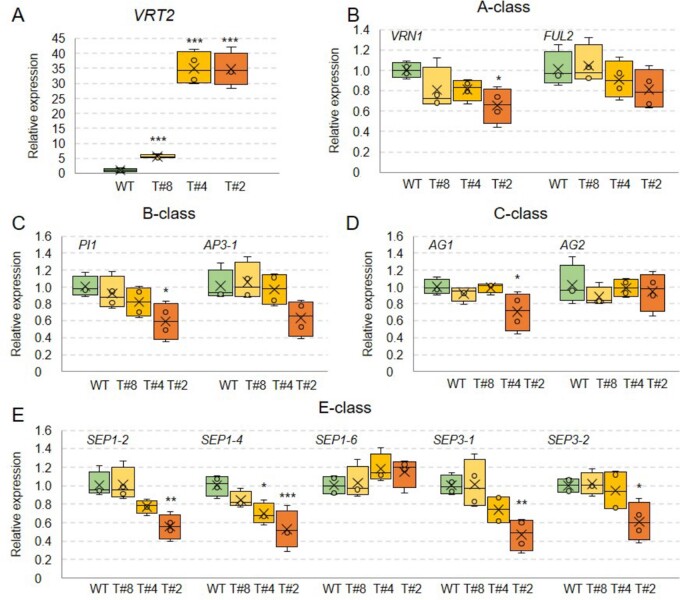
Relative expression of wheat flowering genes in developing spikes at the TS stage of *UBI_pro_:VRT2* transgenic lines T#8, T#4, and T#2 and sister lines without the transgene (WT). A, *VRT2* (transgenic plus endogenous transcripts). B, A-class MADS-box genes *VRN1* and *FUL2*. C, B-class MADS-box genes *PI1* (∼*OsMADS4*) and *AP3-1* (∼*OsMADS16*). D, C-class MADS-box genes *AG1* (∼*OsMADS58*) and *AG2* (∼*OsMADS3*). E, E-class MADS-box genes *SEP1-2* (∼*OsMADS1*), *SEP1-4* (∼*OsMADS5*), *SEP1-6* (∼*OsMADS34*), *SEP3-1* (∼*OsMADS7*) and *SEP3-2* (∼*OsMADS8*). A–E, Graphs are based on four biological replicates (each replicate is a pool of 6–8 developing spikes at the TS stage). *P < 0.05, **P < 0.01, ***P < 0.001 in Dunnett’s test versus the WT control (Supplemental Data Set S2). Expression was determined by qRT-PCR using *ACTIN* as endogenous controls and normalization relative to the WT (WT = 1). Box plot features are explained in the “Statistical analyses” section of “Materials and methods.”

To test whether the ectopic expression of *SVP*-like genes observed in the *vrn1 ful2* mutant contributes to the vegetative characteristic of its inflorescences, we generated transgenic plants constitutively expressing *VRT-A^m^2* (cloned from *T. monococcum* A^m^ genome) under the maize *UBIQUITIN* promoter (hereafter referred to as *UBI_pro_:VRT2*). We characterized three independent transgenic events (T#2, T#4, and T#8) that displayed varying degrees of phenotypic effects (Figures 6 and 7). The intensity of these phenotypic defects was partially correlated with *VRT2* transcript levels in their developing spike at the TS stage. Transgenic lines T#2 and T#4 showed higher transcript levels than T#8, and all three had transcript levels significantly higher (*P* < 0.0001) than the nontransgenic sister lines (WT, Figure 8A).

The T#8 plants showed no significant differences in heading time, whereas the T#4 and T#2 plants headed 4.3 and 10.7 days later than the WT, respectively (*P* < 0.001, Figure 6A). Interestingly, in this experiment, T#8 showed small but opposite effects to T#4 and T#2 for both stem length (Figure 6B) and SNS (Figure 6C). Relative to the WT, T#8 showed a significantly longer stem (2.6 cm. *P* *=* 0.008) and reduced SNS (1 spikelet, *P* *=* 0.0015), while both T#4 and T#2 showed shorter stems (12.2 and 11.8 cm shorter, *P* < 0.001, Figure 6B) and higher SNS (2.9 and 3.7 more spikelets, respectively, *P* < 0.001, Figure 6C). The increase in SNS in T#4 and T#2 resulted in significant increases in spikelet density (Figure 6D). All three transgenic lines showed longer glumes and lemmas than the WT, but the differences were significant (*P* < 0.001) only for T#4 and T#2 (Figure 6, E and F). T#2 exhibited the most severe morphological alterations (Figure 7, A–E), including very long glumes and lemmas, and replacement of basal spikelets by branches with multiple spikelets (also with elongated glumes and lemmas, Figure 7, C–E).

We explored the ability of the weakest *UBI_pro_:VRT2* transgenic line (T#8) to complement the morphological changes observed in *vrt2* in the F_2_ progeny of a cross between T#8 and *vrt2* (Figure 2C). In the absence of the transgene, the *vrt2* mutant headed 3.2 d later than the WT, but those differences disappeared in the presence of the transgene, indicating full complementation (Supplemental Figure S8A). The differences in peduncle length between the WT and *vrt2* mutant (19.6 cm) were significantly reduced in the presence of *UBI_pro_:VRT2* (11.4 cm, Supplemental Figure S8B), indicating partial complementation. However, there was no complementation for the differences in SNS, with similar increases in SNS in the *vrt2* mutant relative to the WT in the transgenic and nontransgenic backgrounds (Supplemental Figure S8C).

To understand better the effect of *UBI_pro_:VRT2* on the regulation of spikelet development, we used qRT-PCR to compare the transcript levels of several MADS-box flowering regulators between the three *UBI_pro_:VRT2* transgenic and the nontransgenic sister line at the TS stage (Figure 8). We observed significant reductions in the transcript levels of A-class gene *VRN1*, B-class gene *PI1*, C-class gene *AG1*, and E-class genes *SEP1-2*, *SEP1-4*, *SEP3-1*, and *SEP3-2* (Figure 8, B–E) in the strongest T#2 transgenic wheat line. The downregulation of these flowering regulators was correlated with the spike phenotypic changes in the different transgenic events, and was significant in the T#4 transgenic line only for *SEP1-4*, and not significant for all the studied genes for T#8 (Figure 8, B–E).

### The *ful2* mutant enhances spikelet defects in weak *UBI_pro_:VRT2* transgenic plants

Strong constitutive expression of *VRT2* resulted in spikelets with leaf-like glumes and lemmas similar to those observed in the partial mutants carrying one functional copy of *VRN-A1* in the heterozygous state (*Vrn1*) and no functional copies of *FUL2* (*ful2*), which was previously designated as *Vrn1 ful2* (Li et al., 2019). Based on these results, we hypothesized that *VRT2* and *FUL2* may have opposite effects on spikelet development. To test if the combination of *ful2* and *UBI_pro_:VRT2* would enhance the spike and spikelet defects of the individual lines, we crossed the weak *UBI_pro_:VRT2* T#8 with *ful2*.

**Figure 9:**
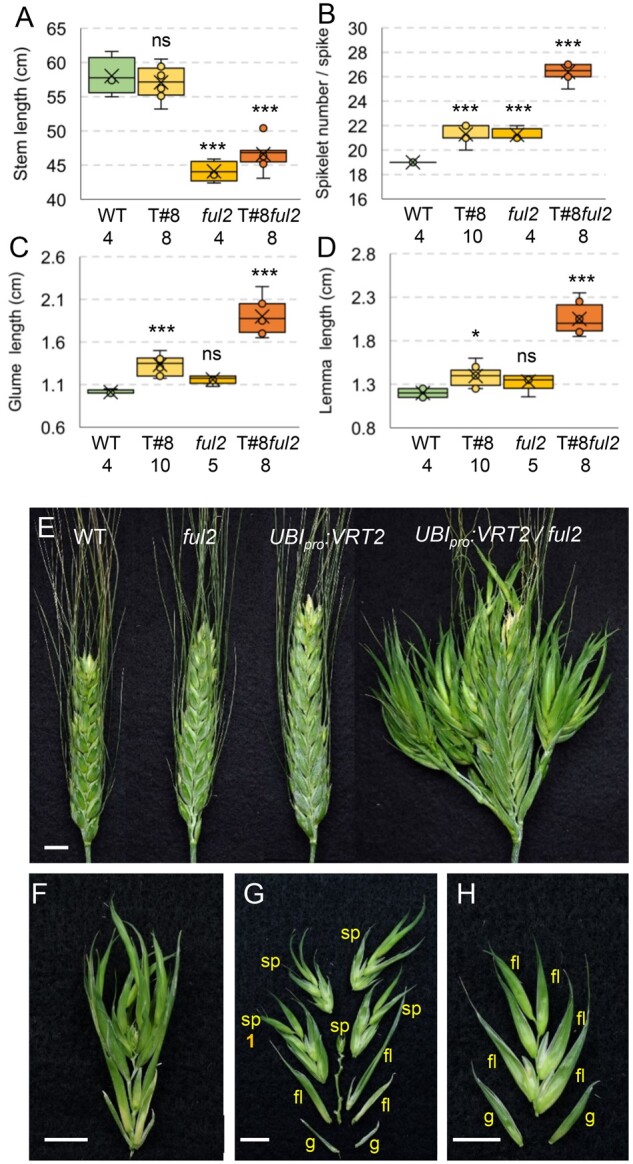
Effect of combined *ful2* mutation and *UBI_pro_:VRT2* T#8 transgenic line on stem elongation and spike/spikelet development. A, Stem length (without spikes). B, SNS. C, Glume length in centimeter. D, Lemma length in centimeter. A–D, WT = homozygous *Ful2* and not transgenic. T#8: weak constitutive transgenic *UBI_pro_:VRT2* line T#8. *ful2* = loss-of-function mutant for *ful-A2* and *ful-B2* and not transgenic. T#8*ful2 =* homozygous *ful2* and T#8 transgenic present. *N* = number of plants analyzed is indicated below the genotypes. *P *<* 0.05, **P *<* 0.01, ***P *<* 0.001 in Dunnett’s test (Supplemental Data Set S2). Box-plot features are explained in the “Statistical analyses” section of “Materials and methods.” E, Young spikes of WT Kronos, *ful2*, *UBI_pro_:VRT2* T#8, and combined *UBI_pro_:VRT2* T#8 – *ful2*. F, Combined *UBI_pro_:VRT2 ful2* basal spikelet transformed into a branch. G, Dissection of the basal spikelet converted into a branch showing lateral spikelets. H, Dissection of the spikelet marked with 1 in G. Bars = 1 cm.

The differences in stem length were highly significant for *ful2* but not for *UBI_pro_:VRT2* (Figure 9A; Supplemental Data Set S2). In contrast, SNS, glume length, and lemma length were significantly affected by both *ful2* and* UBI_pro_:VRT2*, and highly significant interactions were detected for all three traits (Supplemental Data Set S2; Figure 9, B–D). Plants combining *UBI_pro_:VRT2* T#8 and *ful2* showed long leaf-like glumes and lemmas, similar to those of the strong transgenic lines T#4 and T#2 and a large increase in floret number (Figure 9E), a phenotype reported previously in *Vrn1 ful2* mutant plants (Li et al., 2019). Dissection of basal spikelets showed that some florets were replaced by spikelets and that the rachilla ended in a TS (resembling a determinate branch) in seven of the eight plants analyzed (Figure 9, F–H). Taken together, these results suggest that *VRT2* and* FUL2* have antagonistic effects on spikelet development.

### The *vrt2* mutant reduces spikelet developmental defects in the *Vrn1 ful2* mutant

Because *VRT2* constitutive expression exacerbated the spikelet defects observed in the *ful2* mutant, and *VRT2* and *SVP1* are ectopically expressed in *vrn1 ful2* “spikelets” (Figure 5, I–L), we hypothesized that the loss-of-function of *VRT2* could reduce some of the vegetative characteristics of the *vrn1 ful2* “spikelets.” To test this hypothesis, we crossed *vrt2* with *Vrn1 ful2* and selected two pairs of sister lines, the first one homozygous for *vrn1* (*vrt2 vrn1 ful2* and* vrn1 ful2*) and the second one with one functional copy of *Vrn-A1* (*vrt2 Vrn1 ful2* and *Vrn1 ful2*).

The *vrn1 ful2* mutant plants were taller than the *vrt2 vrn1 ful2* mutant plants (Supplemental Figure S9, A–B), and most of its shoots produced spikes with axillary spikelets replaced by tillers (Supplemental Figure S9C), as described in a previous study (Li et al., 2019). In contrast, 84% of the inflorescences in the *vrt2 vrn1 ful2* mutant failed to emerge (Supplemental Figure S9D). Spikes that emerged from the other 16% shoots showed deformed axillary tillers replacing the spikelets (Supplemental Figure S9, E–F). Dissection of the shoots from *vrn1 ful2* (Supplemental Figure S9G) and *vrt2 vrn1 ful2* (Supplemental Figure S9H) revealed that *vrt2 vrn1 ful2* had underdeveloped spikes with very short peduncles and internodes, most of which eventually died within the sheaths.

Scanning electron microscope (SEM) images of the developing inflorescences at the PDR stage showed that, in both *vrt2 vrn1 ful2* and* vrn1 ful2*, the spike axillary meristems resembled VMs, some of them bearing axillary buds in the first leaf-like primordia (Supplemental Figure S10, A–D). In the *vrn1 ful2* SEM images, the visible buds were undifferentiated (yellow arrows). Dissection of *vrn1 ful2* mature tiller-like organs revealed modified floral organs (Li et al., 2019), suggesting that these buds have the potential to eventually differentiate. Interestingly, in the SEM images of *vrt2 vrn1 ful2*, we observed more developed floret organ primordia (orange arrows) in the axils of some basal leaf-like primordia, indicating that *vrt2* mutation may reduce “spikelet” defects of *vrn1 ful2* mutants.

**Figure 10:**
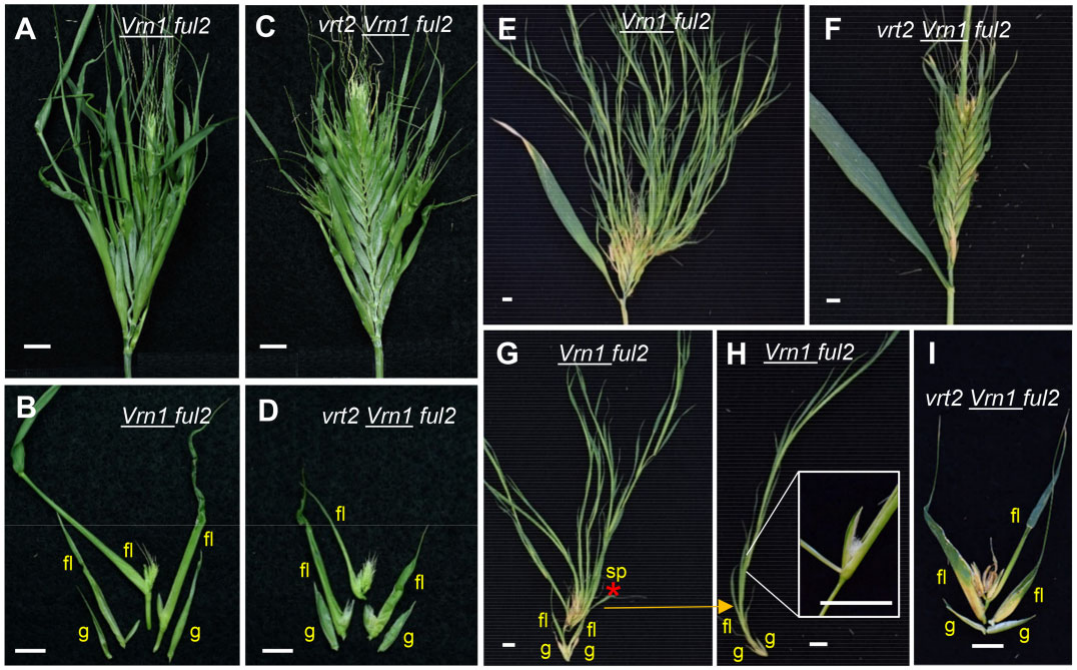
Effect of combined *Vrn1 ful2* and *vrt2* mutations on spike and spikelet development. A, B, E, G–H, *Vrn1 ful2.* C, D, F, I *vrt2 Vrn1 ful2.* A and C, Young spikes. B and D, Dissection of basal spikelets. E and F, Older spikes. G–I Dissection of older spikelets. H, Detail of the third “floret” in G (red asterisk) that reverted to a spikelet with its own glumes. The inset in (H) shows a floret of this spikelet. I, Spikelet of the same age as in G from *vrt2 Vrn1 ful2.* Bars = 1 cm.

To better visualize the effect of the *vrt2* mutation on spikelet development, we compared sister lines *Vrn1 ful2* and *vrt2 Vrn1 ful2* with one functional copy of *Vrn-A1* (Figure 10; Supplemental Figures S10 and S11). SEM images of the early developing inflorescences showed no clear differences, with both genotypes bearing axillary SMs showing normal floret organ primordia (Supplemental Figure S10, E–H). However, clear morphological differences between these genotypes occur at later developmental stages.

The *vrt2 Vrn1 ful2* mutant headed 3.5 days later (Supplemental Figure S11A), had shorter stems (Supplemental Figure S11B, not significant [NS]), and produced on average 4.7 more spikelets per spike (Supplemental Figure S11C) than *Vrn1 ful2*. The spikes of *Vrn1 ful2* showed long glumes and lemmas (Figure 10B; Supplemental Figure S11, D and E), an unusually high number of florets (Figure 10E) and branches replacing 80% of the basal spikelets (Figure 10, G–H; Supplemental Figure S11F). In contrast, the spikes of *vrt2 Vrn1 ful2* showed significantly shorter glumes and lemmas (Figure 10D; Supplemental Figure S11, D and E), a more normal number of florets (Figure 10F), and a reduced proportion of basal spikelets replaced by “branches” (30%, Supplemental Figure S11F). In summary, the spikes and spikelets of *vrt2 Vrn1 ful2* appeared more normal than in *Vrn1 ful2* (Figure 10, E–F), despite the presence of lemmas still showing some leafy characteristics (Figure 10I).

A qRT-PCR comparison of some of the DEGs identified in the Quant-Seq data showed significantly higher transcript levels of *SEPALATA* genes *SEP1-2* and *SEP3-1* and reduced expression of C*EN2*, *CEN5*, and *TB1-2* in the developing spikes of *vrt2 Vrn1 ful2* relative to *Vrn1 ful2* (Supplemental Figure S12A). The positive effect of the *SVP* genes on the transcript levels of C*EN2*, *CEN4*, *CEN5*, and *TB1-2* in developing spikes was validated in the *vrt2 svp1* mutants (Supplemental Figure S12B). These expression profiles are consistent with the changes observed between *vrn1* and* vrn1 ful2* apices (Figure 1, F and G). Taken together, the genetic interactions and the qRT-PCR results indicate that *VRT2* is partially responsible for the spikelet defects of the *Vrn1 ful2* mutant, possibly by regulating the expression of some of the DEGs identified in the Quant-Seq analyses.

### Wheat SQUAMOSA proteins interact with SVP and SEP MADS-box proteins

**Figure 11:**
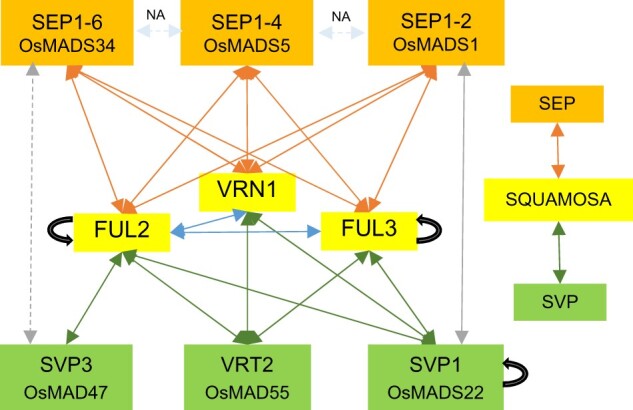
Y2H interactions between wheat SQUAMOSA, SVP, and SEP proteins. Wheat MADS-box proteins of the SQUAMOSA-clade are indicated by yellow boxes (VRN1, FUL2, and FUL3), proteins of the SVP-clade by green boxes (VRT2, SVP1, and SVP3) and proteins of the LOFSEP-clade by orange boxes (SEP1-2, SEP1-4, and SEP1-6). Positive interactions between SQUAMOSA and LOFSEP-clade proteins are shown with orange arrows, between SQUAMOSA- and SVP-clade proteins with green arrows, and between SVP- and LOFSEP-clade proteins in gray (the weak interaction between SVP3 and SEP1-6 is indicated by a dotted line). Black curved arrows indicate positive homodimerization. Interactions among SEP proteins were not analyzed.

Because the previous mutant analyses revealed genetic interactions among *SQUAMOSA*, *SVP*, and *SEP* genes, we decided to explore the pairwise physical interactions among the proteins encoded by these genes using yeast two-hybrid (Y2H) assays. After confirming that none of these wheat proteins caused autoactivation in Y2H assays (Supplemental Figure S13), we tested the interactions within the clades. Individual members of the SVP-clade did not interact with each other, and only SVP1 was able to form homodimers (Figure 11; Supplemental Figure S14). Among the SQUAMOSA proteins, FUL2 and FUL3 showed strong and weak homodimerization, respectively (Supplemental Figure S14). FUL2 interacted with both VRN1 and FUL3, whereas the latter two did not interact with each other (Supplemental Figure S14). Pairwise interactions between proteins from the two clades revealed that VRT2 and SVP1 interacted with all three SQUAMOSA proteins, whereas SVP3 interacted only with FUL2. The interactions of SVP1 with all three SQUAMOSA proteins were of similar strength but the VRT2 interaction was weakest with VRN1 and strongest with FUL2 (Supplemental Figure S15).

Rice SEP proteins of the LOFSEP subclade (OsMADS1, OsMADS5, and OsMADS34) are critical for spikelet and floret organ identity and have been shown to interact with SQUAMOSA proteins (Wu et al., 2018). In wheat, we observed positive Y2H interactions for all nine possible pairwise combinations of the three LOFSEP proteins with the three SQUAMOSA proteins (Figure 11). The interactions for all three LOFSEP proteins were strongest with FUL2, intermediate with FUL3 and weakest with VRN1 (Supplemental Figure S15). In contrast, there were fewer positive Y2H interactions between proteins of the LOFSEP and SVP clades. Among the nine possible pairwise combinations, we only detected a strong interaction between SEP1-2 and SVP1 and a weak interaction between SEP1-6 and SVP3. In summary, wheat proteins from the SQUAMOSA-clade interacted with most of the proteins from the SVP and LOFSEP clades in yeast, whereas the latter two showed limited interaction with each other (Figure 11).

We also used bimolecular fluorescence complementation (BiFC) to validate the positive Y2H interactions in wheat protoplasts (Supplemental Table S6). We observed fluorescent signals in the nucleus (and sometimes in the cytoplasm) for 9 of the 15 tested interactions (Supplemental Table S6; Supplemental Figure S16, A–I), whereas the six interactions of SEP1-4 and SEP1-6 with the SQUAMOSA proteins showed no nuclear fluorescence (Supplemental Figure S16, J–O). Some of the positive and negative interactions showed fluorescing protein aggregates outside the nucleus (Supplemental Figure S16, G and H, see footnote 1). We did not detect fluorescent nuclear signals or aggregates for the negative controls using the C-terminal part of the Yellow Fluorescent Protein (YFP-C) paired with individual proteins of all three clades fused to YFP-N (N-terminal part of YFP) (Supplemental Figure S16, P–W). The lack of nuclear fluorescence between the three SQUAMOSA proteins with SEP1-4 and SEP1-6 (Supplemental Table S6) served as additional negative controls for proteins of the same families.

### Wheat SVP and LOFSEP proteins compete for interactions with SQUAMOSA in yeast

**Figure 12:**
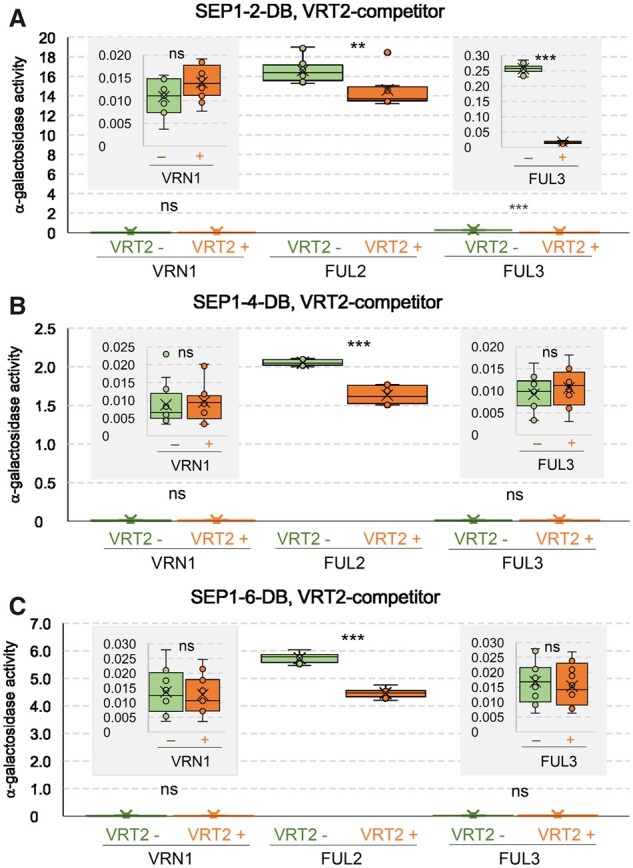
Wheat VRT2 competes with LOFSEP proteins for interactions with SQUAMOSA proteins in yeast. A–C, Y3H assays were used to test the effect of VRT2 as a competitor, where (A) SEP1-2 (∼OsMADS1), (B) SEP1-4 (∼OsMADS5), and (C) SEP1-6 (OsMADS34) were expressed as DNA-binding domain fusions, and SQUAMOSA proteins VRN1/FUL2/FUL3 were expressed as activation domain fusions. The α-gal activity of the protein interactions in the absence of the competitor is shown in green box plots and in the presence of the competitor in orange box plots. Relative α-gal activity values for each interaction are the average of 12 replicates. **P < 0.01 and ***P < 0.001 (paired *t* tests, Supplemental Data Set S2). The insets show the α-gal activity for weaker interactions using different scales. Box-plot features are explained in the “Statistical analyses” section of “Material and methods.”

Because both SVP and LOFSEP proteins interact with SQUAMOSA proteins and *VRT2* ectopic expression results in spikelet and floret defects, we then tested whether the presence of the SVP proteins could interfere with the interaction between the SQUAMOSA and LOFSEP proteins using yeast three-hybrid (Y3H) assays. The α-gal assays confirmed that all three LOFSEP proteins had much stronger interactions with FUL2 than with FUL3 or VRN1 (Figure 12). SEP1-4 and SEP1-6 showed interactions of similar strength with FUL3 and VRN1, but SEP1-2 interaction with FUL3 was stronger than with VRN1 (Figure 12). The expression of VRT2 as the competing protein in Y3H assays significantly reduced the α-gal activity of the three strong FUL2–LOFSEP interactions (12.0% in SEP1-2, 20.6% in SEP1-4, and 22.0% in SEP1-6, *P* < 0.01). Among the weak interactions, the presence of VRT2 only had a significant effect on the FUL3–SEP1-2 interaction (93.8% reduction, *P* < 0.0001, Figure 12). Taken together, these results indicate that the presence of wheat VRT2 can interfere with some of the wheat SQUAMOSA–LOFSEP interactions in yeast.

## Discussion

### Roles of *SQUAMOSA* genes in the transcriptome of developing spikes and spikelets in wheat

By comparing the transcriptomes of developing spikes from *vrn1 ful2* (in which spikelets are replaced by tillers) and *vrn1* (normal spikelets) at four developmental stages, we identified genes and pathways regulating spike and spikelet development (Figure 1). The dramatic increase in the number of DEGs between the DR and PDR stages suggests that this developmental interval is critical for the establishment of the different developmental fates of the axillary spike meristems in the *vrn1* and* vrn1 ful2* mutants. The lower number of DEGs in the VEG and DR stages of spike development correlates with the similar morphology of the early developing spikes of *vrn1* and *vrn1 ful2* mutants up to the DR stage (Li et al., 2019). Similarly, the dramatic morphological differences observed in the spike axillary meristems at the PDR and TS stages between these two genotypes, correlate with the higher number of DEGs (Figure 1).

The large morphological differences at the later stages of spike development are reflected in the DEGs in Clusters 4, 5, 9, and 10. Genes in Cluster 4 are enriched in genes involved in the regulation of the early stages of spikelet development and include several known regulators of axillary branch and SM development and determinacy (Supplemental Table S3). Cluster 5 DEGs include multiple class-B, class-C, and class-E (except *SEP1-6* *=* *OsMADS34*) MADS-box floral genes that are upregulated in *vrn1* at TS, reflecting the progression of the axillary meristems into spikelets and florets in the WT but not in *vrn1 ful2* (Figure 1F). In contrast, transcript levels of the florigen antagonists *CEN2*, *CEN4*, and *CEN5* are strongly upregulated in the *vrn1 ful2* mutant in Clusters 9 and 10 (Figure 1G), which are also enriched in genes with photosynthetic functions reflecting the leaf-like structures generated by the spike axillary meristems in this mutant (Figure 1E).

Genes in Cluster 8 peak at the DR stage and include several genes previously shown to control the number of spikelets per inflorescence (Supplemental Table S3) and *SEP1-6*, the only wheat gene from the *LOFSEP*-clade that is upregulated in the *vrn1 ful2* mutant (Figure 1G). Finally, cluster 7 includes the three *SVP*-clade genes investigated in this study (Figure 1G), and genes known to either extend the activity of the IM or delay the transition of IM to SM identity (Supplemental Table S3). In summary, the complete list of DEGs presented in Supplemental Data Set S1 represents a valuable genomics resource for researchers interested in genes and gene networks that act downstream of *VRN1* and *FUL2* and play important roles in the early stages of spike and spikelet development. Supplemental Table S3 highlights a subset of these genes, which have already been found to play important roles in inflorescence and floret development in grasses.

### Localization of *SVP* and *SQUAMOSA* genes in wheat inflorescences

Our *in situ* experiments detected similar hybridization profiles in *SVP* and *SQUAMOSA* genes at the early stages of spike development. However, at PDR and TS stages expression of *VRT2* and *SVP1* was no longer detected in the IM or the early differentiating SMs (Figure 5, C and G), whereas expression of *VRN1* and* FUL2* persisted in these tissues (Supplemental Figure S5). Similar profiles have been reported for *SQUAMOSA* genes in previous studies in wheat and barley (*Hordeum vulgare*) (Preston and Kellogg, 2007, 2008; Alonso-Peral et al., 2011). The overlapping expression domains of *SVP* and* SQUAMOSA* genes during early stages of inflorescence development are consistent with the positive synergistic interaction between genes from these families observed in this study.

We detected ectopic expression of *VRT2* and* SVP1* within the developing spike axillary organs in the *vrn1 ful2* mutant but not in WT Kronos, confirming that *SQUAMOSA* genes are required to repress *SVP* gene expression at later stages of spike development in the WT. Ectopic expression of *VRT2* in glumes and lemmas was also detected by *in situ* hybridization in developing spikes of *Triticum* *turgidum* subsp. *polonicum*, which carries a *VRT-A2* allele with a shorter first intron (Liu et al., 2021a). Liu et al. (2021a) proposed that the structural changes in the first intron may disrupt the binding of the protein encoded by *MULTIFLORET SPIKELET 1* *(MFS1*, *TraesCS1A02G314200*, an *APETALA2/ETHYLENE-RESPONSIVE FACTOR*), resulting in ectopic expression of *VRT-A2* and elongated glumes. Interestingly, *MFS1* was downregulated in the apices of the *vrn1 ful2* mutant at PDR and TS stages (Cluster 4), which may provide a molecular link to the ectopic expression of *SVP* genes in this mutant.

### Wheat genes from the *SVP* clade accelerate heading time

In Arabidopsis, *SVP* acts as a flowering repressor (Hartmann et al., 2000) but the related *AGL24* acts as a flowering promoter (Michaels et al., 2003), suggesting a great degree of flexibility of genes from this clade to regulate flowering time. In pepper (*Capsicum annuum* L.) and tomato (*Solanum lycopersicum* L.), the *SVP* homologs *CaJOINTLESS* and* JOINTLESS* both function as flowering promoters (Cohen et al., 2012; Thouet et al., 2012), similar to the *VRT2* and *SVP1* genes in wheat. The late heading *vrt2 svp1* mutant showed a significant increase in leaf number, suggesting a delayed transition of the SAM from the vegetative to the reproductive stage. This delay was associated with a significant decrease in the transcript levels of flowering promoter genes *VRN1* and *FT1* and a significant increase of the flowering repressor *VRN2* in the leaves of the *vrt2 svp1* mutant (Supplemental Figure S4)*.* These three genes are part of a positive feedback loop that promotes wheat flowering by increasing the transcript levels of *FT1* (Distelfeld et al., 2009a). In Arabidopsis and rice, it was demonstrated that *FT1* homologs encode a mobile protein that is transported from the leaves to the SAM (Corbesier et al., 2007; Tanaka et al., 2015).

Changes in *VRT2* expression levels or in its spatiotemporal expression profiles can revert the function of this gene from a flowering promoter to a flowering repressor. The weak *UBI_pro_:VRT2* T#8 transgene accelerated flowering 1.3 days (Supplemental Figure S8A), whereas the strong T#2 event delayed heading up to 10 days (Figure 6A). A similar delay was observed when the barley *BM1* (∼*SVP3*) gene was constitutively expressed under the *ZmUBIQUITIN* promoter (Trevaskis et al., 2007). The effect of the constitutive expression of *VRT2* on heading time is likely modulated by genetic background and environment, since constitutive expression of *UBI_pro_:VRT2* in winter wheat accelerated flowering in unvernalized plants but not in fully vernalized plants (Xie et al., 2019).

One possible interpretation of the contrasting roles of *VRT2* on heading time in mutants and transgenic plants is that changes in VRT2 protein abundance and distribution in the transgenic plants may affect the composition and stability of different MADS-box protein complexes resulting in multiple pleiotropic effects. The altered balance of these multiple effects across a complex and interconnected regulatory network can lead to different outcomes depending on the timing, location and levels of *VRT2* expression. This hypothesis is based on interactions of VRT2 with all SQUAMOSA proteins (Figure 11), its ability to compete with other MADS-box proteins for interactions with SQUAMOSA proteins (Figure 12) and its regulatory effects on the expression of multiple floral genes (Figure 8). A similar hypothesis may explain the opposite effects of *VRT2* on plant height and SNS in the *vrt2* mutant and the strongest transgenic *UBI_pro_:VRT2* line.

### *SVP* and* SQUAMOSA* genes contribute to stem elongation

Both *SQUAMOSA* and *SVP* genes contribute to stem elongation in wheat. Mutants for all three *SQUAMOSA* genes have shorter stems, with the *vrn1 ful2 ful3* mutant being shorter than any other mutant combination (Li et al., 2019). Significant reductions in plant height have also been reported for the mutants of the *SQUAMOSA* orthologs in rice (*osmads14* and *osmads15*), suggesting a conserved function in grasses (Wu et al., 2017). Reduced plant height was also observed in the *vrt2* and *svp1* mutants in tetraploid wheat (Figure 3F) and in transgenic rice plants with reduced transcript levels of *OsMADS55* and *OsMADS47* (Lee et al., 2008), the rice orthologs of wheat *VRT2* and *SVP3*.

In contrast, constitutive expression of *SVP* genes *BM1* in barley (∼*SVP3*) (Trevaskis et al., 2007) and *OsMADS55* in rice (Lee et al., 2008) has been shown to promote stem elongation. We also observed a significant increase in stem length associated with the weak *UBI_pro_:VRT2* transgenic T#8 (Figure 6B). Increased expression of *VRT-A2* in the natural mutant *T. turgidum* subsp. *polonicum* was also associated with increased stem elongation (Adamski et al., 2021; Liu et al., 2021a). Because both *SVP* and* SQUAMOSA* genes promote stem elongation, we speculate that interactions between them may explain the drastic reduction in stem elongation in the *vrt2 vrn1 ful2* mutant.

### Mutations in *SVP* and* SQUAMOSA* genes alter inflorescence architecture

An unexpected phenotype of the *vrt2 svp1* mutant was the development of axillary spikelets or spikes in the nodes of the elongating stem (Figure 4, E–J and N), which indicated that both *VRT2* and *SVP1* function redundantly as repressors of axillary meristems in the nodes of the elongating stem. Axillary spikes were reported before in wheat but the causal genes were not identified (Wang et al., 2016). The *vrt2 svp1* axillary spikes were located in the same position as the ears in a maize plant, or the axillary inflorescences or “paracladia” in species from the Bambusoideae, Panicoideae, and Andropogoneae subfamilies (Stapleton, 1997; Vegetti, 1999). Andropogoneae species can develop large axillary inflorescences or small ones consisting of one or few spikelets (Vegetti, 1999), a variation similar to the one we observed in the *vrt2 svp1* mutant (Figure 4, E–J). It would be interesting to investigate if *SVP* genes in other grasses can also regulate the development of axillary inflorescences.

*SVP* MADS-box genes also play critical roles in inflorescence development by regulating meristem transitions in both monocot and eudicot plants. In tomato and pepper *SVP* genes *JOINTLESS* and * CaJOINTLESS* play important roles in the regulation of inflorescence architecture and are required to maintain the inflorescence state by suppressing the sympodial vegetative program (Szymkowiak and Irish, 2006; Cohen et al., 2012). Without *JOINTLESS* function, after one or two flowers are formed from the initial tomato IM, subsequent growth from that apex is vegetative (Szymkowiak and Irish, 2006).

In wheat, *SVP* and* SQUAMOSA* genes showed opposite effects on the regulation of the number of florets per spikelet but overlapping effects on the regulation of SNS. The number of florets per spikelet increased in the *ful2 UBI_pro_:VRT2* plants relative to the individual mutant or transgenic plants (Figure 9) but decreased in *vrt2 Vrn1 ful2* relative to *Vrn1 ful2* (Figure 10, E–F and I). The single *ful2* mutant also produced a higher number of florets per spikelet than Kronos WT indicating that this gene negatively regulates the number of florets per spikelet, an effect that was not observed for *vrn1* or *ful3* (Li et al., 2019). In contrast, ectopic expression of *VRT2* prolonged the activity of the SM and promoted the production of additional FM. These results suggest that dynamic changes in the relative abundance of *SQUAMOSA* and* SVP* genes are critical for the normal progression of floret meristems within spikelets.

In contrast with their antagonistic roles in the regulation of the SM activity, *SQUAMOSA* and *SVP* genes showed synergistic roles in promoting the transition of the IM to a TS. Individual *SVP* mutants (Figure 3E) and *SQUAMOSA* mutants show a delayed IM transition resulting in significant increases in SNS relative to the WT (Li et al., 2019). A synergistic effect of the *SQUAMOSA* and *SVP*-clade mutants was also evident in *vrt2 Vrn1 ful2* relative to *Vrn1 ful2* (Supplemental Figure S11C) and in the wheat *vrt2 vrn1 ful2* mutant, in which spikes remained undeveloped and eventually died within the sheaths (Supplemental Figure S9H).

Interactions between *SQUAMOSA* and *SVP* homologs have been reported also in Arabidopsis. Although no obvious changes in inflorescence architecture were observed in the *svp agl24* double mutant (Gregis et al., 2006), inflorescences of the Arabidopsis *svp agl24 ap1* triple mutant failed to produce FMs and continuously produced IMs (Gregis et al., 2008). Another MADS-box mutant combination in Arabidopsis including *svp agl24 soc1 sep4*, resulted in inflorescences with a striking increase in branching that was not observed in other combinations of these mutants (Liu et al., 2013). These four genes redundantly regulate inflorescence branching in Arabidopsis by repressing the expression of *TERMINAL FLOWER-LIKE 1* (*TFL1*) in the emerging FMs, a mechanism that only occurs in the presence of AP1 activity and that seems to be conserved in rice (Liu et al., 2013).

Interactions between MADS-box and *TFL1*/*CEN* homologous genes also regulate inflorescence architecture in pea. WT pea (*Pisum sativum*) plants have compound inflorescences with lateral branches carrying multiple flowers, but in the pea *veg1* mutant the inflorescence lateral meristems produced vegetative shoots instead of secondary inflorescences bearing flowers (Berbel et al., 2012). The mutated gene in *veg1* is a homolog of Arabidopsis *AGL79*, which encodes a SQUAMOSA MADS-box protein more distantly related to the wheat VRN1/FUL2/FUL3 proteins than Arabidopsis AP1/CAL/FUL. The pea *CEN* homolog *DET*, which is expressed only in the IM in the WT, was also expressed in the lateral meristems in the *veg1* mutant. Interestingly, the *det veg1* double mutant was able to produce flowers, indicating a role of *DET* in the transformation of the flowering branches into vegetative shoots in the pea *veg1* mutant (Berbel et al., 2012).

Our results also point to an interaction between *SVP*, *SQUAMOSA*, and *CEN* genes in wheat. The *CEN* genes showed reduced expression levels in developing spikes of *vrt2 Vrn1 ful2* relative to *Vrn1 ful2 and vrt2 svp1* relative to WT (Supplemental Figure S12, A and B), which suggests that *VRT2* promotes *CEN* transcription and provides a molecular link to the *CEN* upregulation in the *vrn1 ful2* mutant (Figure 1G). This hypothesis is also supported by similar *in situ* profiles of *CEN2*, *VRT2*, and *SVP1*, both in WT Kronos and *vrn1 ful2* (Supplemental Figure S7). Similarly, Arabidopsis AGL24 and SOC1 were shown to bind to *TFL1* regulatory regions to induce its expression in the SAM (Azpeitia et al., 2021). We hypothesize that the ectopic expression of *CEN* genes in the developing axillary organs in the spikes of *vrn1 ful2* may have contributed to their vegetative characteristics. We also hypothesize that their reduced expression in the spikelets of *vrt2 Vrn1 ful2* relative to *Vrn1 ful2* contributed to the reduced vegetative characteristic and floret number (Figure 10).

The effect of *CEN2* on inflorescence architecture is supported by the positive correlation reported between *CEN-D2* transcript levels and both SNS and floret number in *UBI_pro_:CEN-D2* transgenic wheat plants (Wang et al., 2017), and by the effect of the *cen-H2* mutants on SNS in barley (Bi et al., 2019). A mechanism for the regulation of inflorescence architecture involving competition between FT-like (florigen) and CEN/TFL1 (anti-florigen) proteins has been described in Arabidopsis and rice (Kaneko-Suzuki et al., 2018; Zhu et al., 2020). In rice, CEN/TFL1 proteins compete with FT-like florigen proteins for binding to 14-3-3 proteins in the formation of floral activation complexes that regulate *SQUAMOSA* genes and inflorescence development (Kaneko-Suzuki et al., 2018). Because *vrn1 ful2* mutants have both lower *FT1* expression in leaves and higher *CENs* expression in developing spikes (Li et al., 2019), the balance between these two groups of proteins in the inflorescence is likely altered. Given the known interaction of wheat FT1–14-3-3C–FDL2 complex with the *VRN1* promoter (Li et al., 2015), a florigen/anti-florigen competition mechanism represents an interesting area for future research in wheat spike development.

### Ectopic expression of *SVP* genes results in glumes and florets with leafy characteristics

Despite large changes in inflorescence architecture, the spikelets and florets of the wheat *vrt2 svp1* mutant looked normal. Similarly, the early flowering *svp agl24* double mutant in Arabidopsis showed only mild floral defects including reduced number of organs and partial homeotic transformation in the first whorl (Gregis et al., 2006). In contrast, ectopic expression of *VRT2* in tetraploid transgenic lines (Figures 6, E and F and 7) or in natural mutants such as *T. turgidum* subsp. *polonicum* (Adamski et al., 2021; Liu et al., 2021a) results in glumes and florets with vegetative characteristics. Ectopic expression of *SVP* genes has been associated with vegetative characteristic also in spikelet organs in barley (Trevaskis et al., 2007), rice (Sentoku et al., 2005), and maize plants carrying the dominant *Tunicate1* (*ZMM19*, ∼*SVP1*) pod corn mutation (Han et al., 2012; Wingen et al., 2012).

Ectopic expression of *ZMM19*, *OsMADS22*, and *OsMADS47* (∼*SVP2*) in Arabidopsis leads to leaf-like sepals and evergreen flowers similar to those observed with *35S_pro_:SVP* and* 35S_pro_:AGL24*, suggesting a conserved function (He et al., 2004; Fornara et al., 2008). Increases in sepal size have been also observed in transgenic tomato plants with reduced expression of the *SQUAMOSA* gene *LeMADS-*MC (Vrebalov et al., 2002) or with mutations in the *SEP* gene *Ej2* (Soyk et al., 2017). These results suggest that leaf-like sepals in eudicots can be induced by ectopic expression of *SVP*-clade genes or by mutations in *SQUAMOSA*- or *SEP-*clade genes. This result parallels the leaf-like glumes observed in wheat plants transformed with *UBI_pro_:VRT2* and in the *vrn1 ful2* mutant. We also observed strong interactions between *SQUAMOSA* and *SVP* genes on spikelet development in plants combining *ful2* and* UBI_pro_:VRT2* alleles (Figure 9; Supplemental Data Set S2).

We currently do not know whether the *SVP-*clade genes actively induce vegetative characteristics or if they have an effect on the repression of floral organs that leads to the regression to a “default” vegetative developmental program. We show in this study that constitutive expression of *VRT2* results in the downregulation of MADS-box A-, B-, C-, and most E-class genes, a function conserved in Arabidopsis (Gregis et al., 2009; Liu et al., 2009). Similar results were observed in the apices of wheat lines with the *VRT-2A* allele from *T. turgidum* subsp. *polonicum* (Liu et al., 2021a). Moreover, we showed that higher expression levels of *SEP1-2* in *vrt2 Vrn1 ful2* relative to *Vrn1 ful2*, was associated with more normal glumes and lemmas and a reduced proportion of spikelets with branches (Supplemental Figure S11). A role of the *SEP* genes in the development of normal glumes and lemmas was also demonstrated in the leaf-like lemmas and paleas observed in the rice triple mutant *osmads1 osmads5 osmads34* (Wu et al., 2018) Because continuous expression of *VRT2* can compete with the formation of SQUAMOSA–LOFSEP complexes (Figure 12), we speculate that downregulation of *SEP* genes or a reduction in their activity by protein competition may contribute to the observed vegetative characteristics in *UBI_pro_:VRT2* and *Vrn1 ful2* plants.

### Dynamic changes in expression of *SVP*, *SQUAMOSA*, and *SEP* genes are important for normal wheat spike and floral development

**Figure 13:**
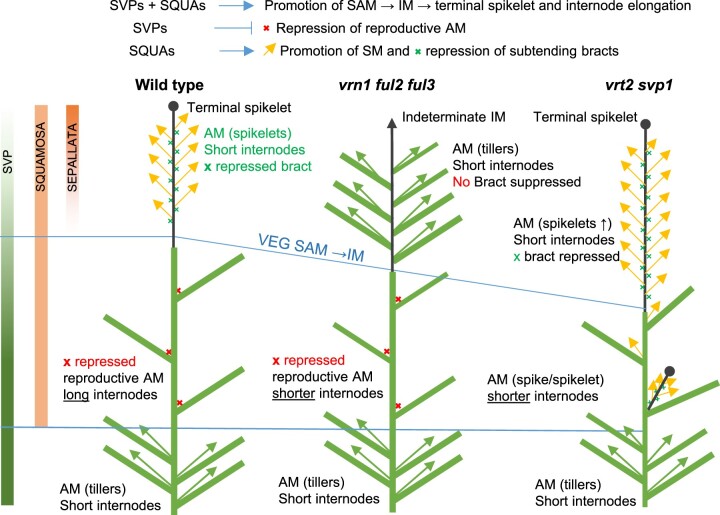
Working model of the role of *SVP* (*VRT2* and *SVP1*), *SQUAMOSA* (*VRN1*, *FUL2*, and *FUL3*) and *SEP* genes on the regulation of wheat plant architecture. Bars on the left represent transcript levels of genes from the three MADS-box clades during wheat development. The three plant models represent the architecture of WT, *vrn1 ful2 ful3* triple, and *vrt2 svp1* double mutants. Green rectangles represent leaves and sheaths, black lines the spike rachis (circle end = determinate and arrow end = indeterminate). The green Xs represent repressed bracts in the spike and the red Xs repressed buds in the elongating nodes. The lower part of the plants represents vegetative growth (*SVP* genes only), the region between the two blue lines the elongation zone (central region, *SVP* + *SQUAMOSA* genes), and the region above the blue lines the developing spikes (*SQUAMOSA* + *SEP* genes). In *vrn1 ful2 ful3*, the lateral SMs regress to VMs, and the bracts are not suppressed. In *vrt2 svp1*, the axillary buds in the elongation zone are no longer repressed and develop into axillary spikes or spikelets.

Based on the results from this and a previous study (Li et al., 2019), we propose the following working model for the roles of *SQUAMOSA* and * SVP* genes in the regulation of wheat reproductive development (Figure 13). Initially, *VRT2* and *SVP1* contribute to the acceleration of the transition of the VM to an IM, which in wheat is driven mainly by the induction of *VRN1* (Yan et al., 2003; Loukoianov et al., 2005)*.* Then, genes from the *SQUAMOSA*- and *SVP*-clades share a common role in inducing stem elongation and accelerating the transition of the IM to TS. Because we did not detect expression of *SVP-*clade genes in the IM at PDR and TS stages we speculate that this might be an indirect interaction. *VRT2* and *SVP1* also repress axillary meristems at the nodes of the elongating stem below the spike contributing to the formation of a single terminal inflorescence.

In the developing wheat spike, the downregulation of the *SVP* genes promoted by *SQUAMOSA* genes at the PDR and subsequent stages is critical for SM specification and normal spikelet development. A similar mechanism has been reported in Arabidopsis (Yu et al., 2004; Liu et al., 2007), in which chromatin immunoprecipitation (ChIP) experiments demonstrated that AP1 and SEP3 act as direct repressors of *AGL24* and* SVP* (Gregis et al., 2008).

MADS-box proteins can form different complexes as the abundance of different MADS-box proteins changes through development (Theissen et al., 2016). Our Y3H results suggest that the failure to downregulate the *SVP* genes in the *vrn1 ful2* mutant may result in competition of the SVP proteins with the formation of LOFSEP–SQUAMOSA protein complexes required for normal spikelet and floret development. This potential competition is avoided in the WT by the timely downregulation of the *SVP* genes*.* Through interactions with both the SVP and SEP proteins, the SQUAMOSA proteins play a pivotal role in the sequential transition between MADS-box protein complexes favoring early reproductive development and those favoring the development of floral organs. A similar function as protein interaction hubs between the flower induction pathway (e.g. SVP, AGL24, and SOC1) and floral organ identity proteins has been proposed in Arabidopsis for the SQUAMOSA proteins AP1 and FUL (de Folter et al., 2005).

In summary, this study shows that *SVP* and* SQUAMOSA* genes have synergistic effects on the acceleration of the transitions of the apical meristems (SAM to IM to TS) and stem elongation, but antagonistic effects on the regulation of axillary meristems in spikes and spikelets, with *SQUAMOSA* genes promoting the transition to floral organs and *SVP* genes having a regressive effect. Our results also show that it is possible to rationally manipulate the dosage or activity of these MADS complexes to optimize wheat spike architecture. Although mutations in both *SQUAMOSA* and *SVP* genes result in increases in SNS, the reduced pleiotropic effects of the *SVP* genes can facilitate their deployment in practical breeding applications. Our results for the separate *vrt2* and* svp1* mutants, and their individual homeologs, show that these effects can be readily fine-tuned in a polyploid species like wheat.

## Materials and methods

### Quant-Seq of *vrn1* and *vrn1 ful2* developing spikes

We collected SAMs from *vrn1* and* vrn1 ful2* mutants at four developmental stages: VEG, DR, PDR, and TS (Figure 1A). These four stages correspond to W1, W2.5, W3.25, and W3.5 stages in the Waddington Scale of wheat spike development (Waddington et al., 1983). We performed Quant-Seq analysis using four biological replicates for each of the four developmental stages, with each replicate including pools of 6 apices for PDR and TS stages, 9 apices for DR, and 12 apices for VEG apices. Sequencing of the 32 samples (2 genotypes × 4 developmental stages × 4 biological replicates) using Hi-seq (100-bp reads not paired) yielded an average of 7,335,215 unique reads per sample after filtering for duplicates, with an average read length of 74.3 bp (after trimming) and an average quality of 36.4 (Supplemental Table S2). BioProject numbers are available in the “Materials and methods” section “Accession Numbers”. Plant growth conditions are described in the section entitled “Growth conditions and phenotyping.”

We processed the raw reads using DOE JGI BBTools (https://sourceforge.net/projects/bbmap/) program bbduk.sh to remove Illumina adapter contamination and low-quality reads (forcetrimleft = 21 qtrim = r trimq = 10). Processed reads were mapped to the International Wheat Genome Sequencing Consortium (IWGSC) RefSeq version 1.0 genome assembly, using the STAR aligner (Dobin et al., 2013). We used parameters –outSAMtype BAM SortedByCoordinate –outSAMunmapped Within –outSAMattributes Standard –quantMode TranscriptomeSAM GeneCounts to generate Binary Sequence Alignment/Map (BAM) files for each sample. We used the high confidence gene models from IWGSC Refseq version1.0 (IWGSC_v1.1_HC_20170706.gff) in combination with the BAM files in the R program (featureCounts.R) which uses the Rsubread package (Liao et al., 2019) to calculate the overlap between reads and features. We used the “readExtension5” option that allows a read to be counted as belonging to a gene when the gene was a defined number of bases 5′ of the read (we used 500 bp).

The raw *t* test values between read counts of *vrn1* and *vrn1 ful2* were corrected for false discovery rate (FDR) using the R function p.adjust (method = “BH,” aka “FDR”) (Benjamini and Hochberg, 1995; R Core Team, 2020). DEGs between *vrn1* and* vrn1 ful2* mutants for each stage were defined as those with a fold change in transcript levels ≥2 and FDR ≤ 0.05. We then generated a list of nonredundant downregulated and upregulated DEGs across the four stages and performed a cluster analysis based on their expression profiles using the MultiExperiment Viewer software (www.tm4.org). For this analysis, the expression levels of each DEG were normalized to the average expression value of each DEG across genotypes and stages (mean normalized expression), and then clustered using K-means with a minimum limit of 10% of total genes per cluster. We then used the clustered lists as queries for BLAST analyses against a rice gene database available from Phytozome (Osativa_323_V7.0.cds_primaryTranscriptOnly.fa) to obtain a functional annotation for the DEGs (Supplemental Data Set S1). Finally, the lists containing the best rice blast hits were used to perform GO enrichment analysis using AgriGO web tool (http://bioinfo.cau.edu.cn/agriGO/).

### Identification of loss-of-function mutations in *VRT2*

The sequenced ethyl methanesulfonate mutagenized populations of the tetraploid wheat variety Kronos and hexaploid variety Cadenza (Krasileva et al., 2017) were screened for mutations using BLASTN with the sequences of *VRT2* (*TraesCS7A02G175200* and *TraesCS7B02G080300*) and *SVP1* (*TraesCS6A02G313800* and *TraesCS6B02G343900*) as queries. For *VRT-A2*, we detected 46 mutations that generated amino acid changes but we found no truncation mutations in the Kronos mutant population. Therefore, we screened the mutant population of the hexaploid wheat Cadenza, where we identified a mutation that generated a Q125* premature stop codon (mutant line Ca0424), which is predicted to eliminate 47% of the VRT2 protein, including part of the K-box and C terminal domains (Figure 2A, above gene model).

The Kronos mutant line K3404 carries a mutation in the donor splice site of the fourth intron of the *VRT-B2* gene, designated hereafter as *vrt-B2* (Figure 2a, below gene model). Sequencing of the RT-PCR products from K3404 revealed three *vrt-B2* alternatively spliced forms, all resulting in severe truncations (Supplemental Figure S2). The first alternatively spliced form showed a 5-bp insertion as a result of the utilization of the next available GT splicing site in intron 4. This resulted in a reading frame shift and a premature stop codon that is predicted to eliminate half of the protein including 30% of the conserved K domain. The second alternatively spliced form had an insertion of the last 4 bp of intron four between exons four and five that generated a reading frame shift and an early stop codon. Similar to the first alternative splice form, this change is also predicted to eliminate half of the protein and 30% of the K domain. Finally, the third alternatively spliced form was missing exons 3, 4, and 5. Although exons 6, 7, and 8 retained the correct reading frame, the deletion resulted in the elimination of 80% of the K domain (Supplemental Figure S2).

Because Kronos × Cadenza crosses result in hybrid necrosis, we crossed Ca0424 to an F_2_ plant from the cross between the hexaploid Insignia and the tetraploid Kronos as a bridge cross. We then intercrossed the F_1_ with the *vrt-B2* mutant K3404 to combine both mutations (Figure 2C). To reduce the background mutations, we backcrossed the F_1_ plant from the cross between *vrt-A2* and *vrt-B2* 3 times to Kronos, and from the segregating BC_2_F_2_ plants we selected a double homozygous mutant *vrt-A2 vrt-B2*, which was designated as *vrt2*.

To test the genetic interaction between *vrt2* and members of the MADS-box genes from the *SQUAMOSA*-clade (*VRN1* and *FUL2*), we combined *vrt2* with loss-of-function mutations at *vrn1 ful2* in the same Kronos background (Li et al., 2019). Because the *vrn1 ful2* line is sterile, we used a line heterozygous for *VRN-A1* and *FUL-B2* for crossing*.* In the progeny, we selected two pairs of isogenic lines, one with no functional copies of *VRN1* (*vrt2 vrn1 ful2/vrn1 ful2*) and one heterozygous for *Vrn-A1* and homozygous for all the other truncation mutations (*vrt2 Vrn1/ful2 Vrn1 ful2*)*.* All these mutant lines were developed in a Kronos background with no functional copies of *VRN2* to avoid the extremely late heading of the *vrn1* mutant in the presence of *VRN2*, which is a strong flowering repressor in wheat (Distelfeld et al., 2009b). We self-pollinated the F_1_ plant and from the F_2_ plants we selected lines homozygous for *vrn1 ful2* and either homozygous for *vrt2* or for the WT alleles (Figure 2C). Because the *vrt2 vrn1 ful2* mutant failed to form spikelets, we also selected lines *Vrn1 ful2* with and without *vrt2* to study its effect on spike morphology.

### Identification of loss-of-function mutations in *SVP1*

For *SVP-A1*, we identified the Kronos line K4488 that carries a mutation in the splice donor site in the third intron, designated as *svp-A1* (Figure 2B). The sequencing of *SVP-A1* RT-PCR products from K4488 revealed two alternatively spliced forms (Supplemental Figure S2). The first one lacks the third exon, which alters the reading frame and generates a premature stop codon that eliminates >60% of the SVP-A1 protein. Because this deletion includes the complete K domain, the resulting protein is likely not functional. The second alternatively spliced form lacks both the second and third exons, which results in the loss of 47 amino acids but does not alter the reading frame. Since this predicted deletion includes the end of the MADS domain and the beginning of the K domain, the resulting protein is likely not functional.

For *SVP-B1*, we identified Kronos line K0679 with a mutation that generates a premature stop codon in the third exon (Q99*), designated as *svp-B1* (Figure 2B). We crossed both mutants separately 2 times to the parental Kronos to reduce background mutations (BC_1_) and then combined them by crossing and selection in BC_1_F_2_, to generate the double mutant designated *svp1*. Finally, we intercrossed *svp1* and* vrt2*, self-pollinated the F_1_, and selected F_2_ plants homozygous for the four mutations (*vrt-A2 vrt-B2 svp-A1 svp-B1*) which were designated as *vrt2 svp1* (Figure 2C).

### *In situ* hybridization

We performed *in situ* RNA hybridization following the protocol described previously (Zhong et al., 2021). Tissues were obtained from diploid *T. monococcum* (accession PI 167615), tetraploid Kronos WT, and Kronos *vrn1 ful2* mutant. We amplified DNA fragments of 300–400 bp covering the end of the coding region and the 3′–untranslated region (UTR) from *T. monococcum* with gene-specific primers appended with T7 or T3 promoter or from Kronos with gene-specific primers, and then inserted them into pGEM-T easy vectors (Promega, Madison, WI, USA). The probes were synthesized using T7 or T3 RNA Polymerase (Promega) and labeled with Digoxigenin-11-UTP (Roche). Images were taken using a Zeiss AxioImager M2 microscope with an AxioCam512 color camera., or a Zeiss SteREO Discovery.V20 microscope with an AxioCam506 color camera. Primers used to amplify the hybridization probes are described in Supplemental Table S4.

### SEM

Apices from the different genotypes and developmental stages were dissected and fixed for a minimum of 24 h in Formaldehyde Alcohol Acetic Acid (FAA) (50% ethanol, 5% (v/v) acetic acid, and 3.7% (v/v) formaldehyde), and then dehydrated through a graded ethanol series to absolute ethanol. Samples were critical point dried in liquid CO_2_ (tousimis 931 Series critical point drier), mounted on aluminum stubs, sputter-coated with gold (Bio-Rad SEM Coating System Model E5100), and examined with a ThermoFisher Quattro ESEM SEM operating at 5 kV. Images were recorded at high definition and saved as TIFF files.

### Transgenic plants and complementation

Transgenic Kronos plants overexpressing *VRT2* were generated at the UC Davis Plant Transformation Facility (http://ucdptf.ucdavis.edu/) using the Japan Tobacco vector pLC41 (hygromycin resistance) and transformation technology licensed to UC Davis. The coding region of *VRT-A^m^2* gene from *T.* *monococcum* accession G3116 (GenBank MW218446) was cloned downstream of the maize *UBIQUITIN* promoter with a C-terminal 4×MYC tag (henceforth *UBI_pro_:VRT2*). *Agrobacterium* strain EHA105 was used to infect Kronos immature embryos and all transgenic plants were tested by PCR using primers described in Supplemental Table S4.

To test the complementation of the mutant phenotypes, we crossed *UBI_pro_:VRT2* plants with the *vrt2* mutant. We self-pollinated the F_1_ plants and used molecular markers to select F_2_ plants homozygous for the *vrt-A2* and *vrt-B2* mutations with and without the transgene. These sister lines were evaluated in growth chamber as described in the following section.

### Growth conditions and phenotyping

We grew the plants used for the Quant-Seq experiment and for phenotypic evaluation of mutants and transgenic plants in PGR15 CONVIRON growth chambers under long-day photoperiod (16-h light/8-h dark) and temperatures of 22°C during the day and 18°C during the night. The light intensity of the sodium halide lights was ∼330 μmol m^−2^ s^−1^. Plants were germinated in Petri dishes at 4°C for 3–5 days. After the first leaf emerged, we transplanted the seedlings into Sun Gro professional growing mix (Sunshine Mix #1) in one-gallon pots, and recorded days to heading from this day until emergency of half of the main spike from the flag leaf. Length measurements were taken at maturity for the complete plants and for each of the internodes and peduncle separately.

We also evaluated the *vrt2* mutant in a field experiment (sown November 22, 2019) at the UC Experimental Field Station in Davis, CA (38° 32′ N, 121° 46′ W). We used 1-m rows with 20 plants each as experimental units, organized in a completely randomized design. The experiment included 20 replications for *vrt2* and the WT sister lines, and 10 replications for *vrt-A2* and *vrt-B2.* Plants in the field were evaluated for heading time, SNS, and total plant height (measured from the soil to the top of the main spike excluding awns).

### Statistical analyses

Effects of individual homeologs (A and B genome) or of individual genes (e.g. *VRT2* and *SVP1*) and their interactions were compared using 2 × 2 factorial analysis of variance (ANOVA) with homeologs or genes as factors and alleles as levels. Simple effects were evaluated using orthogonal contrasts. Means of the individual genotypes were compared with the WT using Dunnett’s test. Homogeneity of variances was tested with the Levene’s test and normality of residuals with the Shapiro–Wilk test. When necessary, we transformed data to meet the assumptions of the ANOVA. All statistical analyses were performed using SAS version 9.4. Results of all statistical analyses are presented in Supplemental Data Set S2. The distribution of the data within each genotype is presented with box plots including individual data points generated with Excel. The middle line of the box represents the median and the x represents the mean. The bottom line of the box represents the first quartile and the top line the third quartile. The whiskers extend from the ends of the box to the minimum value and maximum value. A data point was considered an outlier if it exceeded a distance of 1.5 times the interquartile range. The number of plants analyzed is indicated in each graph.

### cDNA preparation and qRT-PCR analysis

To quantify transcript levels of different flowering genes in transgenic plants overexpressing *VRT2*, we extracted total RNA from pools of 6–8 SAMs at TS stage from four biological replicates using the Spectrum Plant Total RNA Kit (Sigma-Aldrich St Louis, MO, USA). Samples were collected 4–5 h after the lights were turned on in the morning. The cDNA was synthesized using the High-Capacity cDNA Reverse Transcription Kit (Thermo Fisher Scientific, 4368814) from 2 μg RNA treated with RQ1 RNase-free DNase (Promega). The cDNA was then diluted 20-fold in water and 5 μL of the dilution was used for the qRT-PCR analysis. The Quantitative PCR was performed using the 7500 Fast Real-Time PCR system (Applied Biosystems, Waltham, MA, USA) with 2×VeriQuest Fast SYBRGreen qPCRMaster Mix (Affymetrix, 75690). The relative transcript level was determined for each sample and normalized using *ACTIN* as an endogenous control. The normalization was performed as described previously (Livak and Schmittgen, 2001). Melting curve analyses at the end of the process and “no template controls” were performed to ensure product-specific amplification without primer-dimer artifacts. Primer sequences are given in Supplemental Table S4.

### Y2H assay and BiFC

We used the GAL4-based Y2H system to investigate protein interactions. We amplified the full-length cDNAs of the different genes from the *SQUAMOSA-* (*VRN1*, *FUL2*, and *FUL3*), *SVP-* (*VRT2*, *SVP1*, *SVP3*), and *SEP* clades (*SEP1-2*, *SEP1-4*, and *SEP1-6*) and cloned them into the gateway pDONR/Zeo Vector (Catalog number: 12535035) using the primers listed in Supplemental Table S4. We then cloned these genes into the Takara Bio Y2H vectors pGADT7 (activation-domain vector) and pGBKT7 (DNA-binding domain vector) by either restriction enzyme-based cloning or In-Fusion HD Cloning method (638910In-Fusion HD Cloning Plus Takara). Primer sequences used to generate Y2H and Y3H constructs are listed in Supplemental Table S4. Both bait and prey vectors were transformed into yeast AH109 Gold strain (*Saccharomyces cerevisiae* from TaKaRa/Clontech). The cotransformants were plated on selective solid Synthetic Dropout agar medium without leucine (L) and tryptophan (W) (SD-L-W). Positive transformants were re-plated on Synthetic Dropout medium lacking L, W, histidine (H), and adenine (A) to test for interaction (SD-L-W-H-A). We cotransformed each bait vector with pGADT7 and each prey vector with pGBKT7 to test for autoactivation.

For the BiFC (or split YFP) assays, we cloned the same genes into modified Gateway-compatible vectors UBI_pro_:NYFP-GW and UBI_pro_:CYFP-GW by recombination reactions (*UBI_pro_* = the maize *UBIQUITIN* promoter, 1,986-bp upstream of the ATG). These vectors generated fusion proteins with YFP-N-terminal fragment or YFP-C-terminal-fragment at the N-terminus and the proteins being tested at the C-terminus. Wheat protoplasts were prepared, transfected, and visualized as described in (Shan et al., 2014).

### Y3H assays

The pBridge Y3H system (Clontech CATALOG No. 630404) was used to test whether the wheat VRT2 protein can interfere with the interactions between SEP and SQUAMOSA proteins. This vector can express two proteins, a DNA-binding domain fusion, and a second protein (Bridge protein) that is controlled by pMET25, an inducible promoter responsive to methionine levels in the medium. The Bridge protein is only expressed in the absence of methionine and inhibited by the addition of 1 mM methionine. For each pBridge vector, one of the *LOFSEPs* (*SEP1-2, SEP1-4*, or *SEP1-6*) genes was fused to the DNA-binding domain, and the *VRT2* gene was inserted downstream of the MET25 promoter. The same prey vectors generated for *VRN1*, *FUL2*, and *FUL3* in Y2H assays were used in Y3H assays. Each pBridge vector was then paired with one prey vector and co-transformed into yeast Gold. Transformants containing both vectors were selected on SD-L-W medium. Protein interactions were quantified using quantitative α-galactosidase assays as described before (Li et al., 2011). All constructs used in Y2H and Y3H assays have the GAL4 DNA binding (bait) and activation domains (prey) at the N-terminus, and the proteins being tested at the C-terminus of the fusion protein.

### Accession numbers

The *T. monococcum VRT-A^m^2* sequence used for the constitutive expression construct is deposited in GenBank under accession number MW218446. The Quant-Seq datasets for the *vrn1* and* vrn1ful2* mutants have been deposited in GenBank under the following project numbers (each including four biological replicates): PRJNA681065 (*vrn1*, VEG samples), PRJNA681067 (*vrn1*, DR), PRJNA681097 (*vrn1*, post DR) PRJNA681099 (*vrn1*, TS), PRJNA681036 (*vrn1 ful2*, VEG samples), PRJNA680890 (*vrn1 ful2*. DR), PRJNA681027 (*vrn1 ful2*, post DR), and PRJNA681032 (*vrn1 ful2*, TS). Seed stocks have been deposited in the National Small Grain Collection for the following Kronos mutants: *vrn1 vrn2* (PI 698812), *ful2 vrn2* (PI 698814), *ful3 vrn2* (PI 698815), *vrt2* (PI 698811), and *svp1* (PI 698813). All other data and genetic materials are available from the authors upon request.

## Supplemental data

The following materials are available in the online version of this article.

**Supplemental Figure S1.** Phylogenetic relationship among SVP proteins in wheat, barley, rice, and Arabidopsis.

**Supplemental Figure S2.** Effects of *vrt2* and *svp1* mutations on the encoded proteins.

**Supplemental Figure S3.** Effects of mutations in the A and B genome homeologs of *VRT2* and *SVP1.*

**Supplemental Figure S4.** Transcript levels of flowering genes *FT1*, *VRN1*, and *VRN2* in the fifth leaf of single mutants *vrt2* and *svp1*, combined mutant *vrt2 svp1* and WT control.

**Supplemental Figure S5.**  *In situ* hybridizations of *VRN1* and *FUL2* in wheat spikes at different developmental stages.

**Supplemental Figure S6.**  *In situ* hybridizations of *VRT2* and *SVP1* in *T. monococcum* spikes at different developmental stages.

**Supplemental Figure S7.**  *In situ* hybridizations of *CEN2* expression in Kronos spikes at different developmental stages.

**Supplemental Figure S8.** Complementation of the *vrt2* mutation by the weak *UBI_pro_:VRT2* transgenic line T#8.

**Supplemental Figure S9.** Phenotypic comparison between *vrn1 ful2* and *vrt2 vrn1 ful2* mutants (in a *vrn2* mutant background).

**Supplemental Figure S10.** SEM images of developing spikes.

**Supplemental Figure S11.** Effect of the *vrt2* mutation in the partial mutant *Vrn1 ful2* (the underline indicates a plant heterozygous for *Vrn-A1 vrn-A1* and homozygous *vrn-B1 vrn-B1*).

**Supplemental Figure S12.** Effect of *VRT2* and *SVP1* on the transcript levels of *SEP*, *CEN*, and *TB1* genes in developing spikes.

**Supplemental Figure S13.** Auto-activation tests for the bait and prey vectors used in Y2H assays.

**Supplemental Figure S14.** Y2H interactions among proteins within the SQUAMOSA- and SVP-clades.

**Supplemental Figure S15.** Y2H interactions between wheat MADS-box proteins of the SQUAMOSA, SVP, and SEP classes.

**Supplemental Figure S16.** BiFC between proteins of the SQUAMOSA-clade and proteins of both the SVP and SEP clades in wheat protoplasts.

**Supplemental Table S1.** Nomenclature, accession numbers, and synonyms of wheat genes used in this study and their rice orthologs.

**Supplemental Table S2.** Summary statistics for the Quant-Seq samples.

**Supplemental Table S3.** DEGs between *vrn1* and *vrn1 ful2* at four different developmental stages of spike development.

**Supplemental Table S4.** Primers used for gene cloning, genotyping of mutations and transgenic plants, qRT-PCR, generation of Y2H and Y3H constructs, and *in situ* hybridization.

**Supplemental Table S5.** Effects of *vrt2* and *svp1* mutations on heading time, SNS, stem length, and leaf number in growth chambers under LD conditions.

**Supplemental Table S6.** Summary of Y2H interactions and BiFC interactions tested among the SQUAMOSA, SVP, and SEP MADS-box proteins.

**Supplemental Data  Set S1.** DEGs in Clusters 1–10.

**Supplemental Data  Set S2.** Statistical analysis tables.

**Supplemental File S1.** Sequence alignment used to produce the phylogenetic tree in Supplemental Figure S1.

## Supplementary Material

koab243_Supplementary_Data

## Notes

C.L., J.M.D., and J.D. designed the research. K.L. performed most of the experimental work. J.M.D., C.L., H.L., and C.Z. performed research, J.J. contributed the SEM images, M.V.K. and J.Z. contributed *in situ* hybridizations, C.L., H.L., J.M.D., K.L., and J.D. analyzed the data. C.L., J.M.D., K.L., H.L., and J.D. wrote the article.

The authors responsible for distribution of materials integral to the findings presented in this article in accordance with the policy described in the Instructions for Authors (https://academic.oup.com/plcell) are: Jorge Dubcovsky (jdubcovsky@ucdavis.edu) and Juan Manuel Debernardi (jmdebernardi@ucdavis.edu).
