# Supplementary material for: Interactions between SQUAMOSA and SHORT VEGETATIVE PHASE MADS-box proteins regulate meristem transitions during wheat spike development
Source: Plant Cell. 2021 Nov 2;33(12):3621–44. doi: 10.1093/plcell/koab243 (PMC8643710; doi:10.1093/plcell/koab243)
Supplement: koab243_Supplementary_Data [file koab243_supplementary_data.zip › Supplemental Data SNo.pdf]

SUPPLEMENTAL FIGURES

Supplemental Figure S1. Phylogenetic relationship among SVP proteins in wheat, barley, rice and Arabidopsis. (Supports Figure 1.)

We inferred the evolutionary history of MADS-box SVP proteins from wheat, barely, rice and Arabidopsis using the Neighbor-Joining method. We used the MUSCLE protein alignment presented in Supplemental File 1 to calculate the optimal tree with branch lengths proportional to the distances used to infer the phylogenetic tree. The evolutionary distances were computed using the Poisson correction method, and are in the units of the number of amino acid substitutions per site. We show the percentage of replicate trees in which the associated taxa clustered together in the bootstrap test (1000 replicates) next to the branches. We removed all ambiguous positions for each sequence pair (pairwise deletion option) and the final dataset included 236 positions. We conducted all the analyses using MEGA X (Kumar et al., 2018, Mol. Biol. Evol. 35:1547-1549). Arabidopsis SVP and AGL24 proteins were included as outgroups of the grass SVP proteins.

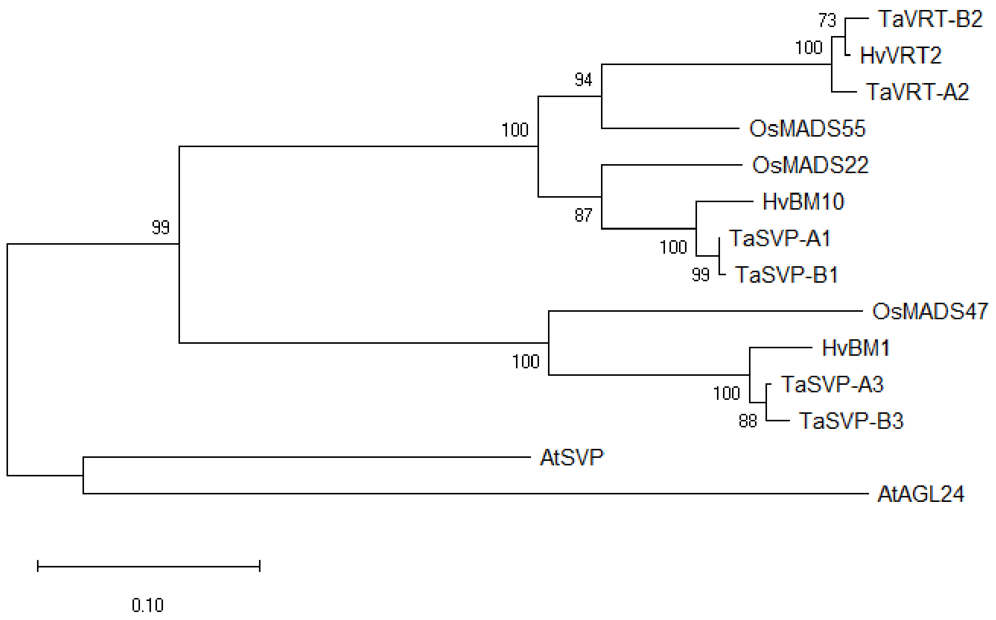

Below we present the tree in Newick machine-readable format:

(((((TaVRT-B2,HvVRT2),TaVRT-A2),OsMADS55),(OsMADS22,(HvBM10,(TaSVP-A1,TaSVP-B1)))),(OsMADS47,(HvBM1,(TaSVP-A3,TaSVP-B3))),(AtSVP,AtAGL24));

Supplemental Figure S2. Effects of *vrt2* and *svp1* mutations on the encoded proteins. (Supports Figure 2.)

Rectangles of different colors represent exons. Violet and green lines represent MADS and K box domains respectively. A violet triangle indicates the position where exons are missing. * = stop codon.

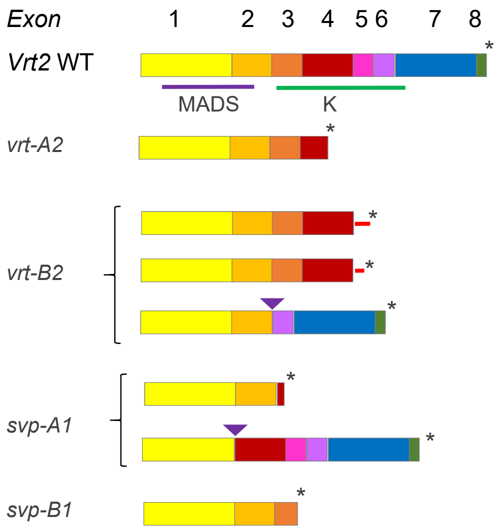

Supplemental Figure S3 Effects of mutations in the A and B genome homeologs of *VRT2* and *SVP1.* (Supports Figure 3.)

(A-F) Growth chamber experiments. The number of replications is indicated in the X axis. **(A, C, E)** Effect of *vrt2.* **(B, D, F)** Effect of *svp1.* **(A-B)** Heading time **(C-D)** Spikelet number per spike. **(E-F)** Stem length: each node is in a different color and peduncles are in green (-1 is the closest node to the peduncle and -4 the most basal node). **(G-I)** Field experiment for *vrt2* mutants only. **(G)** Spikelet number per spike. **(H-I)** Plant height. Number of replications are below the box plots. Significance values are based on Dunnett tests compared to the WT. ns = not significant, * = $P < 0.05$, ** = $P < 0.01$, *** = $P < 0.001$ (Supplemental Data Set 2). Box-plot features are explained in Statistical analyses section of Material and Methods.

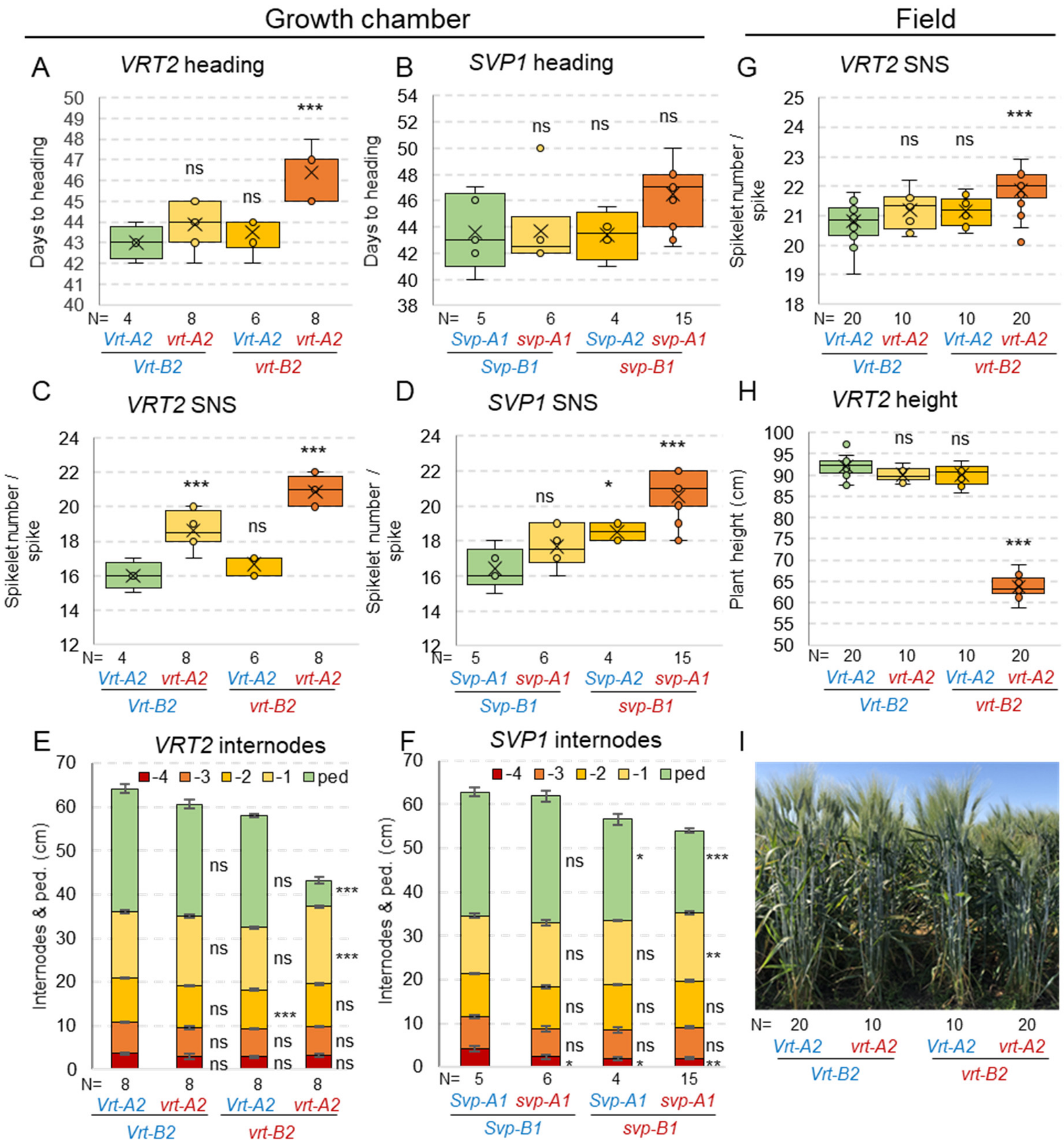

Supplemental Figure S4 Transcript levels of flowering genes *FT1*, *VRN1*, and *VRN2* in the fifth leaf of single mutants *vrt2* and *svp1*, combined mutant *vrt2 svp1* and WT control. (Supports Figure 3.)

(A) *FT1*. **(B)** *VRN1*. **(C)** *VRN2*. Transcript levels are expressed relative to *ACTIN* as endogenous control using the ∆Ct method. ns= not significant, * = $P < 0.05$, ** = $P < 0.01$, *** = $P < 0.001$, calculated using Dunnett tests versus the WT control (Supplemental Data Set 2). The number of replications is indicated in the X axis below each genotype. Box-plot features are explained in Statistical analyses section of Material and Methods.

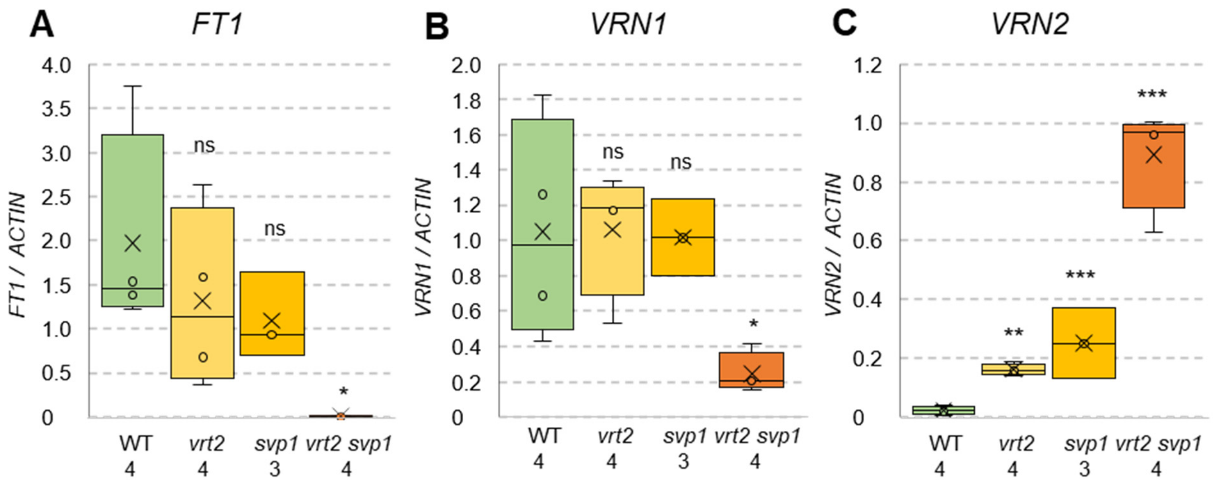

Supplemental Figure S5. *In situ* hybridizations of *VRN1* and *FUL2* in wheat spikes at different developmental stages. (Supports Figure 5.)

(A-E) *VRN1.* **(F-J)** *FUL2.* **(A-D** and **F-I)** Antisense probes. **(E** and **J)** Control sense probes in Kronos. **(A-C** and **F-H)** Kronos. **(D** and **I)** *T. monococcum.* **(A**, **F**, **E** and **J)** = early DR. **(B**, **D**, **G**, and **I)** = DR. **(C**, **H)** = TS. Probes are from *T. monococcum* and primers are described in Supplemental Table 4. Bars are 500 μm.

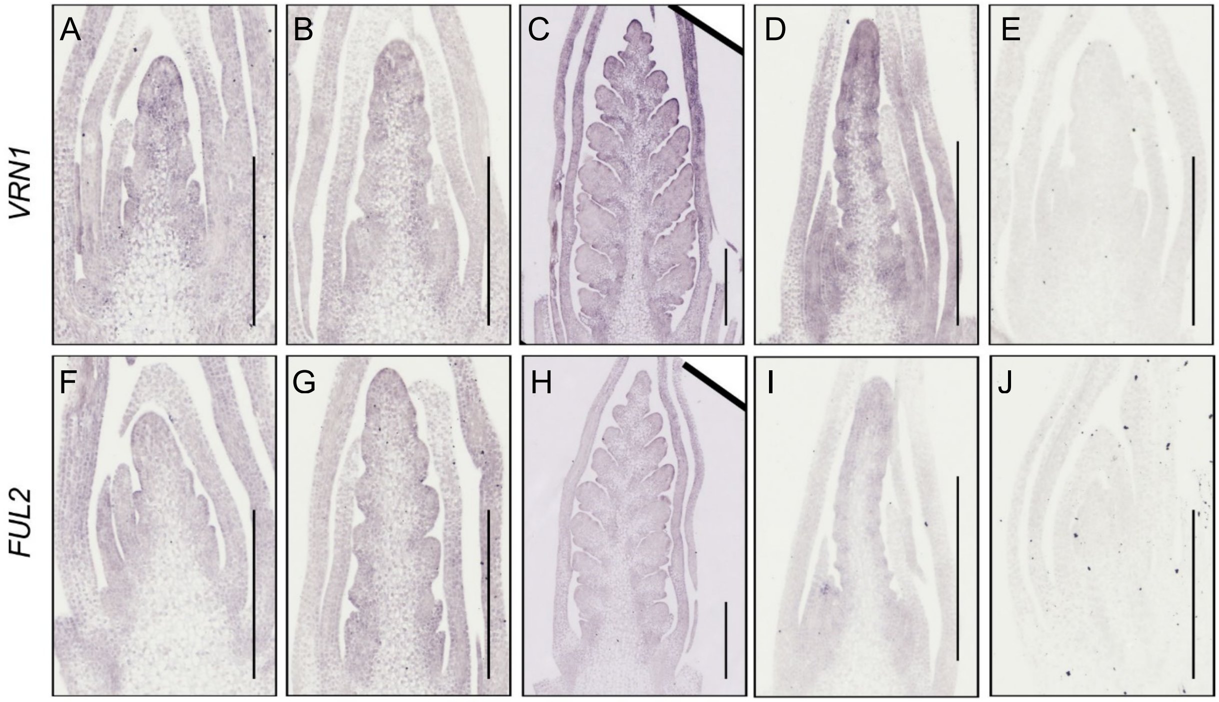

Supplemental Figure S6. *In situ* hybridizations of *VRT2* and *SVP1* in *T. monococcum* spikes at different developmental stages. (Supports Figure 5.)

(A-C) *VRT2.* **(D-F)** *SVP1.* **(A**, **B**, **D** and **E)** = early DR. **(F)** = DR. **(C)** = PDR. Probes are from *T. monococcum* and primers are described in Supplemental Table 4. Bars are 500 µm.

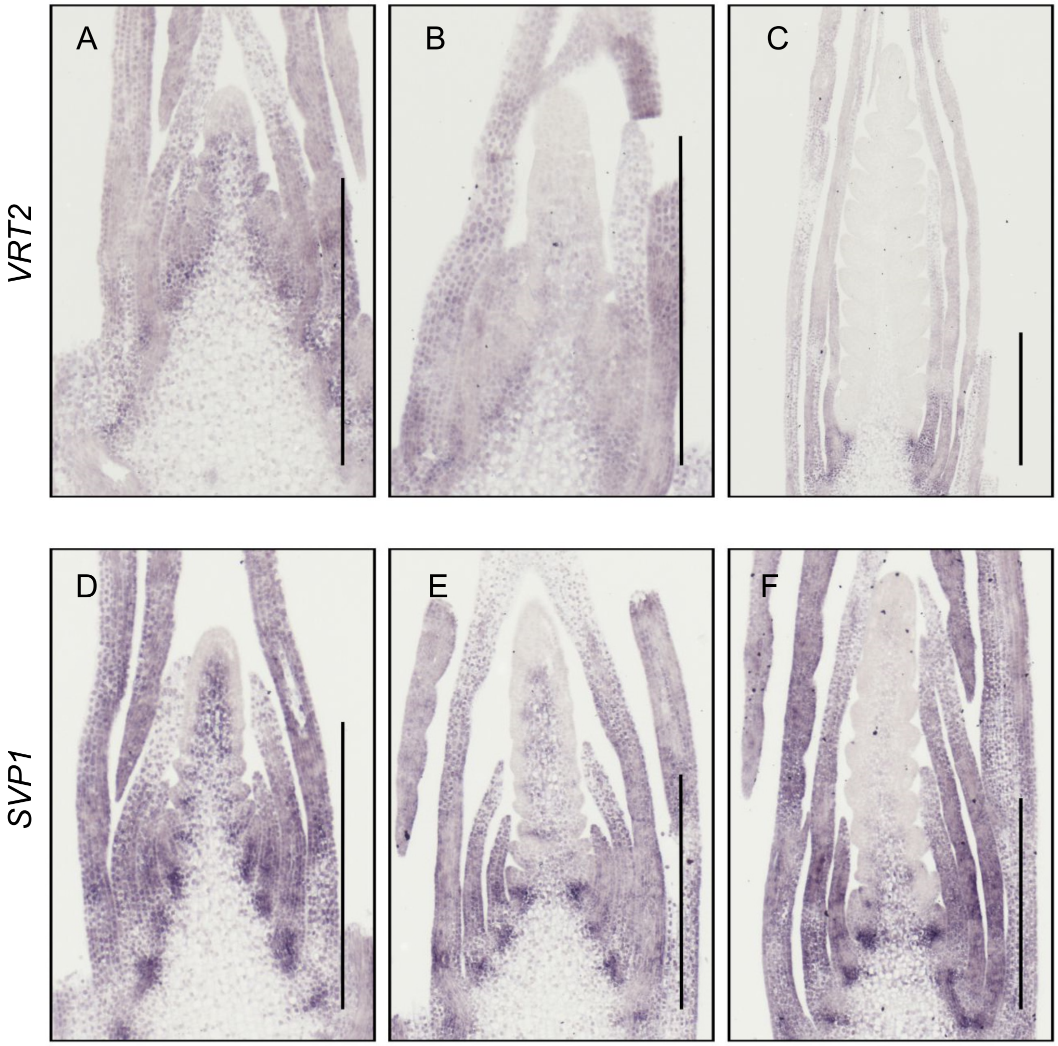

Supplemental Figure S7. *In situ* hybridizations of *CEN2* expression in Kronos spikes at different developmental stages. (Supports Figure 5.)

(A-E) Antisense probe. **(F)** Control sense probe. **(A-D)** Wild type Kronos. **(E)** Kronos *vrn1 ful2* mutant. Probes are from *T. monococcum* except for **(E)** which is from Kronos. **(A-C)** = early DR. **(F)** = DR. **(D)** = TS. **(E)** = equivalent to WT TS but without a terminal spikelet andwith lateral vegetative meristems. Primers for the probes are described in Supplemental Table 4. Bars are 500 μm.

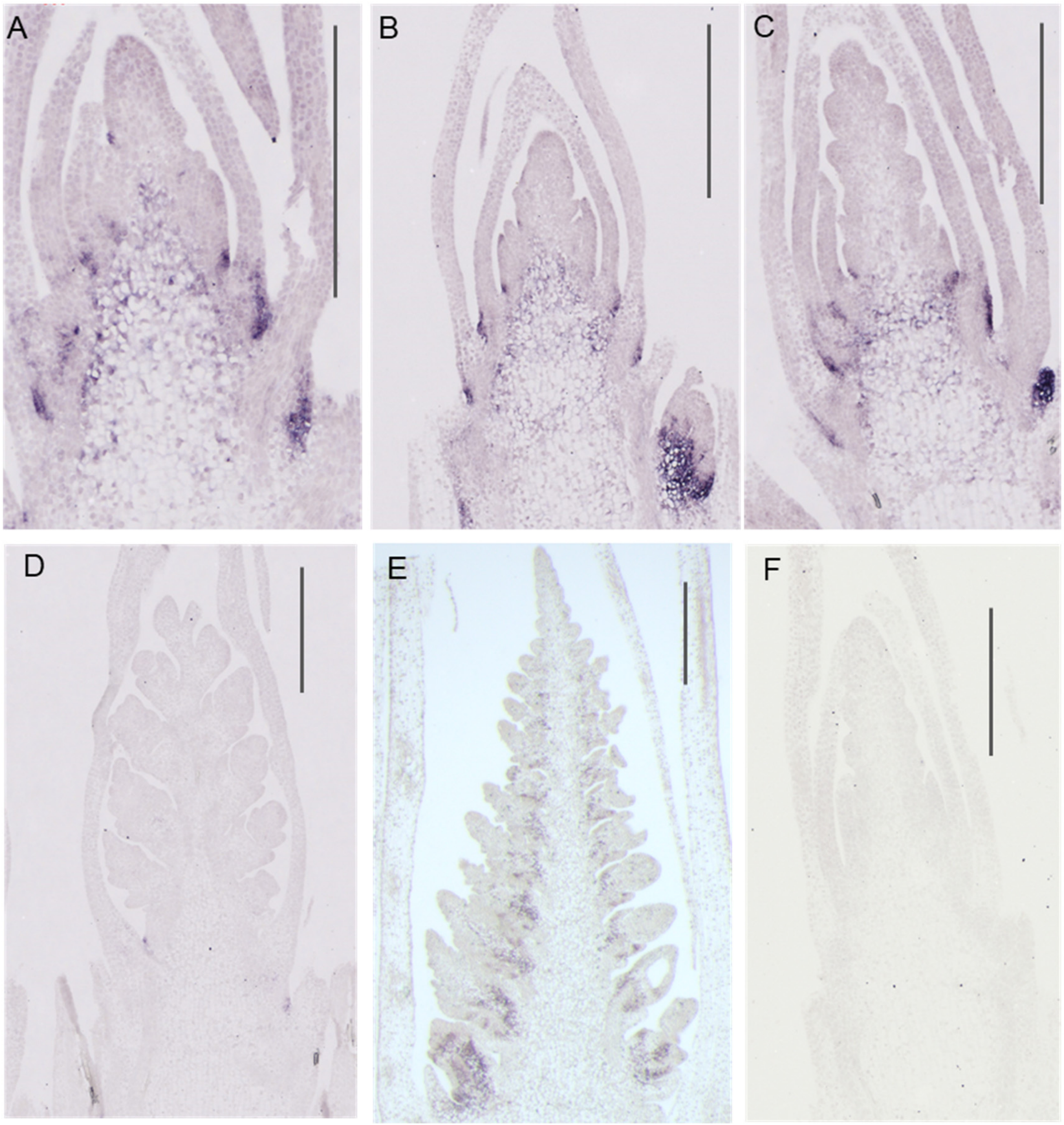

Supplemental Figure S8 Complementation of the *vrt2* mutation by the weak *UBI*$_{pro}$*:VRT2* transgenic line T#8. (Supports Figure 6.)

(A) Heading time. **(B)** Peduncle length. **(C)** Spikelet number per spike. WT = homozygous wild type *VRT2* alleles for both homeologs. *vrt2* = homozygous loss-of-function *vrt-A2* and *vrt-B2* alleles. T = *UBI*$_{pro}$*:VRT2* transgene present. NT = Transgene absent. N = 12 biological replicates per genotype (except WT NT where N = 10). Different letters indicate significant differences using a Tukey test $P < 0.05$ (Supplemental Data Set 2). Box-plot features are explained in the Statistical analyses section of Material and Methods.

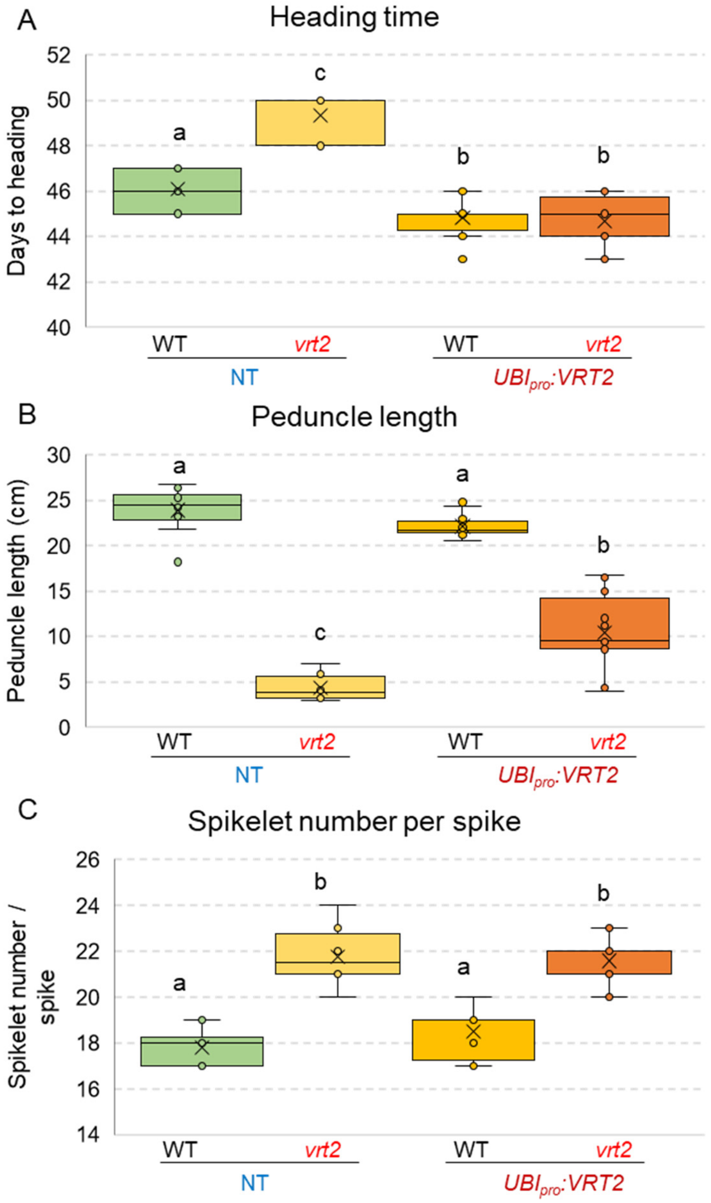

Supplemental Figure S9. Phenotypic comparison between *vrn1 ful2* and *vrt2 vrn1 ful2* mutants (in a *vrn2* mutant background). (Supports Figure 10.)

(A-B) Images of 110-days old plants of *vrn1 ful2* and *vrt2 vrn1 ful2*. **(A, C** and **G)** *vrn1 ful2* **(B, D-F** and **H)** *vrt2 vrn1 ful2*. **(C)** Spikes with axillary tillers emerged from most shoots in *vrn1 ful2.* **(D)** No spike emergence was observed in 84% of the *vrt2 vrn1 ful2* shoots. **(E-F)** Severely deformed **s**pikes with axillary tillers emerged from 16% of the shoots in *vrt2 vrn1 ful2* (detail in F). **(G-H)** Time course dissections of an emerging spike in *vrn1 ful2* **(G)** and a non-emerging spike in *vrt2 vrn1 ful2*, which eventually died inside the sheath **(H)**.

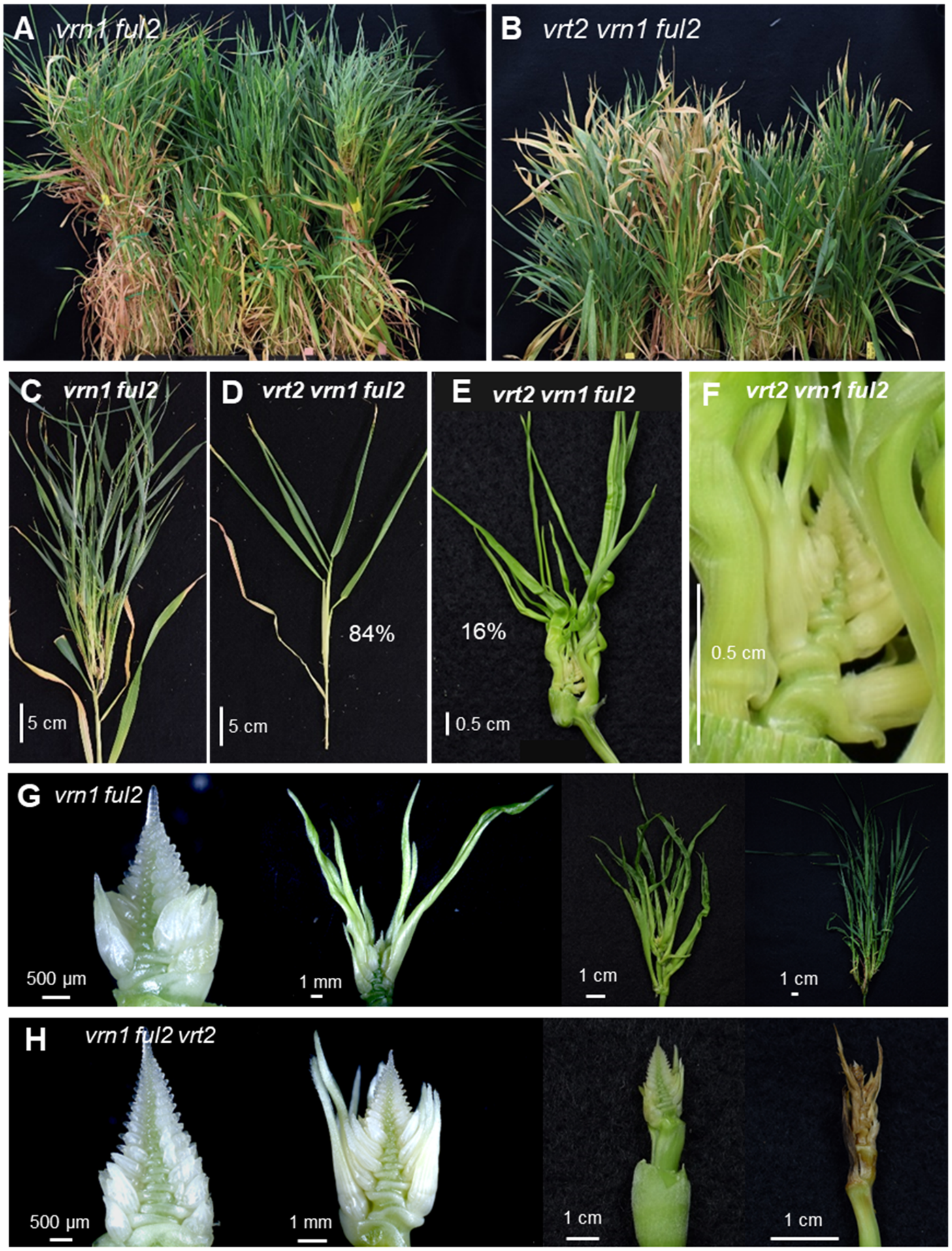

Supplemental Figure S10. Scanning Electron-Microscope (SEM) images of developing spikes. (Supports Figure 10.)

(A and **B)** *vrn1 ful2.* **(C** and **D)** *vrt2 vrn1 ful2.* **(E** and **F)** *Vrn1 ful2* (underlined *Vrn1* indicates one functional copy of *Vrn-A1* in heterozygous state)*.* **(G** and **H)** *vrt2 Vrn1 ful2.* **(A** and **C)** Images of complete indeterminate inflorescences in the absence of functional *vrn1* and *ful2* **(E, G)** Images of determinate inflorescences (terminal spikelet) in the presence of one functional copy of *Vrn-A1*. (**B** and **D)** Detail of the middle section of the inflorescence showing lateral meristems with vegetative characteristics. (**B**) Yellow arrows indicate axillary meristems that were soon covered by elongating leaf primordia. (**C**) Orange arrows indicate axillary floral meristems more developed than in B. **(F** and **H)** Detail of the middle of the inflorescence showing normal spikelet development and no differences between *Vrn1 ful2* and *vrt2 Vrn1 ful2*.

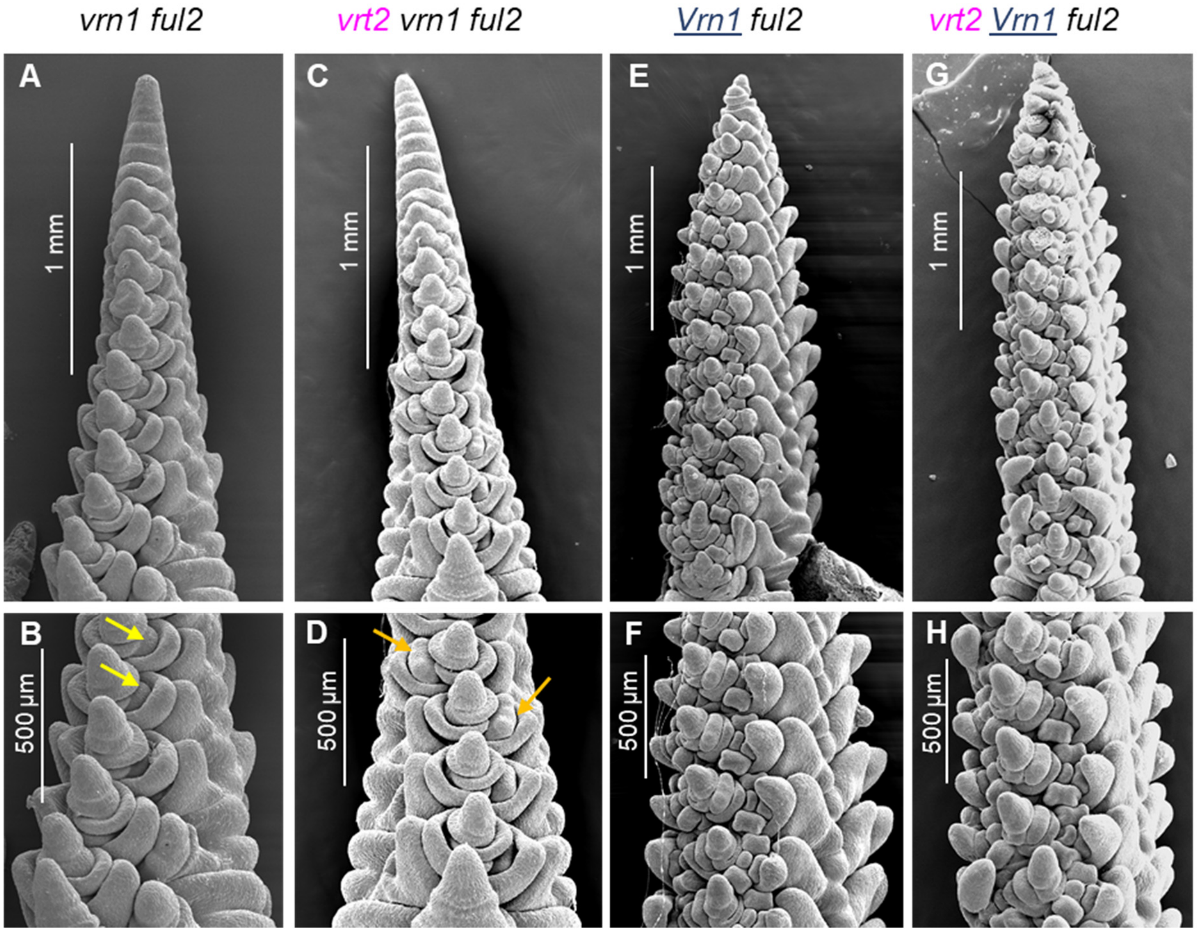

Supplemental Figure S11. Effect of the *vrt2* mutation in the partial mutant *Vrn1 ful2* (the underline indicates a plant heterozygous for *Vrn-A1 vrn-A1* and homozygous *vrn-B1 vrn-B1*). (Supports Figure 10.)

(A) Days to heading. (**B**) Stem length excluding heads. (**C**) Spikelet number per spike. (**D**) Glume length. (**E**) Lemma length. (**F**) Proportion of spikelets transformed into branches with florets replaced by spikelets. The proportion of branched spikelets per spike was calculated from 7-8 spikelets per plant and then averaged across 7 -8 plants. ** = $P < 0.01$, *** = $P < 0.001$ (*t* test) (Supplemental Data Set 2). N = number of plants in A-C & F, spikelets in D and E). Box-plot features are explained in the Statistical analyses section of Material and Methods.

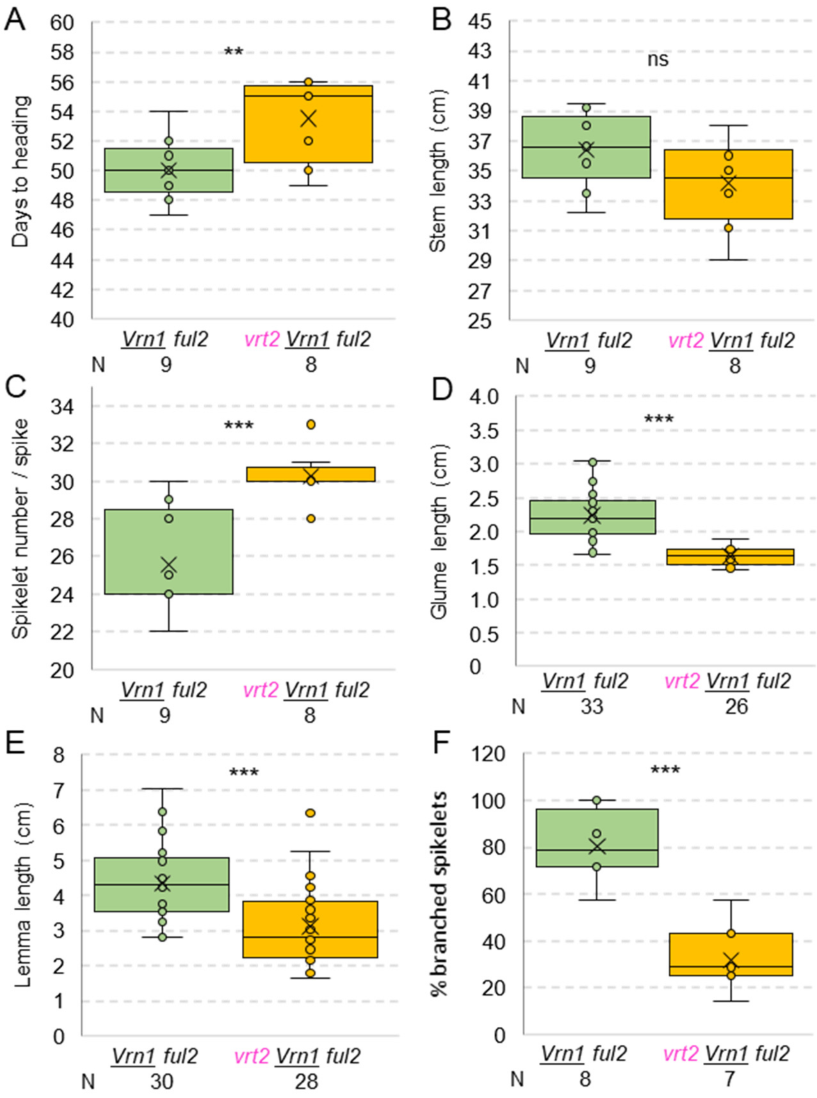

Supplemental Figure S12. Effect of *VRT2* and *SVP1* on the transcript levels of *SEPALLATA*, *CEN* and *TB1* genes in developing spikes. (Supports Figure 10.)

(A) Comparison between Vrn1 *ful2* (*Vrt2*) and *vrt2* Vrn1 *ful2* (*vrt2*) at the post double ridge (PDR) and terminal spikelet (TS) stages. **(B)** Comparison among WT, *vrt2*, *svp1* and *vrt2 svp1* at PDR. Transcript levels were determined by qRT-PCR using *ACTIN* as endogenous control and the ΔCt method. No asterisk = non-significant, * = $P < 0.05$, ** = $P < 0.01$, *** = $P < 0.001$ (*t*Test in (A) and Dunnett test vs. WT in (B)) (Supplemental Data Set 2). Graphs are based on 4 biological replicates (each replicate is a pool of 6-8 developing spikes). Box-plot features are explained in the Statistical analyses section of Material and Methods.

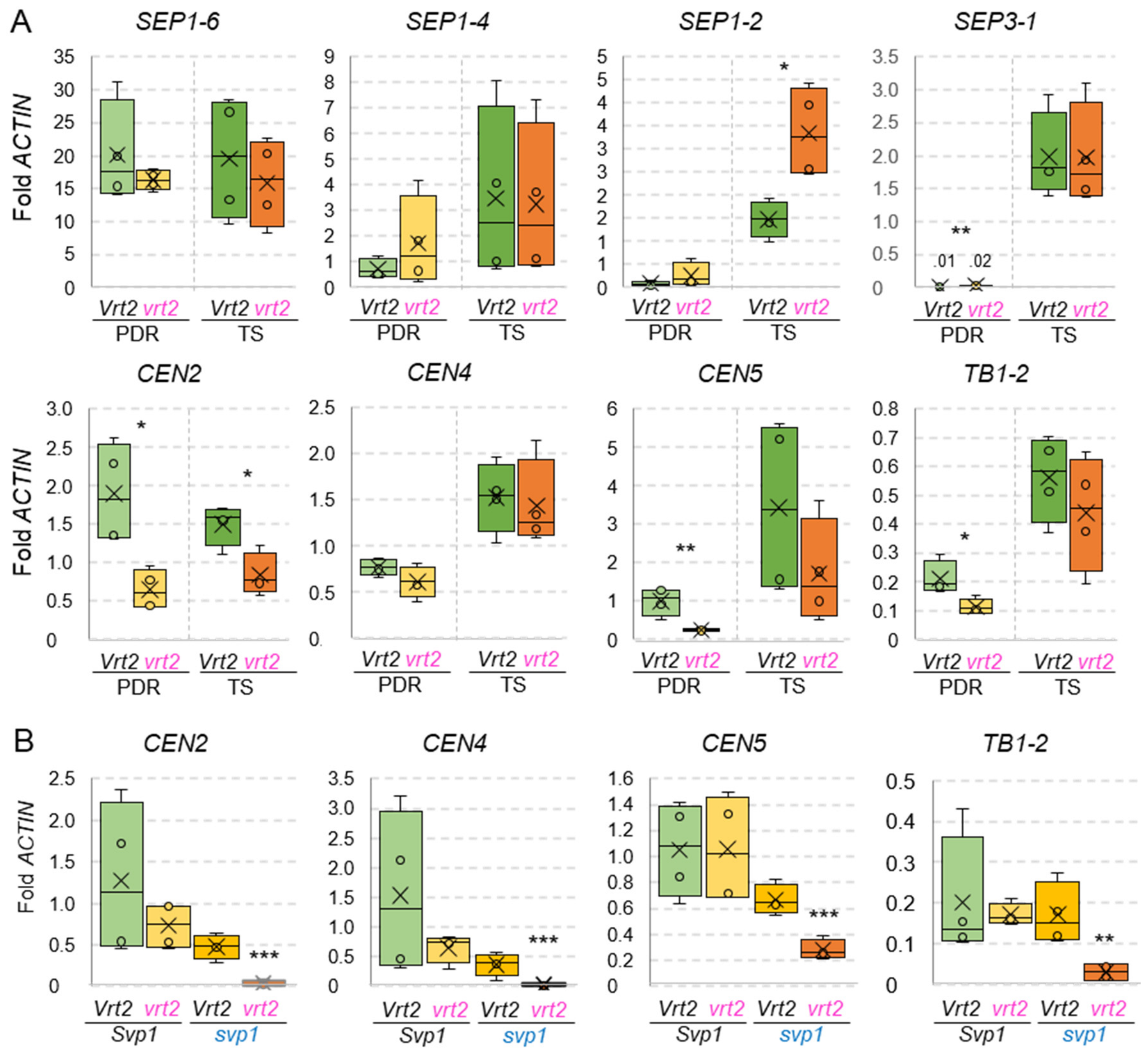

Supplemental Figure S13. Auto-activation tests for the bait and prey vectors used in yeast two-hybrid assays. (Supports Figure 11.)

Vector pGBKT7 expressing the GAL4 DNA binding domain is the empty vector used to generate all the bait vectors, whereas pGADT7 expressing the GAL4 activation domain is the empty vector used to generate all the preys. SD medium lacking Leucine and Tryptophan (-L-W) was used to select for yeast transformants containing both bait and prey vectors. We tested the interactions on SD media lacking Leucine, Tryptophan, Histidine and Adenine (-L-W-H-A).

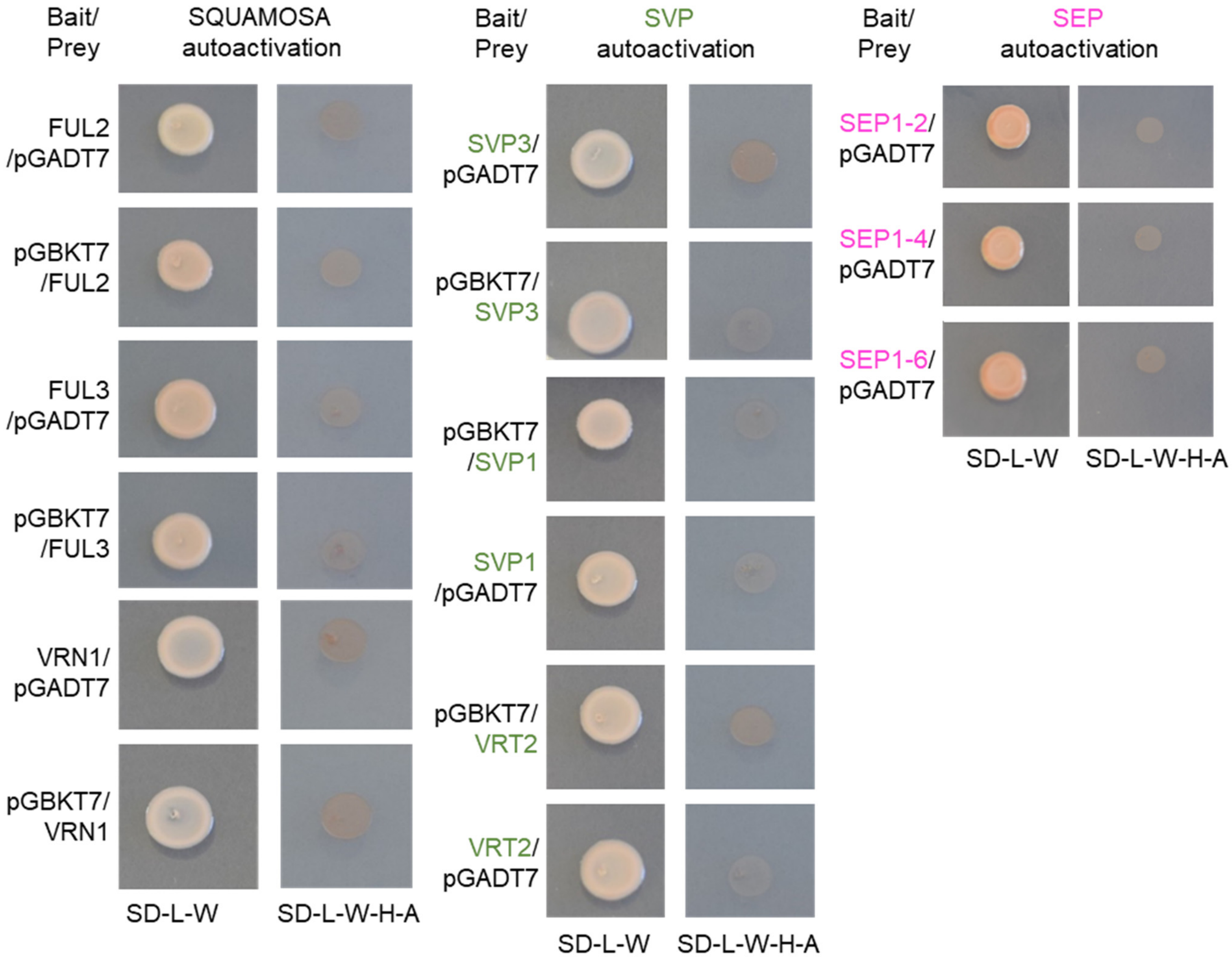

Supplemental Figure S14. Yeast-two-hybrid (Y2H) interactions among proteins within the SQUAMOSA- and SVP-clades. (Supports Figure 11.)

We tested the interactions among the SQUAMOSA MADS-box proteins VRN1, FUL2 and FUL3, and among the SVP proteins VRT2, SVP1 and SVP3. We did not test the interactions among the SEP proteins SEP1-2, SEP1-4, and SEP1-6.

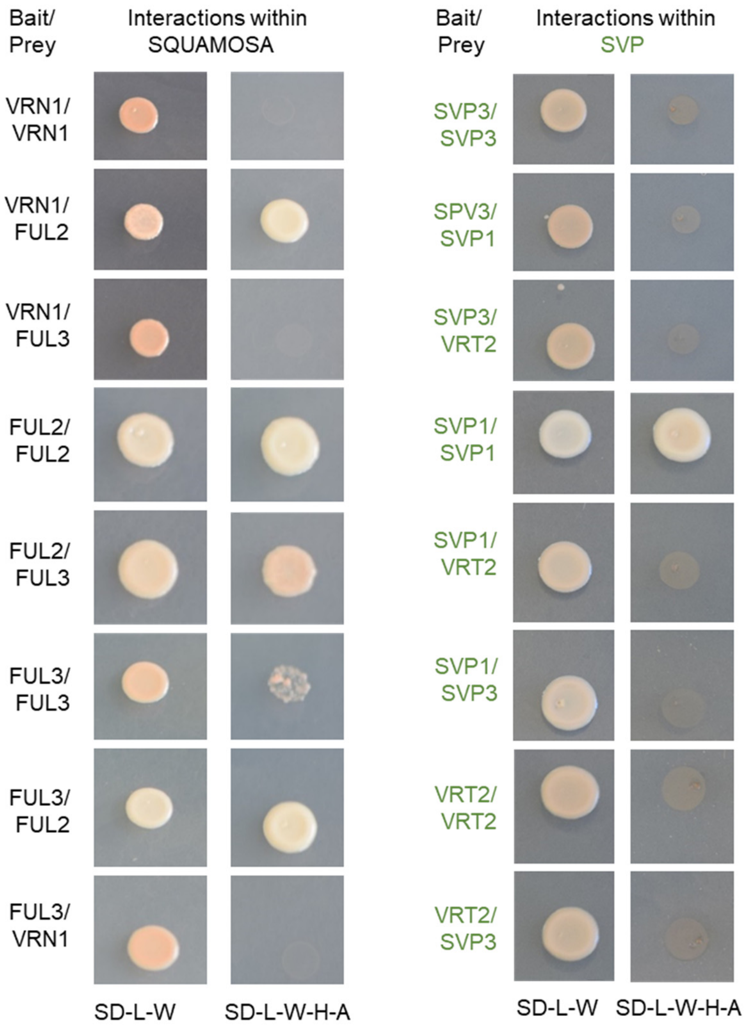

Supplemental Figure S15. Yeast-two-hybrid (Y2H) interactions between wheat MADS-box proteins of the SQUAMOSA (VRN1, FUL2 and FUL3), SVP (VRT2, SVP1 and SVP3) and SEP (SEP1-2, SEP1-4, SEP1-6) classes. (Supports Figure 11.)

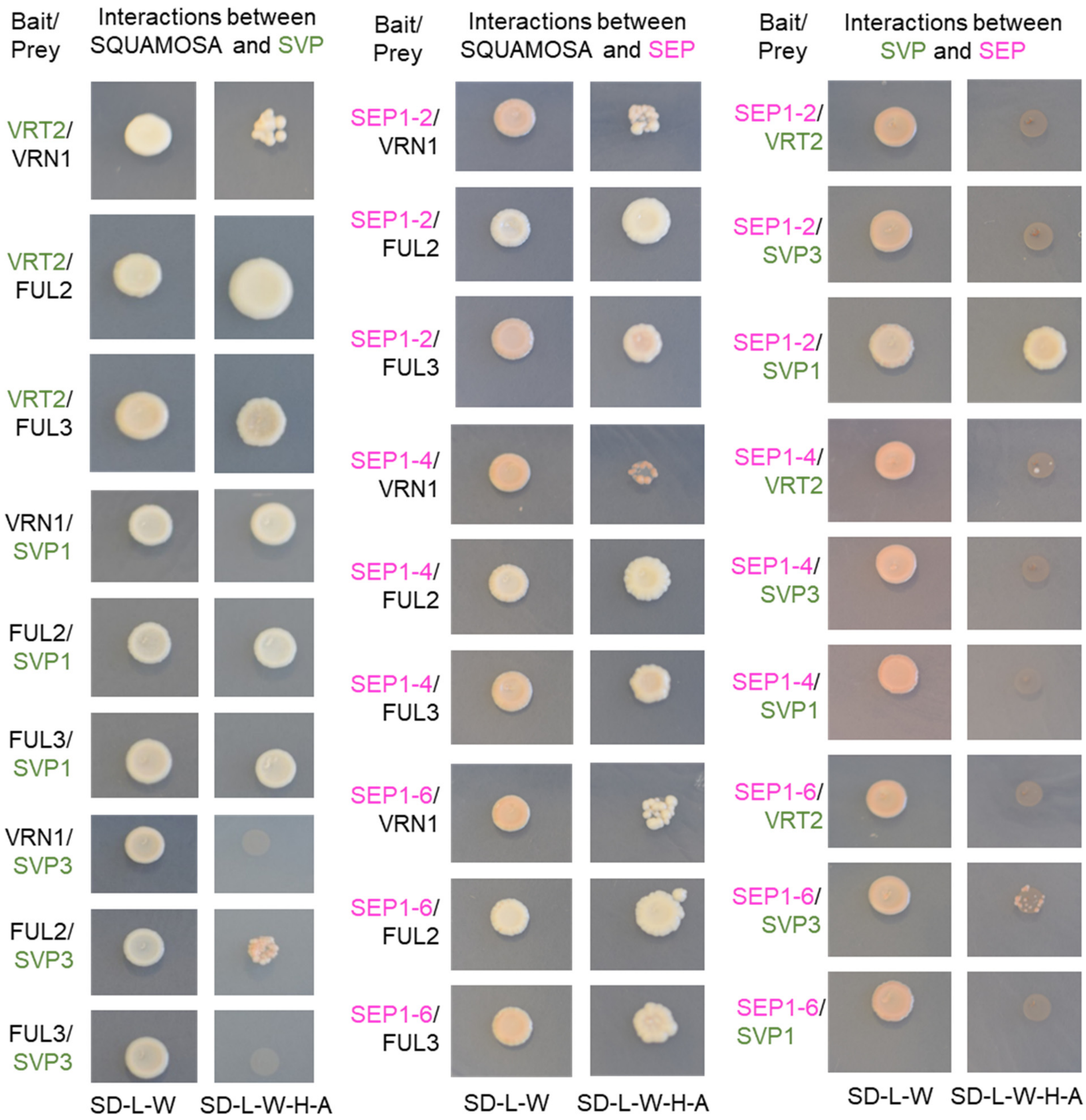

Supplemental Figure S16. Bimolecular Fluorescence Complementation **(**BiFC) between proteins of the SQUAMOSA-clade (VRN1, FUL2 and FUL3) and proteins of both the SVP-clade (VRT2 and SVP1) and SEPALLATA-clade (SEP1-2, SEP1-4 and SEP1-6) in wheat protoplasts. White bars = 200 µm.

The bar is 200 µm. **(A-I)** Positive nuclear signal. **(J-O)** No nuclear signal. **(P-W)** Negative controls.

A) *UBI*$_{pro}$:*N-YFP*:***VRT2*** – *UBI*$_{pro}$:*C-YFP*:***FUL3*** (nuclear signal plus fluorescent aggregates)

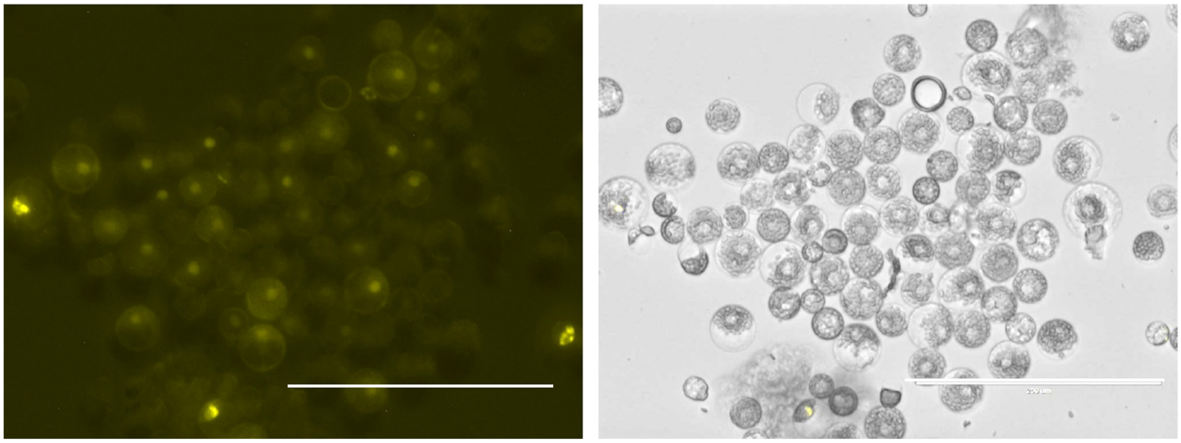

B) *UBI*$_{pro}$:*N-YFP*:***SVP1*** – *UBI*$_{pro}$:*C-YFP*:***FUL3*** (nuclear signal plus fluorescent aggregates)

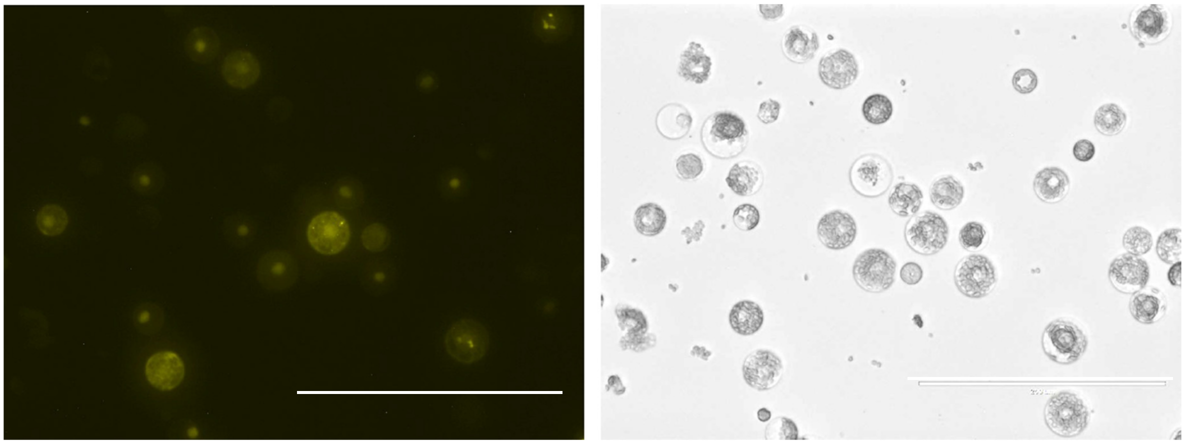

C) *UBI*$_{pro}$:*N-YFP*:***SEP1-2* -** *UBI*$_{pro}$:*C-YFP*:***FUL3*** (nuclear signal plus fluorescent aggregates)

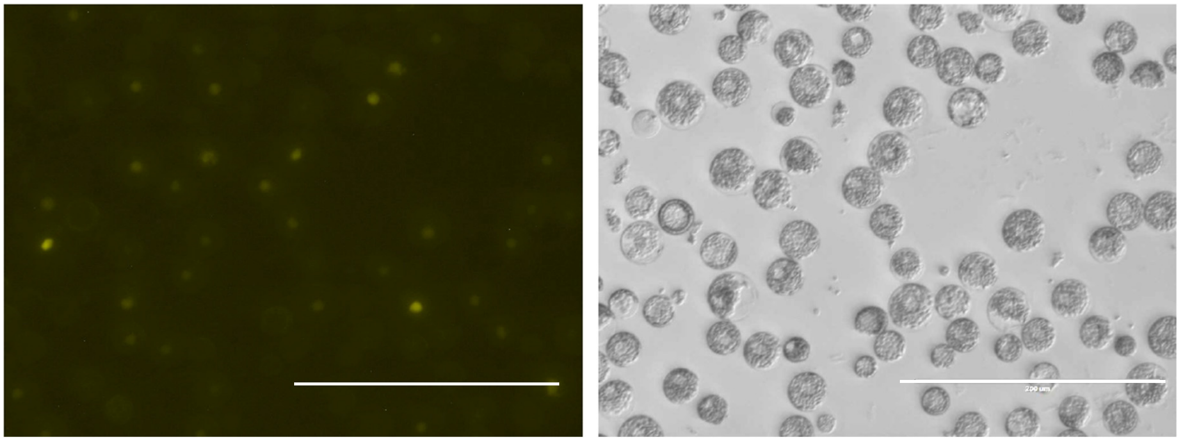

D) UBI_{pro}*:N-YFP:**VRT2*** – UBI_{pro}*:C-YFP:**VRN1*** (nuclear signal plus fluorescent aggregates)

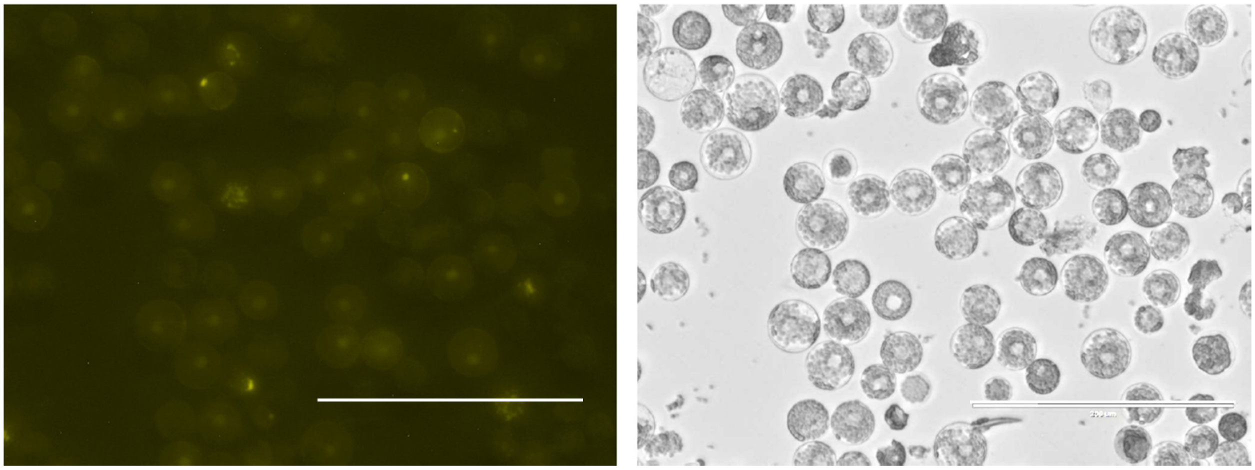

E) UBI_{pro}*:N-YFP:**SVP1*** – UBI_{pro}*:C-YFP:**VRN1*** (nuclear signal plus fluorescent aggregates)

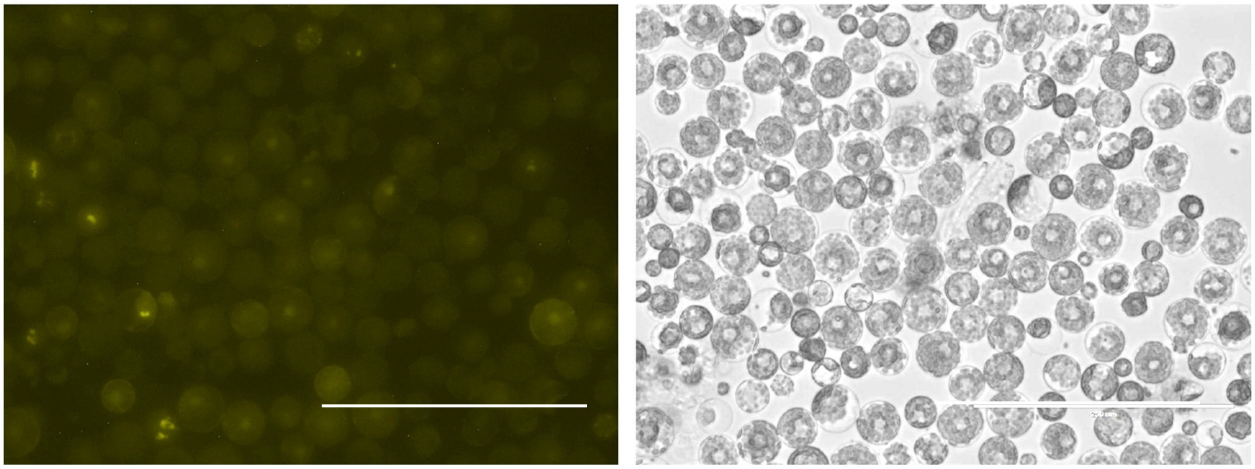

F) UBI_{pro}*:N-YFP:**SEP1-2*** – UBI_{pro}*:C-YFP:**VRN1*** (nuclear signal)

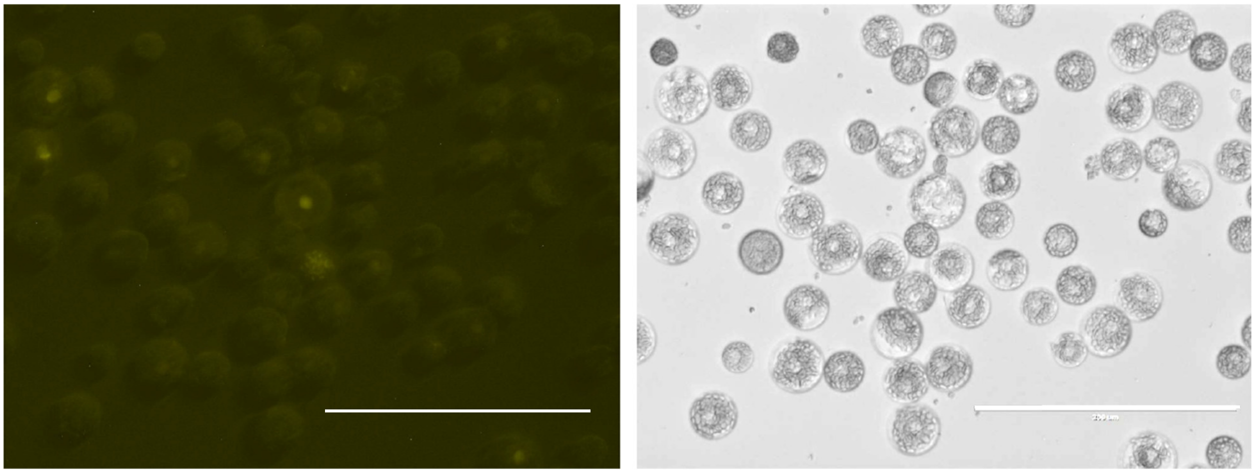

G) *UBI_{pro}:N-YFP:**VRT2*** – *UBI_{pro}:C-YFP:**FUL2*** (nuclear signal plus fluorescent aggregates[1])

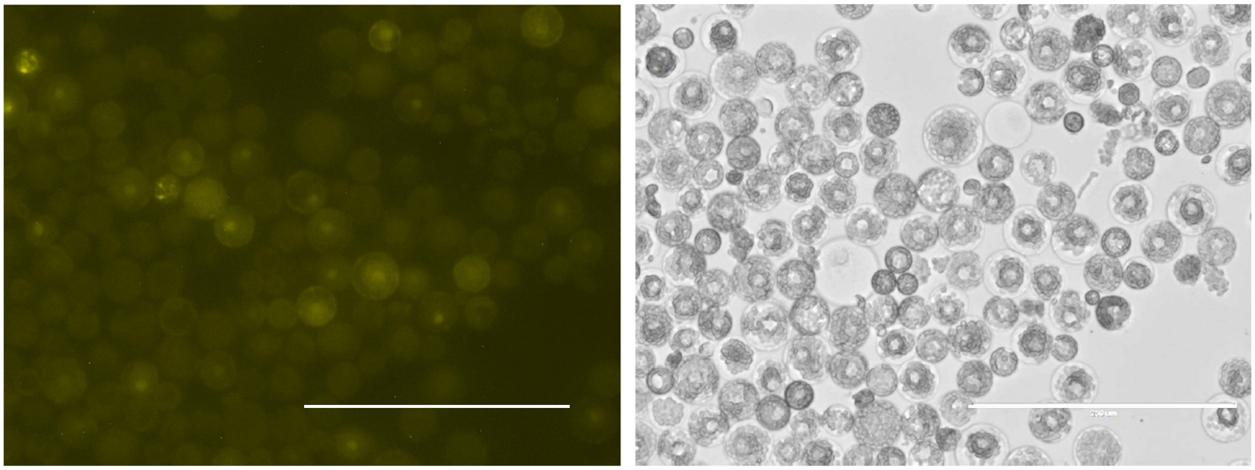

*UBI_{pro}:N-YFP:**VRT2*** – *UBI_{pro}:C-YFP:**FUL2*** fluorescent aggregates outside nucleus[1].

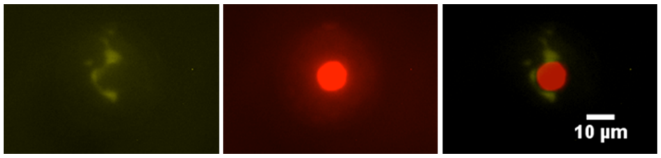

FDL2 mCherry as nuclear marker.

H) *UBI_{pro}:N-YFP:**SVP1*** **–** *UBI_{pro}:C-YFP:**FUL2*** (nuclear signal indicated by arrows & fluorescent aggregates[1])

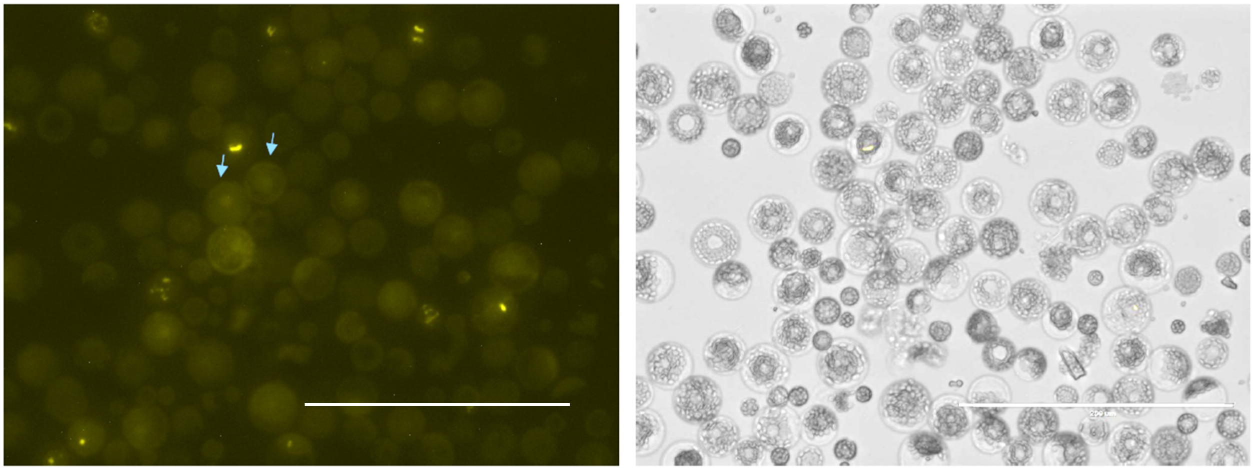

*UBI_{pro}:N-YFP:**SVP1*** – *UBI_{pro}:C-YFP:**FUL2*** fluorescent aggregates outside the nucleus[1].

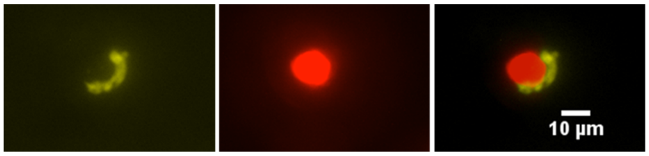

FDL2 mCherry as nuclear marker.

[1] We currently do not know if these bright aggregates are the result of the stabilization of the MADS-box complexes by the YFP reassembly (Robida and Kerppola, 2009, J. Mol. Biol. 394: 391-409), a technical artifact of the over-expression, or a reflection of the real distribution of these MADS-box complexes in the wheat protoplasts.

I) *UBI_{pro}:N-YFP:**SEP1-2*** – *UBI_{pro}:C-YFP:**FUL2***

Nuclear signal indicated by arrows plus fluorescent aggregates.

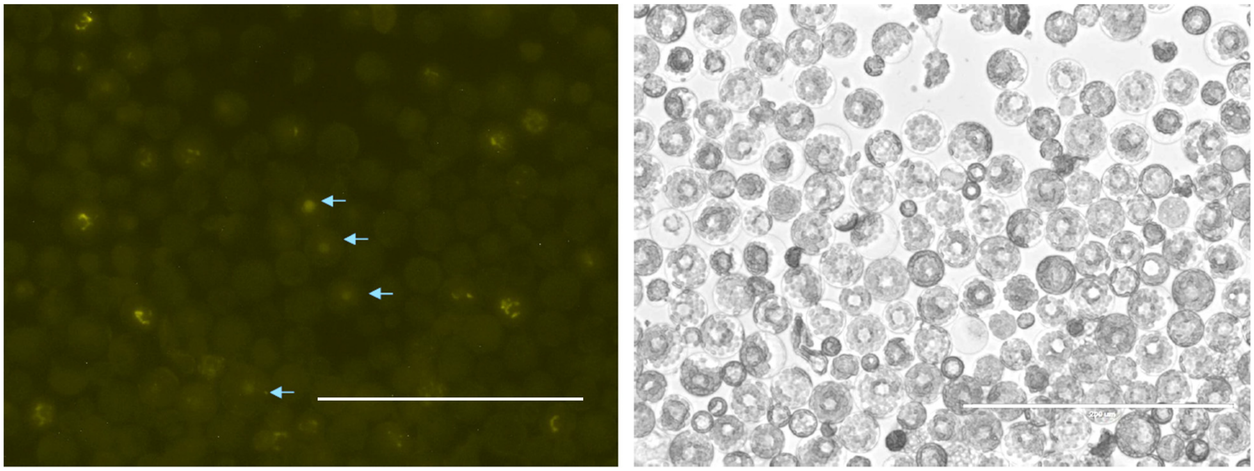

J) *UBI_{pro}:N-YFP:**SEP1-4*** – *UBI_{pro}:C-YFP:**FUL2*** (only fluorescent aggregates)

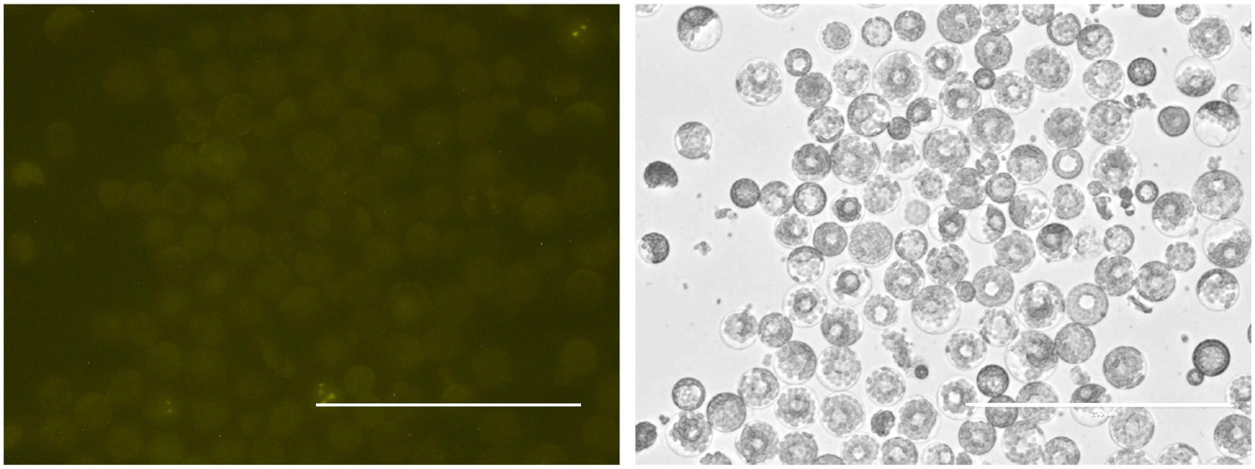

K) *UBI_{pro}:N-YFP:**SEP1-6*** – *UBI_{pro}:C-YFP:**FUL2*** (only fluorescent aggregates)

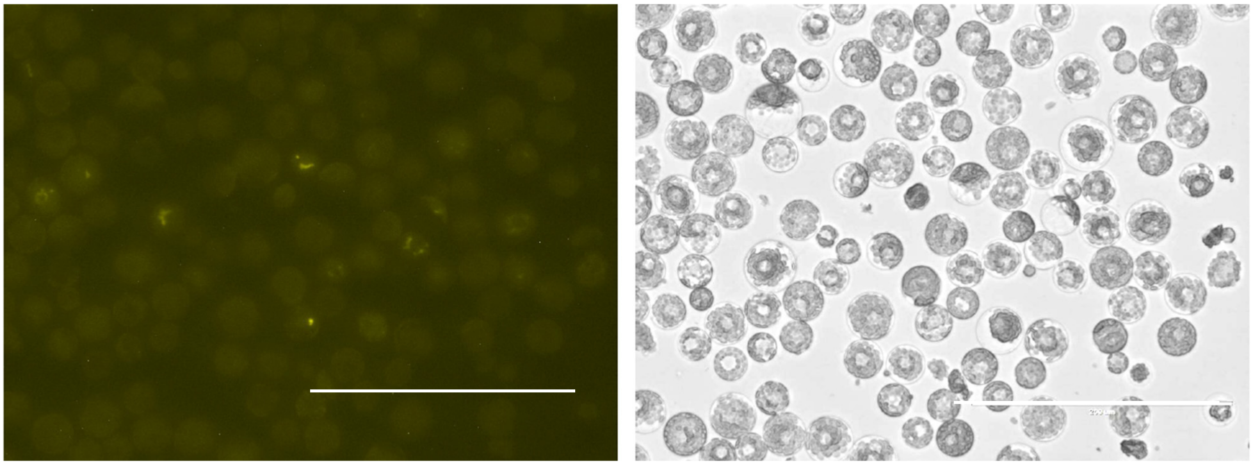

L) *UBI_{pro}:N-YFP:**SEP1-6*** – *UBI_{pro}:C-YFP:**VRN1*** (only fluorescent aggregates)

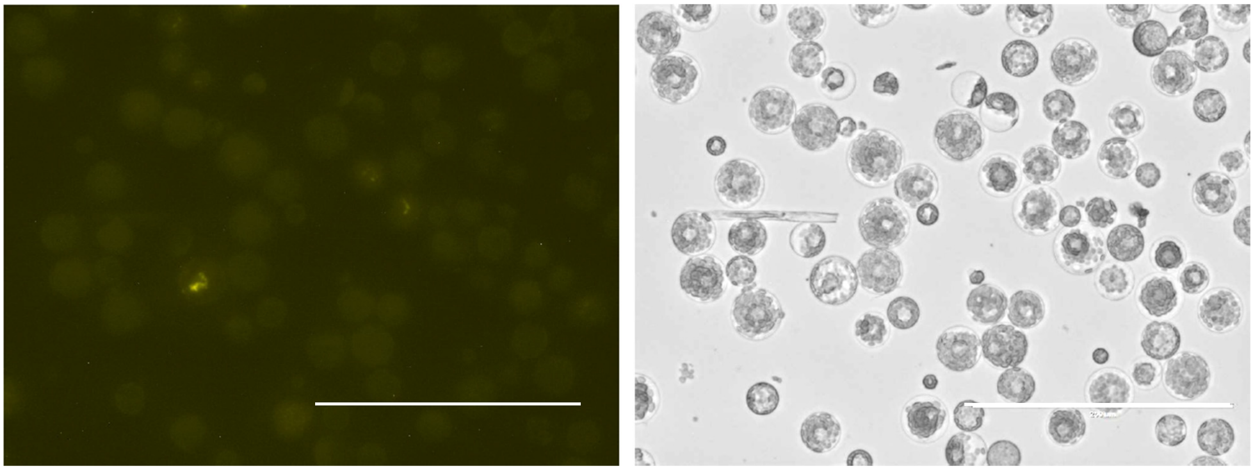

M) *UBI_{pro}:N-YFP:**SEP1-4*** – *UBI_{pro}:C-YFP:**VRN1*** (no signal)

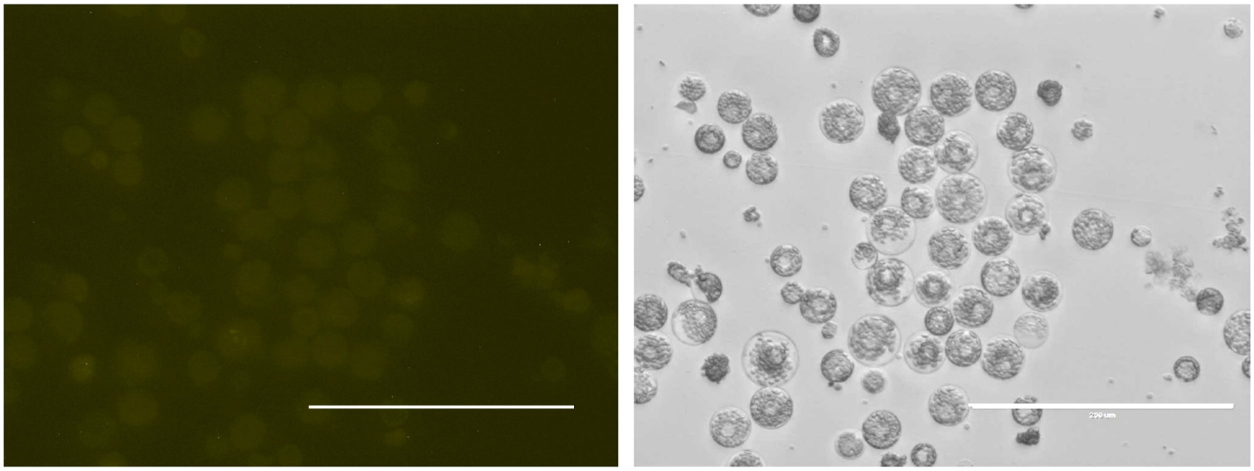

N) *UBI_{pro}:N-YFP:**SEP1-4*** – *UBI_{pro}:C-YFP:**FUL3*** (no signal)

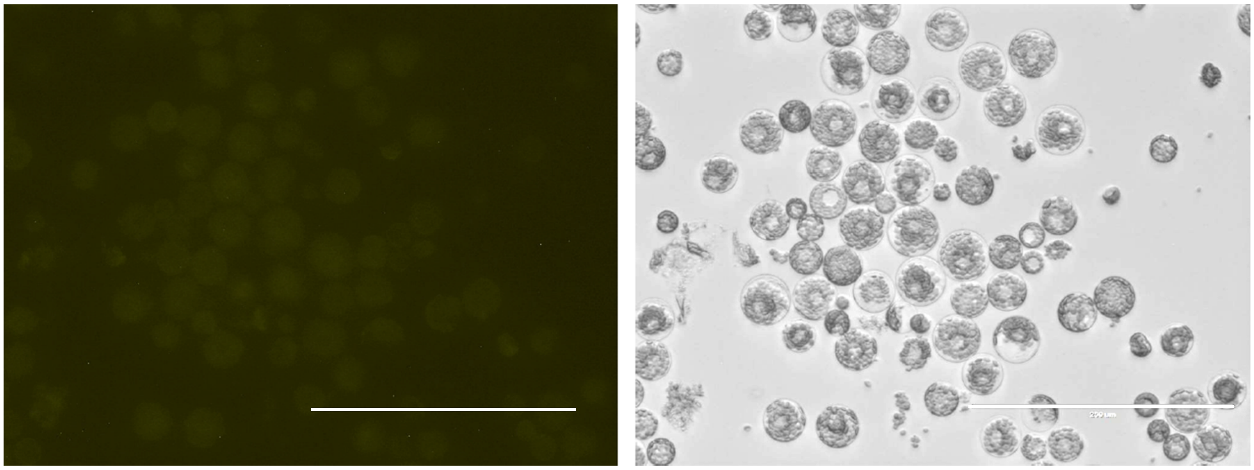

O) *UBI_{pro}:N-YFP:***SEP1-6** – *UBI_{pro}:C-YFP:***FUL3** (no signal)

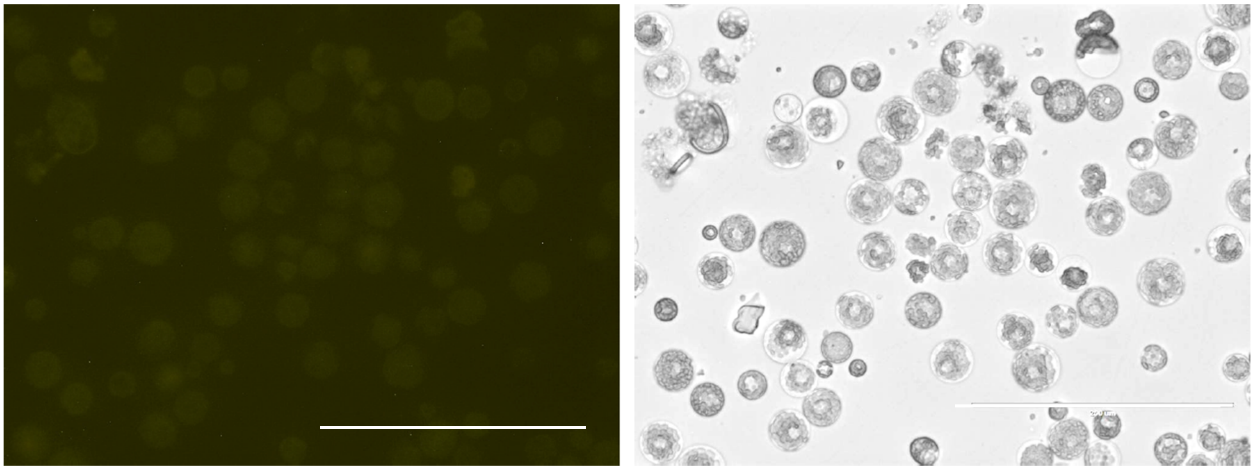

BiFC negative controls

P) *UBI_{pro}:N-YFP:***VRT2** – *C-YFP* (negative control, no signal)

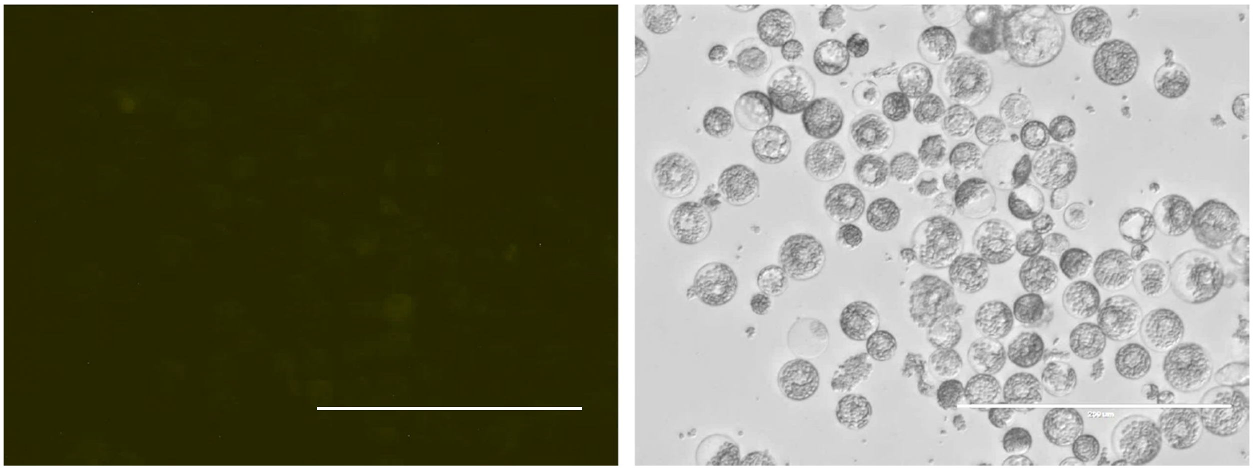

Q) *UBI_{pro}:N-YFP:***SVP1** – *C-YFP* (negative control, no signal)

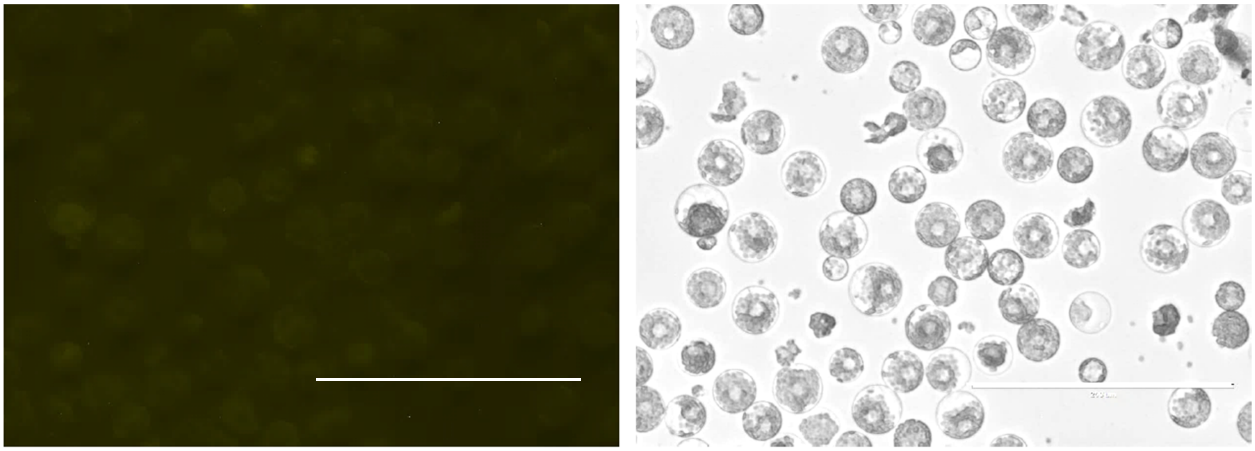

R) *UBI$_{pro}$:N-YFP:***FUL3***- C-YFP* (negative control, no signal)

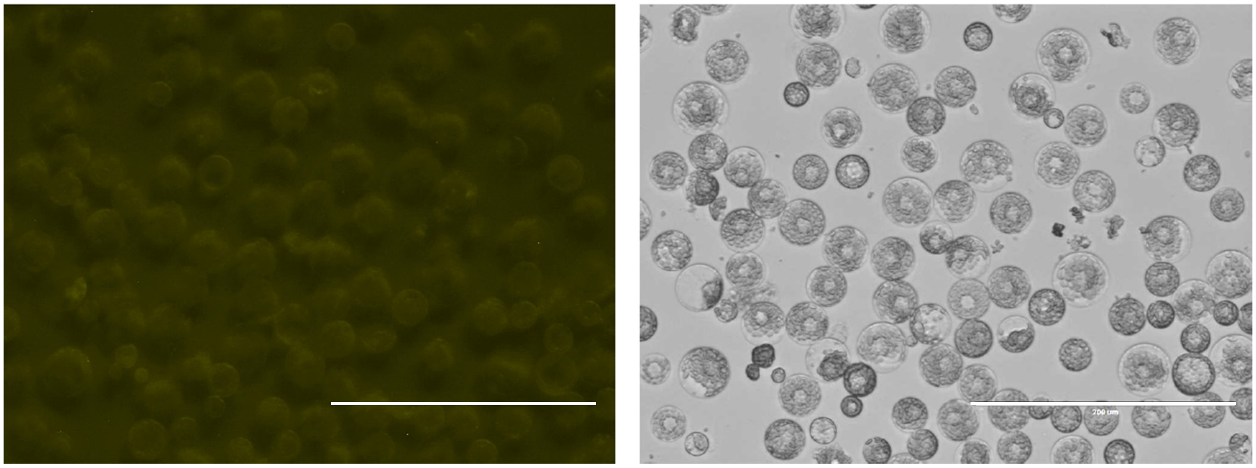

S) *UBI$_{pro}$:N-YFP:***FUL2** – *C-YFP* (negative control, no signal)

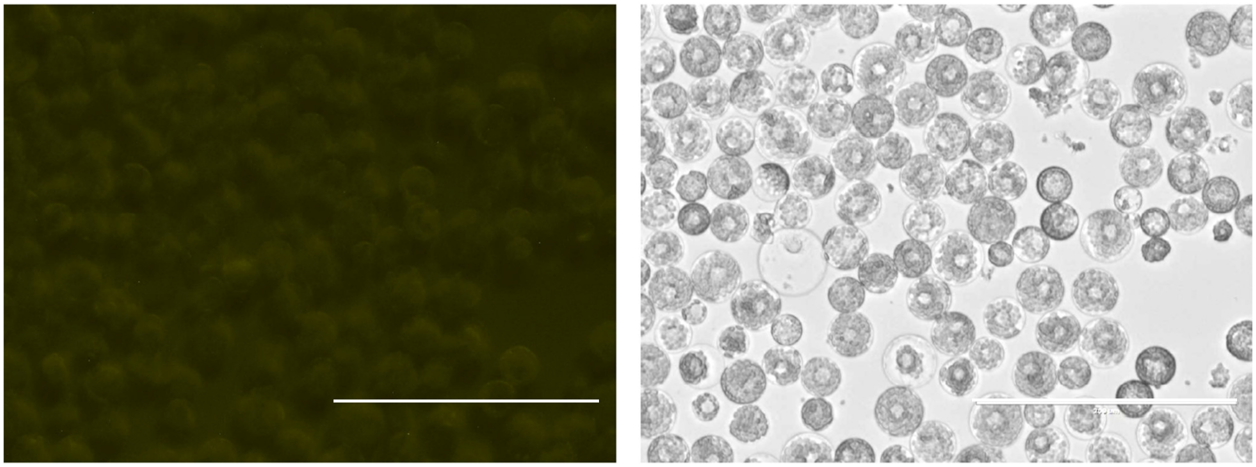

T) *UBI$_{pro}$:N-YFP:***VRN1** – *C-YFP* (negative control, no signal)

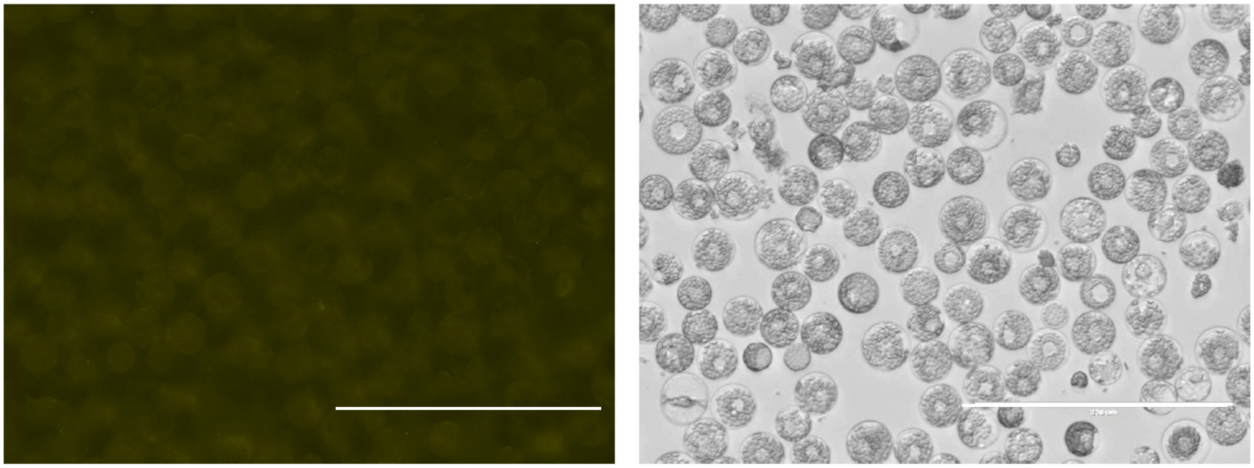

U) *UBI_{pro}:N-YFP:**SEP1-2*** – *C-YFP* (negative control, no signal)

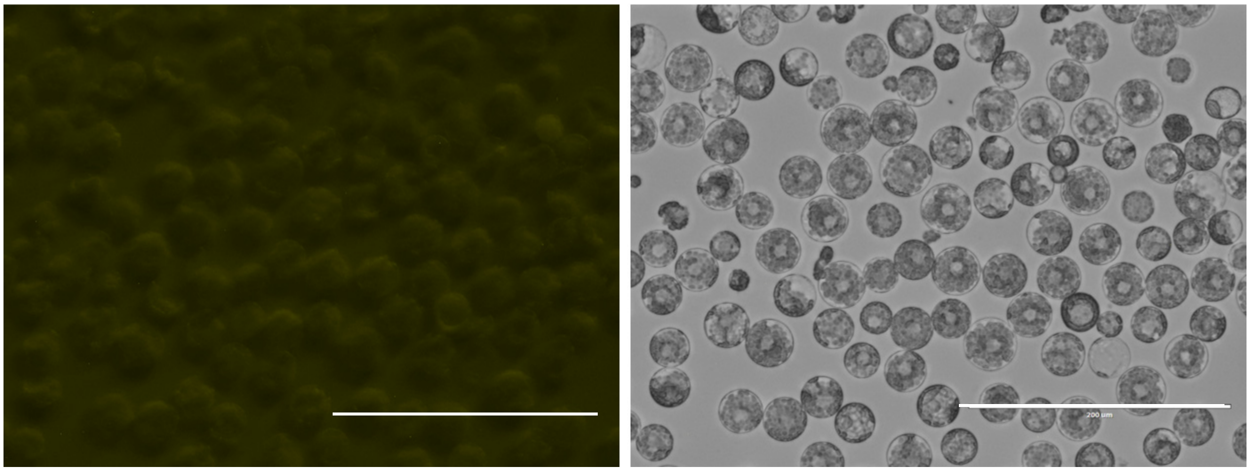

V) *UBI_{pro}:N-YFP:**SEP1-4*** – *C-YFP* (negative control, no signal)

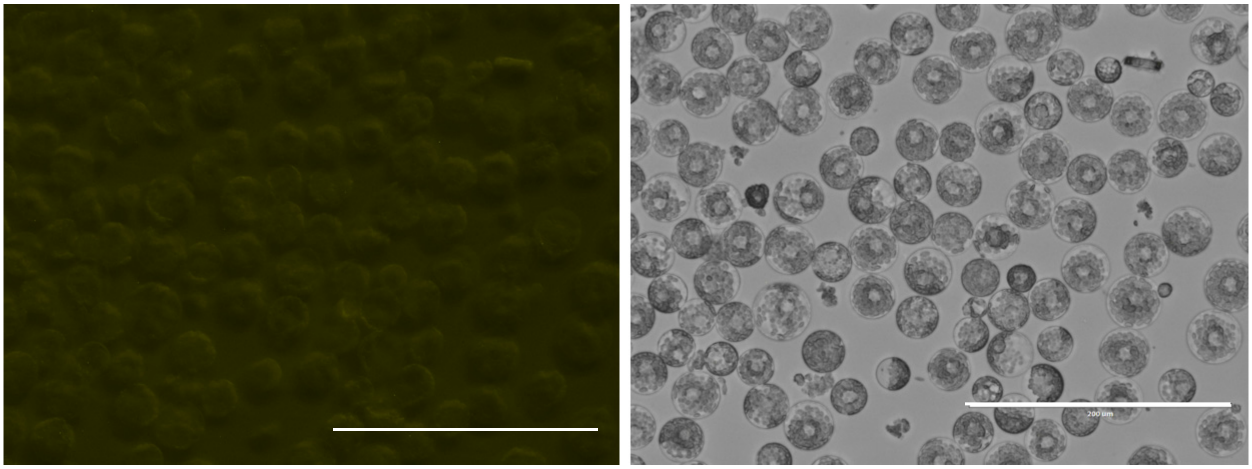

W*)* *UBI_{pro}:N-YFP:**SEP1-6*** – *C-YFP* (negative control, no signal)

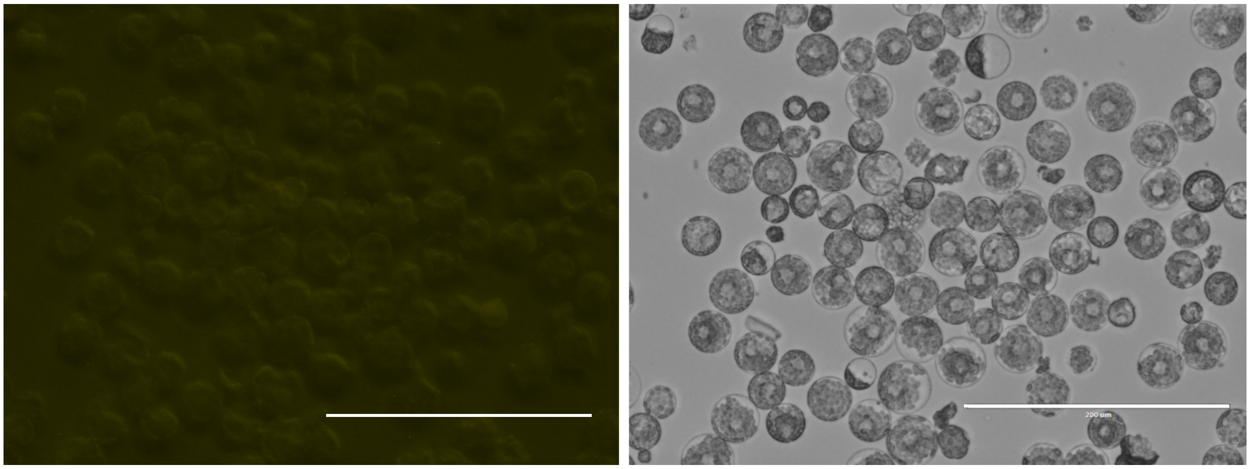

SUPPLEMENTAL TABLES

Supplemental Table S1. Nomenclature, accession numbers, synonyms of wheat genes used in this study and their rice orthologs. We adopted the wheat MADS-box nomenclature proposed in a recent and exhaustive review (1) except for the *VRN1* (2) and *VRT2* (3) genes, for which the historical names were used, and *FUL2* and *FUL3*, for which a comprehensive phylogenetic analysis and nomenclature has been proposed before (4). *BM* names correspond to barely MADS-box gene names.

MADS		Wheat RefSeq v1.1					
Class	Rice	A genome	B genome	**Wheat**	Ref.	Synonyms	Ref.
**SVP**	*OsMADS22*	*TraesCS6A02G313800.1*	*TraesCS6B02G343900.1*	*SVP1*	(1)	*BM10* [a]	(5)
	OsMADS47	*TraesCS4A02G002600.1*	*TraesCS4B02G302600.1*	*SVP3*	(1)	*BM1* [a]	(5)
	OsMADS55	*TraesCS7A02G175200.1*	*TraesCS7B02G080300.1*	*VRT2*	(3)	*TaSVP-2*	(1)
**A-class**	*OsMADS14*	*TraesCS5A02G391700.1*	*TraesCS5B02G396600.1*	*VRN1*	(2)	*TaMADS11*<br>*BM5* [a]<br>*TaFUL1*<br>*TaAP1-1*	(6)<br>(5)<br>(7)<br>(1)
	OsMADS15	*TraesCS2A02G261200.1*	*TraesCS2B02G281000.1*	*FUL2*	(7)	*BM8* [a]<br>*TaAP1-3*	(5)<br>(1)
	OsMADS18	*TraesCS2A02G174300.1*	*TraesCS2B02G200800.1*	*FUL3*	(7)	*BM3* [a]<br>*TaAP1-2*	(5)<br>(1)
**B-class**	*OsMADS4*	*TraesCS1A02G264300.1*	*TraesCS1B02G275000.1*	*PI1*	(1)	*WPI-1*	(8)
	OsMADS16	*TraesCS7A02G383800.1*	*TraesCS7B02G286600.1*	*AP3-1*	(1)	*TaMADS51*<br>*WAP3*	(6)<br>(8)
**C-class**	*OsMADS3*	*TraesCS3A02G314300.1*	*TraesCS3B02G157500.1*	*AG2*	(1)	*WAG-2*	(9)
	OsMADS58	*TraesCS1A02G125800.1*	*TraesCS1B02G144800.1*	*AG1*	(1)	*WAG-1*	(9)
**E-class**	*OsMADS1*	(*TraesCS4A02G058900.1*) [b]	*TraesCS4B02G245700.1*	*SEP1-2*	(1)	*BM7* [a]<br>*WLHS1*<br>*TaSEP2*	(5)<br>(10)<br>(11)
	OsMADS34	*TraesCS5A02G391800.1*	*TraesCS5B02G396700.1*	*SEP1-6*	(1)	*TaAGLG1*<br>*PAP2*<br>*TaSEP5*	(2)<br>(12)<br>(11)
	OsMADS5	*TraesCS7A02G122000.1*	*TraesCS7B02G020800.1*	*SEP1-4*	(1)	*TaSEP6*	(11)
	OsMADS7	*TraesCS7A02G260600.1*	*TraesCS7B02G158600.1*	*SEP3-1*	(1)	*WSEP*<br>*TaSEP4*	(10)<br>(11)
	OsMADS8	*TraesCS5A02G286800.1*	*TraesCS5B02G286100.1*	*SEP3-2*	(1)	*TaSEP3*	(11)

[a] Barley gene name. All other names in this column are for wheat genes

[b] Pseudogene in Chinese Spring but complete and expressed gene in Kronos

References Supplemental Table S1

1. **Schilling, S., Kennedy, A., Pan, S., Jermiin, L.S., and Melzer, R.** (2020) Genome-wide analysis of MIKC-type MADS-box genes in wheat: pervasive duplications, functional conservation and putative neofunctionalization. New Phytol. **225**: 511-529.

2. **Yan, L., Loukoianov, A., Tranquilli, G., Helguera, M., Fahima, T., and Dubcovsky, J.** (2003) Positional cloning of wheat vernalization gene *VRN1*. Proc. Natl. Acad. Sci. U.S.A. **100**, 6263-6268.

3. **Kane, N.A., Danyluk, J., Tardif, G., Ouellet, F., Laliberte, J.F., Limin, A.E., Fowler, D.B., and Sarhan, F.** (2005) TaVRT-2, a member of the StMADS-11 clade of flowering repressors, is regulated by vernalization and photoperiod in wheat. Plant Physiol. **138**: 2354-2363.

4. **Preston, J.C., and Kellogg, E.A.** (2007) Conservation and divergence of *APETALA1/FRUITFULL*-like gene function in grasses: evidence from gene expression analyses. Plant J. **52** :69-81.

5. **Schmitz, J., Franzen, R., Ngyuen, T.H., Garcia-Maroto, F., Pozzi, C., Salamini, F., and Rohde, W.** (2000) Cloning, mapping and expression analysis of barley MADS-box genes. Plant Mol. Biol. **42**: 899-913.

6. **Murai, K., Murai, R., Takumi, S., and Ogihara, Y.** (1998) cDNA cloning of three MADS box genes in wheat (Accession Nos. AB007504, AB007505 and AB007506). Plant Physiol. **118**: 330.

7. **Preston, J.C., and Kellogg, E.A.** (2006) Reconstructing the evolutionary history of paralogous *APETALA1/FRUITFULL*-like genes in grasses (Poaceae). Genetics **174**: 421-437.

8. **Hama, E., Takumi, S., Ogihara, Y., and Murai, K.** (2004) Pistillody is caused by alterations to the class-B MADS-box gene expression pattern in alloplasmic wheats. Planta **218**: 712-720.

9. **Hirabayashi, C., and Murai, K.** (2009) Class C MADS-box gene AGAMOUS was duplicated in the wheat genome. Wheat Information Service **107**: 13-16.

10. **Shitsukawa, N., Tahira, C., Kassai, K.I., Hirabayashi, C., Shimizu, T., Takumi, S., Mochida, K., Kawaura, K., Ogihara, Y., and Murai, K.** (2007) Genetic and epigenetic alteration among three homoeologous genes of a class E MADS box gene in hexaploid wheat. Plant Cell **19**: 1723-1737.

11. **Paolacci, A.R., Tanzarella, O.A., Porceddu, E., Varotto, S., and Ciaffi, M.** (2007) Molecular and phylogenetic analysis of MADS-box genes of MIKC type and chromosome location of *SEP*-like genes in wheat (*Triticum aestivum* L.). Mol. Genet. Genomics **278**: 689-708.

12. **Kobayashi, K., Maekawa, M., Miyao, A., Hirochika, H., and Kyozuka, J.** (2010) *PANICLE PHYTOMER2* (*PAP2*), encoding a SEPALLATA subfamily MADS-box protein, positively controls spikelet meristem identity in rice. Plant Cell Physiol. **51**: 47-57.

Supplemental Table S2. Summary statistics for the Quant-Seq samples. 100 bp not-paired reads were obtained from Hi-Seq.

Genotype	Stage	Rep.	Raw Seq.	Post filtering & trimming Seq.	Avg. read length	% GC	Avg. quality per read
vrn1 ful2	Vegetative	1	8,908,554	8,133,600	74.6	44	36.4
vrn1 ful2	Vegetative	2	8,712,913	7,941,270	73.9	44	36.5
vrn1 ful2	Vegetative	3	8,058,165	7,421,715	74.4	44	36.4
vrn1 ful2	Vegetative	4	6,645,853	6,095,630	74.4	43	36.3
vrn1 ful2	Double Ridge	1	7,410,731	6,806,199	74.5	44	36.5
vrn1 ful2	Double Ridge	2	7,917,524	7,322,349	74.7	44	36.5
vrn1 ful2	Double Ridge	3	7,107,594	6,531,596	74.3	44	36.4
vrn1 ful2	Double Ridge	4	8,690,383	8,001,221	74.4	44	36.5
vrn1 ful2	Post Double Ridge	1	7,815,528	7,129,083	74.3	44	36.5
vrn1 ful2	Post Double Ridge	2	8,229,842	7,487,536	73.8	45	36.5
vrn1 ful2	Post Double Ridge	3	7,924,170	7,292,702	74.6	44	36.4
vrn1 ful2	Post Double Ridge	4	7,434,355	6,776,312	74.0	44	36.6
vrn1 ful2	Terminal spikelet	1	7,914,775	7,224,431	73.8	45	36.2
vrn1 ful2	Terminal spikelet	2	9,227,663	8,530,373	74.7	44	36.5
vrn1 ful2	Terminal spikelet	3	7,132,618	6,540,557	74.2	44	36.4
vrn1 ful2	Terminal spikelet	4	8,463,345	7,787,485	74.5	44	36.5
vrn1	Vegetative	1	8,116,199	7,382,112	74.0	44	36.5
vrn1	Vegetative	2	8,248,815	7,526,889	74.4	44	36.4
vrn1	Vegetative	3	8,678,594	7,838,265	73.7	44	36.4
vrn1	Vegetative	4	7,024,474	6,368,904	73.6	43	36.7
vrn1	Double Ridge	1	7,629,003	6,983,084	74.3	44	36.4
vrn1	Double Ridge	2	6,081,080	5,525,915	73.3	44	36.1
vrn1	Double Ridge	3	7,770,006	7,209,184	74.7	44	36.5
vrn1	Double Ridge	4	7,224,717	6,630,339	74.4	44	36.5
vrn1	Post Double Ridge	1	6,348,081	5,874,810	76.3	44	36.3
vrn1	Post Double Ridge	2	8,990,467	8,144,957	73.6	45	36.5
vrn1	Post Double Ridge	3	8,151,534	7,518,656	74.6	44	36.5
vrn1	Post Double Ridge	4	8,287,973	7,593,081	74.2	45	36.3
vrn1	Terminal spikelet	1	7,635,487	6,996,867	74.3	43	36.5
vrn1	Terminal spikelet	2	9,248,399	8,497,189	74.1	44	36.6
vrn1	Terminal spikelet	3	10,025,819	9,043,389	73.6	44	36.6
vrn1	Terminal spikelet	4	9,251,175	8,571,176	74.9	43	36.5
	**Average**		**8,009,557**	**7,335,215**	**74.3**	**44.0**	**36.4**

Supplemental Table S3. Differentially Expressed Genes (DEGs) between *vrn1* and *vrn1 ful2* at different developmental stages of spike development: VEG= vegetative, DR= double ridge, PDR= post double ridge, TS= terminal spikelet. Complete list of DEGs at FDR 0.05 in Supplementary File 1. DEGs with known functions in inflorescence or flower development in grasses, with corresponding references and short functional description. DEGs are grouped by expression clusters as described in Figure 1. The X indicates effect on spikelet number per spike or panicle (SNS) or spikelet and floret organ identity (O. ID).

Gene	Name/Symbol	Sp	Ref.	Description	Function	
					SNS	O. ID
**Cluster 4**						
TraesCS2A02G116900<br>TraesCS2B02G136100	*BRANCHED HEAD1*<br>*COMPOSITUM2*<br>*BRANCHED SILKLESS*<br>*FRIZZY PANICLE*	*Ta*<br>*Hv*<br>*Zm*<br>*Os*	(1)<br>(2)<br>(3)	Mutations in this gene are associated with branched spikes in wheat and with the replacement of spikelets by branches in rice.	x	
TraesCS1A02G314200<br>TraesCS1B02G326500	*MULTI-FLORET SPIKELET1*	*Os*	(4)	Involved in the regulation of spikelet meristem determinacy and floral organ identity in rice.	x	x
TraesCS3A02G350600<br>TraesCS3B02G383000	*LAX PANICLE1*<br>*BARREN STALK1*	*Os*<br>Zm	(5)<br>(6)	Required for the initiation/maintenance of axillary meristems in rice and maize inflorescence (branches and flowers).		
TraesCS2A02G232400<br>TraesCS2B02G250900	*SPL13*	*Os*	(7)	Involved in regulation of panicle length, number of primary branches and grain size in rice.	x	
TraesCS5A02G319200	*SIX-ROWED SPIKE 2*	*Hv*	(8)	The barley *vrs2* mutants develops supernumerary spikelets at its base.	x	
TraesCS5B02G319600	*SHORT INTERNODES1*	*Os*	(9)	The rice *shi1* mutant shows reduced tiller number, enhanced culm strength, and increased panicle branch number.	x	
TraesCS3A02G247200	*DWARF TILLER1*	*Os*	(10)	The rice *dtw1* mutant has short internodes.		
**Cluster 5**						
TraesCS4A02G058900<br>TraesCS4B02G245700	*OsMADS1*<br>*LEAFY HULL STERILE1*	*Os*	(11)	The rice *lhs1* mutant has leafy palea and lemmas.		x
TraesCS7A02G122000<br>TraesCS7B02G020800	*OsMADS5*	*Os*	(12)	*OsMADS1*, *OsMADS5*, and *OsMADS34* together regulate floral meristem determinacy and specify spikelet organs identity.		x
TraesCS5A02G286800<br>TraesCS5B02G286100	*OsMADS8*	*Os*	(13)	Plants affected in both *OsMADS7* and *OsMADS8* show late flowering, homeotic changes of lodicules, stamens and carpels into palea/lemma-like organs, and a loss of floral determinacy.		x
TraesCS7A02G260600<br>TraesCS7B02G158600	*OsMADS7*	*Os*	(13)			
TraesCS1A02G264300	*OsMADS4 / PI1*	*Os*	(14)	These genes control lodicule and stamen development.		x
TraesCS3B02G440200	*OsMADS2 / PI2*	*Os*	(15)			x
TraesCS6A02G259000	*OsMADS6*<br>*MOSAIC FLORAL ORGANS1*	*Os*	(16)	Rice *osmads6* mutant has altered palea identity, extra glume-like or mosaic organs, abnormal carpels and loss of floral meristem determinacy.		x
TraesCS6B02G286400			(17)	*mfo1* mutants have disturbed palea and lodicule identities, and mosaic organs. Determinacy of the floral meristem was lost.		
TraesCS3B02G157500	*OsMADS3*	*Os*	(18)	A knockout line shows homeotic transformation of stamens into lodicules and ectopic development of lodicules in the second whorl near the palea, carpels develop almost normally.		x
TraesCS1A02G125800	*OsMADS58*	*Os*		RNA-silencing of *OSMADS58* results in flowers that reiterate a set of floral organs, including lodicules, stamens, and carpel-like organs.		x

TraesCS3B02G318300	*OsMADS32*	*Os*	(19)	Rice *OsMADS32* plays a role in maintaining floral organ identity.		x
TraesCS2A02G312200<br>TraesCS2B02G329000	*NONSTOP GLUMES1*	*Os*	(20)	Rice *NSG1* plays a pivotal role in maintaining organ identities in the spikelet by repressing the expression of *LHS1*, *DL*, and *MFO1*.		x
TraesCS5A02G134700<br>TraesCS5B02G133700	*LAX-A*	*Hv*	(21)	Barley mutant *lax-a* phenotype has extended rachis internodes, broadened base of the lemma awns, thinner grains and homeotic conversion of lodicules into stamenoid structures. Homolog of *BLADE-ON-PETIOLE1* (*BOP1*) and *BOP2*		
**Cluster 7** (includes *SVP1*, *VRT2* and *SVP3* from this study)						
TraesCS1A02G154900<br>TraesCS1B02G172100	*TAW1*	Os	(22)	Gain-of-function mutant prolongs branch formation and increased numbers of spikelets. Upstream SVP-like genes.	x	
TraesCS2A02G420900<br>TraesCS2B02G440000	*NAL1 / SPIKE*	Os	(23)	Natural variants with increased expression are associated with increases in spikelet number, leaf size, root system, and vascular bundles.	x	
TraesCS1A02G106200<br>TraesCS1B02G123500	*GA2OX1*	Os	(24)	Ectopic expression of the OsGA2ox1 cDNA in transgenic rice inhibited stem elongation and the development of reproductive organs.		
**Cluster 8**						
TraesCS5A02G391800<br>TraesCS5B02G396700	*OsMADS34 / PAP2*	Os	(25)	Rice *pap2-1* mutant shows transformation of early arising spikelets into rachis branches. Rudimentary glumes and sterile lemmas, the outermost organs of the spikelet, elongate into a leafy morphology.	x	x
TraesCS5A02G265900	*SPL17*	Os	(26)	Panicle branching and spikelets are reduced in *SPL17* RNAi plants.	x	
TraesCS1B02G448200	*NECK LEAF1 (NL1)*	Os	(27)	Rice *nl1* mutant shows delay in flowering time and smaller panicles.	x	
TraesCS7B02G384000	*APO1 / WAPO1*	Os	(28)<br>(29)	Rice *APO1* and its wheat ortholog *WAPO1* positively control spikelet number.	x	
TraesCS5B02G560300	*GA20OX1 / GNP1*	Os	(30)	Natural variants with increased expression are associated with increases in grain number.	x	
**Cluster 9**						
TraesCS4B02G042700	*TB1-1*	Ta	(31)<br>(32)	Increased dosage of TB1 promotes paired spikelet and delays inflorescence growth.<br>TCP family in wheat.	x	
TraesCS5B02G127600	*RICE CENTRODIALIS1*	Os	(33)	*35S::RCN1* shows delay of transition to the reproductive phase and more branched, denser panicle morphology.	x	
TraesCS7A02G229400	*OsMFT1*	Os	(34)	Overexpressing *OsMFT1* delays heading time and increased spikelets and branches per panicle.		
TraesCS2A02G188500	*vrs1*<br>*TaVRS1*	Hv<br>Ta	(35)<br>(36)	Loss-of-function mutations result in conversion of two-rowed barley rudimentary lateral spikelets into fertile spikelets in six-rowed barley.<br>Over-expression of *TaVRS1-2B* reduces SNS.	 x	
**Cluster 10**						
TraesCS7B02G195200	OsMFT1	Os	(34)	Overexpressing *OsMFT1* delays heading time and increases spikelets and branches per panicle.	x	
TraesCS4A02G409200<br>TraesCSU02G202000<br>TraesCS2B02G310700	*RICE CENTRODIALIS2*	Os	(33)	*35S::RCN2* shows delay of transition to the reproductive phase and more branched, denser panicle morphology.	x	
TraesCS5A02G001900<br>TraesCS5B02G002300	*TB1-2*	Ta	(32)	The wheat *TB1* locus (TCP family) is duplicated.		
TraesCS2A02G514000	*WOX4*		(37)	*OsWOX4* is as a key regulator at the early stages of leaf development.		

References Supplemental Table S3

1. **Poursarebani, N. *et al.*** (2015) The genetic basis of composite spike form in barley and 'Miracle-Wheat'. Genetics **201**: 155-165.

2. **Chuck, G., Muszynski, M., Kellogg, E., Hake, S., and Schmidt, R.J.** (2002) The control of spikelet meristem identity by the *branched silkless1* gene in maize. Science **298**: 1238-1241.

3. **Komatsu, M., Chujo, A., Nagato, Y., Shimamoto, K., and Kyozuka, J.** (2003) *FRIZZY PANICLE* is required to prevent the formation of axillary meristems and to establish floral meristem identity in rice spikelets. Development **130**: 3841-3850.

4. **Ren, D.Y., Li, Y.F., Zhao, F.M., Sang, X.C., Shi, J.Q., Wang, N., Guo, S., Ling, Y.H., Zhang, C.W., Yang, Z.L., and He, G.H.** (2013) *MULTI-FLORET SPIKELET1*, which encodes an AP2/ERF protein, determines spikelet meristem fate and sterile lemma identity in rice. Plant Physiol. **162**: 872-884.

5. **Komatsu, M., Maekawa, M., Shimamoto, K., and Kyozuka, J.** (2001) The *LAX1* and *FRIZZY PANICLE 2* genes determine the inflorescence architecture of rice by controlling rachis-branch and spikelet development. Dev. Biol. **231**: 364-373.

6. **Gallavotti, A., Zhao, Q., Kyozuka, J., Meeley, R.B., Ritter, M.K., Doebley, J.F., Pe, M.E., and Schmidt, R.J.** (2004) The role of *barren stalk1* in the architecture of maize. Nature **432**: 630-635.

7. **Si, L.Z.** *et al.* (2016) *OsSPL13* controls grain size in cultivated rice. Nat. Genet. **48**: 447-456.

8. **Youssef, H.M., Eggert, K., Koppolu, R., Alqudah, A.M., Poursarebani, N., Fazeli, A., Sakuma, S., Tagiri, A., Rutten, T., Govind, G., Lundqvist, U., Graner, A., Komatsuda, T., Sreenivasulu, N., and Schnurbusch, T.** (2017) VRS2 regulates hormone-mediated inflorescence patterning in barley. Nat. Genet. **49**: 157-161.

9. **Duan, E.C., Wang, Y.H., Li, X.H., Lin, Q.B., Zhang, T., Wang, Y.P., Zhou, C.L., Zhang, H., Jiang, L., Wang, J.L., Lei, C.L., Zhang, X., Guo, X.P., Wang, H.Y., and Wan, J.M.** (2019) OsSHI1 Regulates Plant Architecture Through Modulating the Transcriptional Activity of IPA1 in Rice. Plant Cell **31**: 1026-1042.

10. **Wang, W.F., Li, G., Zhao, J., Chu, H.W., Lin, W.H., Zhang, D.B., Wang, Z.Y., and Liang, W.Q.** (2014) DWARF TILLER1, a WUSCHEL-related homeobox transcription factor, is required for tiller growth in rice. PLoS Genet. **10**: e1004154

11. **Jeon, J.S., Jang, S., Lee, S., Nam, J., Kim, C., Lee, S.H., Chung, Y.Y., Kim, S.R., Lee, Y.H., Cho, Y.G., and An, G.** (2000) *leafy hull sterile1* is a homeotic mutation in a rice MADS box gene affecting rice flower development. Plant Cell **12**: 871-884.

12. **Wu, D., Liang, W.Q., Zhu, W.W., Chen, M.J., Ferrandiz, C., Burton, R.A., Dreni, L., and Zhang, D.B.** (2018) Loss of LOFSEP transcription factor function converts spikelet to leaf-like structures in rice. Plant Physiol. **176**: 1646-1664.

13. **Cui, R.F., Han, J.K., Zhao, S.Z., Su, K.M., Wu, F., Du, X.Q., Xu, Q.J., Chong, K., Theissen, G., and Meng, Z.** (2010) Functional conservation and diversification of class E floral homeotic genes in rice (*Oryza sativa*). Plant J. **61**: 767-781.

14. **Yao, S.G., Ohmori, S., Kimizu, M., and Yoshida, H.** (2008) Unequal genetic redundancy of rice *PISTILLATA* orthologs, *OsMADS2* and *OsMADS4*, in lodicule and stamen development. Plant Cell Physiol. **49**: 853-857.

15. **Prasad, K., and Vijayraghavan, U.** (2003) Double-stranded RNA interference of a rice *PI/GLO* paralog, *OsMADS2*, uncovers its second-whorl-specific function in floral organ patterning. Genetics **165**: 2301-2305.

16. **Li, H., Liang, W., Jia, R., Yin, C., Zong, J., Kong, H., and Zhang, D.** (2010) The *AGL6*-like gene *OsMADS6* regulates floral organ and meristem identities in rice. Cell Res. **20**: 299-313.

17. **Ohmori, S., Kimizu, M., Sugita, M., Miyao, A., Hirochika, H., Uchida, E., Nagato, Y., and Yoshida, H.** (2009) *MOSAIC FLORAL ORGANS1*, an *AGL6*-like MADS box gene, regulates floral organ identity and meristem fate in rice. Plant Cell **21**: 3008-3025.

18. **Yamaguchi, T., Lee, D.Y., Miyao, A., Hirochika, H., An, G.H., and Hirano, H.Y.** (2006) Functional diversification of the two C-class MADS box genes *OsMADS3* and *OsMADS58* in *Oryza sativa*. Plant Cell **18**, 15-28.

19. **Sang, X., Li, Y., Luo, Z., Ren, D., Fang, L., Wang, N., Zhao, F., Ling, Y., Yang, Z., and Liu, Y., He, G.** (2012) *CHIMERIC FLORAL ORGANS1*, encoding a monocot-specific MADS box protein, regulates floral organ identity in rice. Plant Physiol. **160**: 788-807.

20. **Zhuang, H., Wang, H.L., Zhang, T., Zeng, X.Q., Chen, H., Wang, Z.W., Zhang, J., Zheng, H., Tang, J., Ling, Y.H., Yang, Z.L., He, G.H., and Li, Y.F.** (2020) *NONSTOP GLUMES1* encodes a C2H2 zinc finger protein that regulates spikelet development in rice. Plant Cell **32**: 392-413.

21. **Jost, M., Taketa, S., Mascher, M., Himmelbach, A., Yuo, T., Shahinnia, F., Rutten, T., Druka, A., Schmutzer, T., Steuernagel, B., Beier, S., Taudien, S., Scholz, U., Morgante, M., Waugh, R., and Stein, N.** (2016) A homolog of *Blade-On-Petiole 1* and *2* (*BOP1/2*) controls internode length and homeotic changes of the barley inflorescence. Plant Physiol. **171**: 1113-1127.

22. **Yoshida, A., Sasao, M., Yasuno, N., Takagi, K., Daimon, Y., Chen, R.H., Yamazaki, R., Tokunaga, H., Kitaguchi, Y., Sato, Y., Nagamura, Y., Ushijima, T., Kumamaru, T., Iida, S., Maekawa, M., and Kyozuka, J.** (2013) *TAWAWA1*, a regulator of rice inflorescence architecture, functions through the suppression of meristem phase transition. Proc. Natl. Acad. Sci. U.S.A. **110** : 767-772.

23. **Fujita, D., Trijatmiko, K.R., Tagle, A.G., Sapasap, M.V., Koide, Y., Sasaki, K., Tsakirpaloglou, N., Gannaban, R.B., Nishimura, T., Yanagihara, S., Fukuta, Y., Koshiba, T., Slamet-Loedin, I.H., Ishimaru, and T., Kobayashi, N.** (2013) *NAL1* allele from a rice landrace greatly increases yield in modern indica cultivars. Proc. Natl. Acad. Sci. U.S.A **110**: 20431-20436.

24. **Sakamoto, T., Kobayashi, M., Itoh, H., Tagiri, A., Kayano, T., Tanaka, H., Iwahori, S., and Matsuoka, M.** (2001) Expression of a *Gibberellin 2-oxidase* gene around the shoot apex is related to phase transition in rice. Plant Physiol. **125**: 1508-1516.

25. **Kobayashi, K., Maekawa, M., Miyao, A., Hirochika, H., and Kyozuka, J.** (2010) *PANICLE PHYTOMER2* (*PAP2*), encoding a SEPALLATA subfamily MADS-box protein, positively controls spikelet meristem identity in rice. Plant Cell Physiol. **51**: 47-57.

26. **Wang, L., Sun, S.Y., Jin, J.Y., Fu, D.B., Yang, X.F., Weng, X.Y., Xu, C.G., Li, X.H., Xiao, J.H., and Zhang, Q.F.** (2015) Coordinated regulation of vegetative and reproductive branching in rice. Proc. Natl. Acad. Sci. U.S.A. **112**: 15504-15509.

27. **Wang, L., Yin, H., Qian, Q., Yang, J., Huang, C., Hu, X., and Luo, D.** (2009) *NECK LEAF 1*, a GATA type transcription factor, modulates organogenesis by regulating the expression of multiple regulatory genes during reproductive development in rice. Cell Res. **19**: 598-611.

28. **Ikeda, K., Ito, M., NagasawaO, N., Kyozuka, J., and Nagato, Y.** (2007) Rice *ABERRANT PANICLE ORGANIZATION 1*, encoding an F-box protein, regulates meristem fate. Plant J. **51**: 1030-1040.

29. **Kuzay, S., Xu, Y., Zhang, J., Katz, A., Pearce, S., Su, Z., Fraser, M., Anderson, J.A., Brown-Guedira, G., DeWitt, N., Peters Haugrud, A., Faris, J.D., Akhunov, E., Bai, G., and Dubcovsky, J.** (2019) Identification of a candidate gene for a QTL for spikelet number per spike on wheat chromosome arm 7AL by high-resolution genetic mapping. Theor. Appl. Genet. **132**: 2689–2705.

30. **Wu, Y., Wang, Y., Mi, X.F., Shan, J.X., Li, X.M., Xu, J.L., and Lin, H.X.** (2016) The QTL *GNP1* encodes GA20ox1, which Increases grain number and yield by increasing cytokinin activity in rice panicle meristems. PLoS Genet. **12**: e1006386.

31. **Dixon, L.E., Greenwood, J.R., Bencivenga, S., Zhang, P., Cockram, J., Mellers, G., Ramm, K., Cavanagh, C., Swain, S.M., and Boden, S.A.** (2018) *TEOSINTE BRANCHED1* regulates inflorescence architecture and development in bread wheat (*Triticum aestivum*). Plant Cell **30**: 563-581.

32. **Zhao, J., Zhai, Z., Li, Y., Geng, S., Song, G., Guan, J., Jia, M., Wang, F., Sun, G., Feng, N., Kong, X., Chen, L., Mao, L., and Li, A.** (2018) Genome-wide identification and expression profiling of the *TCP* family genes in spike and grain development of wheat (*Triticum aestivum* L.). Front. Plant Sci. **9**: 1282.

33. **Nakagawa, M., Shimamoto, K., and Kyozuka, J.** (2002) Overexpression of *RCN1* and *RCN2*, rice *TERMINAL FLOWER 1/CENTRORADIALIS* homologs, confers delay of phase transition and altered panicle morphology in rice. Plant J. **29**: 743-750.

34. **Song, S., Wang, G., Hu, Y., Liu, H., Bai, X., Qin, R., and Xing, Y.** (2018) *OsMFT1* increases spikelets per panicle and delays heading date in rice by suppressing *Ehd1*, *FZP* and *SEPALLATA*-like genes. J. Exp. Bot. **69**: 4283-4293.

35. **Komatsuda, T., Pourkheirandish, M., He, C., Azhaguvel, P., Kanamori, H., Perovic, D., Stein, N., Graner, A., Wicker, T., Tagiri, A., Lundqvist, U., Fujimura, T., Matsuoka, M., Matsumoto, T., and Yano, M.** (2007) Six-rowed barley originated from a mutation in a homeodomain-leucine zipper I-class homeobox gene. Proc. Natl. Acad. Sci. U.S.A. **104**: 1424-1429.

36. **Wang, Y.G., Yu, H.P., Tian, C.H., Sajjad, M., Gao, C.C., Tong, Y.P., Wang, X.F., and Jiao, Y.L**. (2017) Transcriptome association identifies regulators of wheat spike architecture. Plant Physiol. **175**: 746-757.

37. **Yasui, Y., Ohmori, Y., Takebayashi, Y., Sakakibara, H., and Hirano, H.Y.** (2018) *WUSCHEL-RELATED HOMEOBOX4* acts as a key regulator in early leaf development in rice. PLoS Genet. **14**: e1007365.

Supplemental Table S4. Primers used for gene cloning, genotyping of mutations and transgenic plants, qRT-PCR, generation of Y2H and Y3H constructs and *in situ* hybridization.

Use & gene	Orientation	Primer sequence 5’ to 3’	Res. Enz.
Gene cloning (into Gateway™ pDONR™/Zeo Vector)			
VRT2	VRT2-ATTB1	ggggacaagtttgtacaaaaaagcaggcttcATGGCGCGGGAGAGGCGGGC	
	VRT2-ATTB2	ggggaccactttgtacaagaaagctgggtcTTACTTCCAAGGTAACGCTAG	
SVP1	SVP1-ATTB1	ggggacaagtttgtacaaaaaagcaggctatATGGCGCGGGAGCGGAGGGAGA	
	SVP1-ATTB2	ggggaccactttgtacaagaaagctgggtaTTACTTCCACGGAAGGCAGG	
SEP1-2	SEP1-2-ATTB1	ggggacaagtttgtacaaaaaagcaggctatATGGGTCGGGGGAAGGTGGAG	
(*OsMADS1*)	SEP1-2-ATTB2	ggggaccactttgtacaagaaagctgggtaTCATATCCAACCTGCAGATG	
SEP1-4	SEP1-4-ATTB1	ggggacaagtttgtacaaaaaagcaggctatATGGGGCGCGGCAAGGTGGAGCT	
(*OsMADS5*)	SEP1-4-ATTB2	ggggaccactttgtacaagaaagctgggttTCATTCTTTGTTCAACTGATCC	
SEP1-6	SEP1-6-ATTB1	ggggacaagtttgtacaaaaaagcaggctatATGGGTCGCGGCAAGGTGGT	
(*OsMADS34*)	SEP1-6-ATTB2	ggggaccactttgtacaacaaagctgggttCTATGCCATCCATGCAGGCGG	
Genotyping of mutants			
vrt-A2	Forward	TGAAGTATATACCTGCCTCGG	CAPS(*Bbv*I)
	Reverse	ACCTTGTGTTGGAGGTCAGTA	
vrt-B2	Forward	GGATTGCAGAGGGTGCTTTGTACGAAG	dCAPS(*Mbo*II)
	Reverse	CAGAGTCTAAACAAACAATTG	
svp-A1	Forward	TCAGCTTGCAGAAGCTAGTCTTCGACGAAG	dCAPS(*Mbo*II)
	Reverse	TTGAAAAATGACATGTTCACTG	
svp-B1	Forward	GTTCATGATAGCTGTAAAAAAAT	dCAPS(*Pvu*II)
	Reverse	AACCTTAGTCGAAGACTAGCTTCTGCCAGCT	
Transgenic *VRT2* plants			
297	Forward	TCCCTGAAACTTGCGTTACC	
1064	Reverse	TCGCTTATTTAAAGGGCGAAT	
qRT-PCR			
VRT2	Forward	GAGGTGAGGAACTTGACGGA	
(*OsMADS55*)	Reverse	GAATTGCCGGTCCTTTGTAC	
TaSVP1 [1]	Forward	CACAGGGTGCTTCAGACAAA	
(*OsMADS22*)	Reverse	TTGGAATCTGGCCTACTTGG	
TaSVP3 [1]	Forward	CCAGCAGATGAGAGGAGAGG	
(*OsMADS47*)	Reverse	GGCTCTTGGTTTTCAGAACG	
TaSEP1-2	MADS1-Fw	GGAGCAAGAATTGCAGGATG	
(*OsMADS1*) [2]	MADS1-Rev	GCTASACTGCCCTCCGTCTT (S= 50% G + 50% C primer mix)	
TaSEP1-4	MADS5-Fw	GGCGACAAAGAGCCAACAGT	
(*OsMADS5*) [2]	MADS5-Rev	TCCAACATCCTGGCAAGACA	
TaSEP3-1	MADS7-Fw	CAGTTGGAGGAGAGCAACCA	
(*OsMADS7*) [2]	MADS7-Rev	AAGGGGGTGGAAGAATCCAT	
TaSEP3-2	MADS8-Fw	CCAACTTGCTCGGCTACGAC	
(*OsMADS8*) [2]	MADS8-Rev	TGCGTTGTTTATCTGCTCCTG	
TaPI-1	MADS4-Fw	AGATGCTGGAGGAGGAGCAC	
(*OsMADS4*) [2]	MADS4-Rev	CGGCATCTGGGAAGTGAAAT	
TaAP3	MADS16Fw3	AAAATGTCGATGCCGCTCTC	
(*OsMADS16*) [2]	MADS16Rev3	CTCCTGGGAGTGCTTCACCT	
TaSEP1-6	MADS34-Fw	GCAGCCAGAGCACTTCTTCC	
(*OsMADS34*) [2]	MADS34-Rev	GGCTGGTTCACATCCATGC	
FUL2 [3]	FUL2-FW	CCATACAAAAATGTCACAAGC	
	FUL2-Rev	TTCTGCCTCTCCACCAGTTC	
VRN1 [4]	VRN1-Ex5-6-F2	AAGAAGGAGAGGTCACTGCAGG	
	VRN1-Ex8-R37	GGCTGCACTGCCGCA	
ACTIN	Actin F	ACCTTCAGTTGCCCAGCAAT	
	Actin R	CAGAGTCGAGCACAATACCAGTTG	

Continuation of Supplemental Table S4

Gene	Probe name	Primer sequence 5' to 3'
Y2H and Y3H constructs [5]		
VRN1	VRN1-NdeI-F	CTGCATATGGGGCGGGGGAAGGTGCAG
	VRN1-EcoRI-R	GAATTCCCCGTTGATGTGGCTCACC
FUL2	FUL2-EcoRI-F	GAATTCATGGGTCGCGGCAAGGTGCAG
	FUL2-BamHI-R	GGGATCCGCGTTGAGGTGGCTCAGCATC
FUL3	FUL3-NdeI-F	CTGCATATGGGGCGCGGGCCGGTGCAG
	FUL3-EcoRI-R	GAATTCTCTGTTGCTGATGGTGGAGAG
VRT2	VRT2-NdeI-F	CCCCATATGGCGCGGGAGAGGCGGGC
	VRT2-EcoRI-R	CCCGAATTCCTTCCAAGGTAACGCTAG
SVP1	SVP1-NedI-F	CCCCATATGGCGCGGGAGCGGAGG
	SVP1-EcoRI-R	CCCGAATTCCTTCCACGGGAGGCAGGGCA
SVP3	SVP3-NdeI-F	AATCATATGGCGGGGAAGAGGGAGAGG
	SVP3-EcoRI-R	AAGAATTCCTTCGAGTTGTAGAGTGGTAATC
SEP1-2	SEP1-2-EcoRI-F	TGTATCGCCGGAATTCATGGGTCGGGGGAAG
	SEP1-2-BamHI-R	GCAGGTCGACGGATCCTCATATCCAACCTGCAG
SEP1-4	SEP1-4-EcoRI-F	TGTATCGCCGGAATTCATGGGGCGCGGCAAGG
	SEP1-4-BamHI-R	GCAGGTCGACGGATCCTCATTCTTTGTTCAACT
SEP1-6	SEP1-6-EcoRI-F	TGTATCGCCGGAATTCATGGGTCGCGGCAAGGTG
	SEP1-6-BamHI-R	GCAGGTCGACGGATCCCTATGCCATCCATGCAGG
VRT2	VRT2-M25-NotI-F	GAAAGGTGGCGGCCGCATGGCGCGGGAGAGGCGG
	VRT2-M25-BglII-R	ATCAGCCCGAAGATCTTTACTTCCAAGGTAACGC

Use & gene	Orientation	Primer sequence 5' to 3'	Size	Source
In situ hybridization				
VRT2	VRT2_T3_609F	**ATTAACCCTCACTAAAGGGA**ACATTCGGGAAGCTCACAGG	409 bp	*T. monococcum*
	VRT2_T7_1017R	**TAATACGACTCACTATAGGG**TTTGCCCTGTCTCAGCGAAT		
SVP1	SVP1_T3_391F	**ATTAACCCTCACTAAAGGGA**GAAACTGGTCTGCACAGGGT	412 bp	*T. monococcum*
	SVP1_T7_801R	**TAATACGACTCACTATAGGG**TAACTGATCTCGCTTCCGCC		
FUL2	ful2_T3_708F2	**ATTAACCCTCACTAAAGGGA**CTACCCGCCAGTGACGATG	370 bp	*T. monococcum*
	ful2_T7_1077R2	**TAATACGACTCACTATAGGG**GCCAAGCAAGTCATCCATGC		
VRN1	vrn1_T3_627F2	**ATTAACCCTCACTAAAGGGA**CATTCATCCAGCGGCAACAG	320 bp	*T. monococcum*
	vrn1_T7_945R2	**TAATACGACTCACTATAGGG**ACTCGTACAGCCATCTCAGC		
CEN2	CEN2_T3_F	**ATTAACCCTCACTAAAGGGA**TCACAGGTTCACCTTCGTGC	378 bp	*T. monococcum*
	CEN2_T7_R	**TAATACGACTCACTATAGGG**TGCACGTACACTGGTTCACA		
VRT2	VRT2_ 609F	ACATTCGGGAAGCTCACAGG	409 bp	Kronos
	VRT2_ 1017R	TTTGCCCTGTCTCAGCGAAT		
SVP1	SVP1_391F	GAAACTGGTCTGCACAGGGT	412 bp	Kronos
	SVP1_801R	TAACTGATCTCGCTTCCGCC		
CEN2	CEN2_Fw	GGAAGGGAGGTGGTGAGCTA	502 bp	Kronos
	CEN2_Rev	AACCTTCGTCTCCACAAATAG		

[1] (Li et al., 2019), [2] (Debernardi et al., 2020), [3] (Chen and Dubcovsky, 2012), and [4] (Yan et al., 2006).

[5] Restriction sites for enzyme-based cloning and overlapping vector sequences required for in-fusion cloning are underlined.

References Supplemental Table S4

Chen, A., and Dubcovsky, J. (2012). Wheat TILLING mutants show that the vernalization gene *VRN1* down-regulates the flowering repressor *VRN2* in leaves but is not essential for flowering. PLoS Genet. **8**: e1003134.

Debernardi, J.M., Greenwood, J.R., Jean Finnegan, E., Jernstedt, and J., Dubcovsky, J. (2020). *APETALA 2*-like genes *AP2L2* and *Q* specify lemma identity and axillary floral meristem development in wheat. Plant J. **101**: 171-187.

Li, C.X., Lin, H.Q., Chen, A., Lau, M., Jernstedt, J., and Dubcovsky, J. (2019). Wheat *VRN1*, *FUL2* and *FUL3* play critical and redundant roles in spikelet development and spike determinacy. Development. **146**: dev175398.

Yan, L., Fu, D., Li, C., Blechl, A., Tranquilli, G., Bonafede, M., Sanchez, A., Valarik, M., Yasuda, S., and Dubcovsky, J. (2006). The wheat and barley vernalization gene *VRN3* is an orthologue of *FT*. Proc. Natl. Acad. Sci. U.S.A. **103**: 19581-19586.

Supplemental Table S5. Effects of *vrt2* and *svp1* mutations on heading time, spikelet number per spike, stem length, and leaf number in growth chambers under LD conditions. Experiment 1 compared *vrt-A2* (n = 8), *vrt-B2* (n = 6), *vrt2* (n = 8) and wild type sister lines (n = 4, Supplemental Figure 3). Experiment 2 compared *svp-A1* (n = 6), *svp-B1* (n = 4), *svp1* (n = 15) and wild type sister lines (n = 5). Experiment 3 compared *svp1* (n = 9), *vrt2* (n = 9), *vrt2 svp1* (n = 13) and wild type sister lines (n = 9, Supplemental Figure 3). In Experiments 1 and 2 main effects are the differences between least square means for the mutant allele minus the WT allele (*P* values for main effects and interactions are from the 2 x 2 factorial ANOVA). The third row is the difference between the combined mutant and the WT. Experiment 3 corresponds to Figure 3 in the main manuscript. Significant differences between the mutants and the WT are in blue if the mutant increases the value of the trait relative to the WT and in red if the mutants decreased the values (*P* values are from the Dunnett tests). Stem nodes are numbered from the peduncle (-1 is the closest internode to the peduncle and -4 the most basal node). ns= not significant, * = $P < 0.05$, ** = $P < 0.01$, *** = $P < 0.001$. Statistical analyses are presented in Supplemental Data Set 2,

Exp.	Effect	HD days	SNS	Peduncle cm	-1 inter-node cm	-2 inter-node cm	-3 inter-node cm	-4 inter-node cm	Leaf No.
1	*VRT-A2* Main	1.9 ***	3.4 ***	-11.1 ***	2.1 ***	0.2 ns	-0.1 ns	-0.2 ns	NA
1	*VRT-B2* Main	1.5 **	1.5 ***	-11.1 ***	0.3 ns	-0.5 *	-0.3 ns	-0.3 ns	NA
1	Interaction	*	*	***	***	***	ns	ns	NA
1	*vrt2* – WT	3.4 ***	4.9 ***	-22.1 ***	2.4 ***	-0.3 ns	-0.4 ns	-0.5 ns	NA
2	*SVP-A1* Main	1.2 ns	1.7 **	-1.8 ns	1.1 *	0.1 ns	-0.2 ns	-0.9 ns	0.1 ns
2	*SVP-B1* Main	1.3 ns	2.5 ***	-7.7 ***	1.3 *	0.7 *	-0.1 ns	-1.3 *	0.2 ns
2	Interaction	ns	ns	*	ns	ns	ns	ns	ns
2	*svp1* - WT	2.9 ns	4.1 ***	-9.5 ***	2.4 ***	0.8 ns	-0.3 ns	-2.2 **	0.3 ns
3	*vrt2* - WT	3.1 *	3.6 ***	-20.1 ***	1.9 *	0.1 ns	0.2 ns	0.5 ns	0.7 ns
3	*svp1* - WT	4.8 ***	2.1 *	-8.5 *	-0.6 ns	-0.2 ns	-0.7 ns	-0.7 ns	0.6 ns
3	*vrt2 svp1* - WT	29.0 ***	13.0 ***	-25.0 ***	-4.0 ***	-0.8 ns	-1.5 *	0.1 ns	4.4 ***

Supplemental Table S6. Summary of Y2H interactions (lower triangular table) and BiFC interactions (upper triangular table) tested among the SQUAMOSA, SVP and SEP MADS-box proteins.

	VRN1	FUL2	FUL3	VRT2	SVP1	SVP3	SEP1-2	SEP1-4	SEP1-6
VRN1	Y2H-	NA	NA	BiFC+[1]	BiFC+	NA	BiFC+	BiFC-	(BiFC-)[2]
FUL2	Y2H+	Y2H+	NA	BiFC+	BiFC+	NA	BiFC+	(BiFC-)[2]	(BiFC-)[2]
FUL3	Y2H-	Y2H+	Y2H+[3]	BiFC+	BiFC+	NA	BiFC+	BiFC-	BiFC-
VRT2	Y2H+	Y2H+[2]	Y2H+	Y2H-	NA	NA	NA	NA	NA
SVP1	Y2H+	Y2H+	Y2H+	Y2H-	Y2H+	NA	NA	NA	NA
SVP3	Y2H-	Y2H+	Y2H-	Y2H-	Y2H-	Y2H-	NA	NA	NA
SEP1-2	Y2H+	Y2H+	Y2H+	Y2H-	Y2H+	Y2H-	NA	NA	NA
SEP1-4	Y2H+	Y2H+	Y2H+	Y2H-	Y2H-	Y2H-	NA	NA	NA
SEP1-6	Y2H+	Y2H+	Y2H+	Y2H-	Y2H-	Y2H+[3]	NA	NA	NA

[1] The interaction VRT2-VRN1 was confirmed by CoIP and luciferase assays in previous study (Xie et al., 2019).

[2] (BiFC-) in parentheses indicates no clear nuclear signal but bright fluorescent aggregates outside the nucleus.

[3] Weak interaction.

Reference Supplemental Table S6

Xie, L., Zhang, Y., Wang, K., Luo, X., Xu, D., Tian, X., Li, L., Ye, X., Xia, X., Li, W., Yan, L., and Cao, S. (2019). *TaVrt2*, an SVP-like gene, cooperates with *TaVrn1* to regulate vernalization-induced flowering in wheat. *New Phytol*. doi: 10.1111/nph.16339
