# Supplementary material for: Interactions between SQUAMOSA and SHORT VEGETATIVE PHASE MADS-box proteins regulate meristem transitions during wheat spike development
Source: Plant Cell. 2021 Nov 2;33(12):3621–44. doi: 10.1093/plcell/koab243 (PMC8643710; doi:10.1093/plcell/koab243)
Supplement: koab243_Supplementary_Data [file koab243_supplementary_data.zip › tpc.21.00586_SupplementalDataSet2.docx]

**Supplemental Data Set 2. Statistical analysis tables**

**Supplemental Statistical Analyses for Figure 3**

2 x 2 Factorial ANOVA *VRT2* x *SVP1* (WT and mutant alleles). Effects of individual and combined *vrt2* and *svp1* mutants on important agronomic traits.

**Figure 3C.** **Days to heading**. Power transformation (^0.01) to restore normality of residuals and homogeneity of variances. ANOVA *P* values are the same in the transformed and untransformed data.

Sum of

Source DF Squares Mean Square F Value Pr > F

Genotype 3 0.0001710 0.0000570 265.51 <0.0001

Error 36 0.0000077 0.0000002

Corrected Total 39 0.0001787

R-Square: 0.956758

DF SS Mean Square F Value Pr > F

vrt2 1 0.0000508 0.0000508 205.48 <0.0001

svp1 1 0.0000668 0.0000668 258.72 <0.0001

Interaction vrt2 x svp1 1 0.0000260 0.0000260 122.49 <0.0001

Dunnett’s Test for Days to heading. *P* values are from the transformed data but differences between means are untransformed to facilitate visualization.

Genotype Difference

Comparison Between Means *P* value

vrt2svp1 - WT 28.966 <0.001

vrt2 - WT 4.778 <0.001

svp1 - WT 3.111 <0.05

**Figure 3D**. **Leaf number.**

Sum of

Source DF Squares Mean Square F Value Pr > F

Genotype 3 87.35 29.12 94.42 <0.0001

Error 26 8.02 0.31

Corrected Total 29 95.37

R-Square: 0.915926

DF SS Mean Square F Value Pr > F

vrt2 1 37.50 37.50 121.61 <0.0001

svp1 1 35.15 35.15 113.98 <0.0001

Interaction vrt2 x svp1 1 17.81 17.81 57.77 <0.0001

Dunnett’s Test for Leaf number.

Genotype Difference

Comparison Between Means *P* value

vrt2svp1 - WT 4.4107 <0.001

vrt2 - WT 0.6964 ns

svp1 - WT 0.6250 ns

**Figure 3E**. **Spikelet number per spike (SNS)**

Sum of

Source DF Squares Mean Square F Value Pr > F

Genotype 3 1097.99 366.00 135.68 <0.0001

Error 35 97.11 2.70

Corrected Total 39 1195.10

R-Square: 0.918742

DF SS Mean Square F Value Pr > F

vrt2 1 493.04 493.04 182.77 <0.0001

svp1 1 313.08 313.08 116.06 <0.0001

Interaction vrt2 x svp1 1 123.26 123.26 45.69 <0.0001

Dunnett’s Test for s**pikelet number per spike**.

Genotype Difference

Comparison Between Means *P* value

vrt2svp1 - WT 13.0278 <0.001

vrt2 - WT 3.5556 <0.001

svp1 - WT 2.1111 <0.05

**Figure 3F**. **Plant height.**

Sum of

Source DF Squares Mean Square F Value Pr > F

Genotype 3 5897.36 1965.79 195.01 <0.0001

Error 36 362.90 10.08

Corrected Total 39 6260.26

R-Square: 0.942032

DF SS Mean Square F Value Pr > F

vrt2 1 4193.32 4193.33 415.98 <0.0001

svp1 1 971.67 971.67 96.39 <0.0001

Interaction vrt2 x svp1 1 100.86 100.86 10.01 0.0032

Dunnett’s Test for plant height.

Genotype Difference

Comparison Between Means *P* value

vrt2svp1 - WT -30.721 <0.001

vrt2 - WT -17.522 <0.001

svp1 - WT -6.767 <0.001

**Supplemental Statistical Analyses for Figure 6**

Phenotypic characterization of Kronos lines constitutively expressing *VRT2*. One-way ANOVA including WT and three independent *UBI_pro_:VRT2* transgenic events (T#2= strongest, T#4 = intermediate and T#8 weakest phenotypic effects).

**Figure 6A.** **Days to heading**.

Sum of

Source DF Squares Mean Square F Value Pr > F

Genotype 3 967.473 322.491 86.72 <0.0001

Error 52 193.367 3.719

Corrected Total 55 1160.839

R-Square: 0.8334

Dunnett’s Test for days to heading.

Genotype Difference

Comparison Between Means *P* value

T#2 - WT 10.667 <0.001

T#4 - WT 4.283 <0.001

T#8 - WT 1.433 ns

**Figure 6B.** **Stem length**.

Sum of

Source DF Squares Mean Square F Value Pr > F

Genotype 3 2394.607 798.202 125.12 <0.0001

Error 52 331.732 6.379

Corrected Total 55 2726.339

R-Square: 0.8783

Dunnett’s Test for stem length.

Genotype Difference

Comparison Between Means *P* value

T#2 - WT -11.793 <0.001

T#4 - WT -12.331 <0.001

T#8 - WT 2.557 <0.05

**Figure 6C.** **Spikelet number per spike (SNS)**. Power transformation (^-3) to restore normality of residuals and homogeneity of variances. ANOVA *P* values are the same in the transformed and untransformed data.

Sum of

Source DF Squares Mean Square F Value Pr > F

Genotype 3 1.82E-8 6.08E-9 63.29 <0.0001

Error 52 4.99E-9 9.60E-11

Corrected Total 55 2.32E-8

R-Square: 0.7850

Dunnett’s Test for spikelet number per spike. *P* values are from the transformed data but differences between means are untransformed to facilitate visualization.

Genotype Difference

Comparison Between Means *P* value

T#2 - WT 3.733 <0.001

T#4 - WT 2.854 <0.001

T#8 - WT -1.033 <0.01

**Figure 6D.** **Spikelet density** (SNS / spike length).

Sum of

Source DF Squares Mean Square F Value Pr > F

Genotype 3 10.549 3.516 101.87 <0.0001

Error 52 1.795 0.035

Corrected Total 55 12.343

R-Square: 0.8546

Dunnett’s Test for spikelet density.

Genotype Difference

Comparison Between Means *P* value

T#2 - WT 1.025 <0.001 T#4 - WT 0.461 <0.001

T#8 - WT -0.064 ns

**Figure 6E.** **Length of glume 1**. Power transformation (^-0.5) to restore normality of residuals and homogeneity of variances. ANOVA *P* values are the same in the transformed and untransformed data.

Sum of

Source DF Squares Mean Square F Value Pr > F

Genotype 3 1.459 0.486 282.07 <0.0001

Error 52 0.090 0.002

Corrected Total 55 1.549

R-Square: 0.9421

Dunnett’s Test for length of glume 1. *P* values are from the transformed data but differences between means are untransformed to facilitate visualization.

Genotype Difference

Comparison Between Means *P* value

T#2 - WT 2.867 <0.001

T#4 - WT 0.859 <0.001

T#8 - WT 0.067 ns

**Figure 6F.** **Length of lemma 1**. Power transformation (^-0.5) to restore normality of residuals and homogeneity of variances. ANOVA *P* values are the same in the transformed and untransformed data.

Sum of

Source DF Squares Mean Square F Value Pr > F

Genotype 3 1.980 0.660 401.67 <0.0001

Error 52 0.085 0.001

Corrected Total 55 2.065

R-Square: 0.9586

Dunnett’s Test for Length of lemma1. *P* values are from the transformed data but differences between means are untransformed to facilitate visualization.

Genotype Difference

Comparison Between Means *P* value

T#2 - WT 4.813 <0.001

T#4 - WT 0.818 <0.001

T#8 - WT 0.037 ns

**Supplemental Statistical Analyses for Figure 8**

One-way ANOVA for the expression of wheat flowering genes in developing spikes at the TS stage of *UBI_pro_:VRT2* transgenic lines T#8, T#4 and T#2 and sister lines without the transgene (WT) relative to *ACTIN* as endogenous control.

**Figure 8A.** ***VRT2***. Power transformation (^0.5) to improve normality of residuals and homogeneity of variances. ANOVA *P* values are the same in the transformed and untransformed data.

Sum of

Source DF Squares Mean Square F Value Pr > F

Genotype 3 73.393 24.464 182.74 <0.0001

Error 12 1.607 0.134

Corrected Total 15 75.000

R-Square: 0.9786

Dunnett’s Test for *VRT2*. *P* values are from the transformed data but differences between means are untransformed to facilitate visualization.

Genotype Difference

Comparison Between Means *P* value

T#2 - WT 33.478 <0.001

T#4 - WT 33.851 <0.001

T#8 - WT 4.581 <0.001

**Figure 8B (left). *VRN1*.**

Sum of

Source DF Squares Mean Square F Value Pr > F

Genotype 3 0.243 0.081 3.51 0.0493

Error 12 0.277 0.023

Corrected Total 15 0.521

R-Square: 0.4673

Dunnett’s Test for *VRN1*.

Genotype Difference

Comparison Between Means *P* value

T#2 - WT -0.348 <0.05 T#4 - WT -0.191 ns

T#8 - WT -0.195 ns

**Figure 8B (right).** ***FUL2*.**

Sum of

Source DF Squares Mean Square F Value Pr > F

Genotype 3 0.135 0.045 1.30 0.3183

Error 12 0.413 0.034

Corrected Total 15 0.547

R-Square: 0.2458

Dunnett’s Test for *FUL2*.

Genotype Difference

Comparison Between Means *P* value

T#2 - WT -0.199 ns T#4 - WT -0.102 ns

T#8 - WT 0.036 ns

**Figure 8C (left).** ***PI1*.**

Sum of

Source DF Squares Mean Square F Value Pr > F

Genotype 3 0.382 0.127 3.92 0.0366

Error 12 0.390 0.032

Corrected Total 15 0.772

R-Square: 0.4951

Dunnett’s Test for *PI1*.

Genotype Difference

Comparison Between Means *P* value

T#2 - WT -0.412 <0.05

T#4 - WT -0.184 ns

T#8 - WT -0.082 ns

**Figure 8C (right).** *AP3-1***.**

Sum of

Source DF Squares Mean Square F Value Pr > F

Genotype 3 0.450 0.150 3.66 0.0440

Error 12 0.491 0.041

Corrected Total 15 0.941

R-Square: 0.4781

Dunnett’s Test for *AP3-1*.

Genotype Difference

Comparison Between Means *P* value

T#2 - WT -0.378 ns

T#4 - WT -0.041 ns

T#8 - WT 0.048 ns

**Figure 8D (left). *AG1*.** Power transformation (^2) to improve normality of residuals and homogeneity of variances. ANOVA *P* values are the same in the transformed and untransformed data.

Sum of

Source DF Squares Mean Square F Value Pr > F

Genotype 3 0.573 0.191 4.53 0.0242

Error 12 0.506 0.042

Corrected Total 15 1.079

R-Square: 0.5308

Dunnett’s Test for *AG1*. *P* values are from the transformed data but differences between means are untransformed to facilitate visualization.

Genotype Difference

Comparison Between Means *P* value

T#2 - WT -0.297 <0.05

T#4 - WT -0.011 ns

T#8 - WT -0.081 ns

**Figure 8D (right). *AG2*.**

Sum of

Source DF Squares Mean Square F Value Pr > F

Genotype 3 0.041 0.014 0.42 0.7427

Error 12 0.039 0.033

Corrected Total 15 0.434

R-Square: 0.0948

Dunnett’s Test for *AG2*.

Genotype Difference

Comparison Between Means *P* value

T#2 - WT -0.074 ns

T#4 - WT -0.029 ns

T#8 - WT -0.135 ns

**Figure 8E (1^st^ from left). *SEP1-2*.**

Sum of

Source DF Squares Mean Square F Value Pr > F

Genotype 3 0.562 0.187 9.82 0.0015

Error 12 0.229 0.019

Corrected Total 15 0.791

R-Square: 0.7106

Dunnett’s Test for *SEP1-2*.

Genotype Difference

Comparison Between Means *P* value

T#2 - WT -0.449 <0.01

T#4 - WT -0.233 ns

T#8 - WT 0.002 ns

**Figure 8E (2^nd^ from left). *SEP1-4*.**

Sum of

Source DF Squares Mean Square F Value Pr > F

Genotype 3 0.498 0.166 8.93 0.0022

Error 12 0.223 0.019

Corrected Total 15 0.721

R-Square: 0.6908

Dunnett’s Test for *SEP1-4*.

Genotype Difference

Comparison Between Means *P* value

T#2 - WT -0.476 <0.001

T#4 - WT -0.309 <0.05

T#8 - WT -0.159 ns

**Figure 8E (3^rd^ from left). *SEP1-6*.**

Sum of

Source DF Squares Mean Square F Value Pr > F

Genotype 3 0.093 0.031 1.37 0.2978

Error 12 0.271 0.022

Corrected Total 15 0.364

R-Square: 0.2557

Dunnett’s Test for *SEP1-6*.

Genotype Difference

Comparison Between Means *P* value

T#2 - WT 0.144 ns

T#4 - WT 0.181 ns

T#8 - WT 0.026 ns

**Figure 8E (4^th^ from left). *SEP3-1*.**

Sum of

Source DF Squares Mean Square F Value Pr > F

Genotype 3 0.793 0.264 7.82 0.0037

Error 12 0.406 0.034

Corrected Total 15 1.199

R-Square: 0.6616

Dunnett’s Test for *SEP3-1*.

Genotype Difference

Comparison Between Means *P* value

T#2 - WT -0.533 <0.01

T#4 - WT -0.263 ns

T#8 - WT 0.009 ns

**Figure 8E (5^th^ from left). *SEP3-2*.**

Sum of

Source DF Squares Mean Square F Value Pr > F

Genotype 3 0.443 0.148 5.19 0.0158

Error 12 0.342 0.028

Corrected Total 15 0.785

R-Square: 0.5648

Dunnett’s Test for *SEP3-2*.

Genotype Difference

Comparison Between Means *P* value

T#2 - WT -0.392 <0.05

T#4 - WT -0.056 ns

T#8 - WT 0.018 ns

**Supplemental Statistical Analyses for Figure 9**

Effect of combined *ful2* mutation and *UBI_pro_:VRT2* T#8 transgenic line on stem elongation and spike / spikelet development. 2 x 2 Factorial ANOVA *FUL2* (WT and mutant alleles) x *UBI_pro_:VRT2* T#8 (WT and transgenic line).

**Figure 9A. Stem length**

Sum of

Source DF Squares Mean Square F Value Pr > F

Genotype 3 829.03 276.34 55.17 <0.0001

Error 20 100.17 5.01

Corrected Total 23 929.20

R-Square: 0.892

DF SS Mean Square F Value Pr > F

T#8 1 3.36 3.36 0.67 0.4224

ful2 1 793.00 793.00 158.33 <0.0001

Int. T#8 x ful2 1 15.99 15.99 3.19 0.0892

Dunnett’s Test for Stem length.

Genotype Difference

Comparison Between Means *P* value

T#8 - WT -0.938 ns

*ful2* - WT -13.925 <0.001

T#8 *ful2* - WT -11.400 <0.001

**Figure 9B. Spikelet number per spike (SNS)**

Sum of

Source DF Squares Mean Square F Value Pr > F

Genotype 3 189.77 63.26 159.51 <0.0001

Error 22 8.72 0.40

Corrected Total 25 198.50

R-Square: 0.956

DF SS Mean Square F Value Pr > F

T#8 1 76.04 76.04 191.74 <0.0001

ful2 1 74.01 74.01 186.61 <0.0001

Int. T#8 x ful2 1 11.01 11.01 27.76 <0.0001

Dunnett’s Test for spikelet number per spike.

Genotype Difference

Comparison Between Means *P* value

T#8 - WT 2.300 <0.001

*ful2* - WT 2.250 <0.001

T#8 *ful2* - WT 7.3750 <0.001

**Figure 9C. Glume 1 length.** Power transformation (^0.01) to restore normality of residuals and homogeneity of variances. ANOVA *P* values are very similar in the transformed and untransformed data.

Sum of

Source DF Squares Mean Square F Value Pr > F

Genotype 3 0.0144 0.00481 65.62 <0.0001

Error 23 0.0017 0.00007

Corrected Total 26 0.0161

R-Square: 0.8954

DF SS Mean Square F Value Pr > F

T#8 1 0.0091 0.0091 124.22 <0.0001

ful2 1 0.0038 0.0038 51.90 <0.0001

Int. T#8 x ful2 1 0.0008 0.0008 10.91 0.0031

Dunnett’s Test for glume 1 length. *P* values are from the transformed data but differences between means are untransformed to facilitate visualization.

Genotype Difference

Comparison Between Means *P* value

T#8 - WT 0.320 <0.001

*ful2* - WT 0.148 ns

T#8 *ful2* - WT 0.888 <0.001

**Figure 9D. Lemma 1 length.**

Sum of

Source DF Squares Mean Square F Value Pr > F

Genotype 3 2.97 0.99 61.02 <0.0001

Error 23 0.37 0.02

Corrected Total 26 3.34

R-Square: 0.8884

DF SS Mean Square F Value Pr > F

T#8 1 1.24 1.24 76.26 <0.0001

ful2 1 0.92 0.92 56.45 <0.0001

Int. T#8 x ful2 1 0.41 0.41 25.06 <0.0001

Dunnett’s Test for lemma 1 length.

Genotype Difference

Comparison Between Means *P* value

T#8 - WT 0.195 <0.05

*ful2* - WT 0.131 ns

T#8 *ful2* - WT 0.850 <0.001

**Supplemental Statistical Analyses for Figure 12**

Paired *t-*Tests for yeast-three-hybrid assays used to test the effect of VRT2 as a competitor. SEPALLATA proteins were expressed as DNA-binding domain fusions, and SQUAMOSA proteins as activation domain fusions

**Figure 12 A. SEP1-2-DB with VRT2 as competitor**

|  | VRN1 | |  | FUL2 | |  | FUL3 | |
| --- | --- | --- | --- | --- | --- | --- | --- | --- |
| Replication | VRT2- | VRT2+ |  | VRT2- | VRT2+ |  | VRT2- | VRT2+ |
| 1 | 0.0037 | 0.0096 |  | 15.8135 | 14.5215 |  | 0.2464 | 0.0108 |
| 2 | 0.0147 | 0.0128 |  | 16.7424 | 15.0430 |  | 0.2530 | 0.0181 |
| 3 | 0.0074 | 0.0160 |  | 16.6806 | 18.8374 |  | 0.2300 | 0.0145 |
| 4 | +0.0110 | 0.0128 |  | 18.9981 | 18.4437 |  | 0.2333 | 0.0108 |
| 5 | 0.0074 | 0.0115 |  | 15.4072 | 13.7331 |  | 0.2497 | 0.0217 |
| 6 | 0.0074 | 0.0153 |  | 15.2842 | 13.2218 |  | 0.2530 | 0.0157 |
| 7 | 0.0111 | 0.0077 |  | 15.6427 | 13.4876 |  | 0.2851 | 0.0126 |
| 8 | 0.0148 | 0.0192 |  | 15.5661 | 13.7399 |  | 0.2624 | 0.0188 |
| 9 | 0.0156 | 0.0110 |  | 16.0393 | 13.4381 |  | 0.2657 | 0.0157 |
| 10 | 0.0094 | 0.0184 |  | 16.6986 | 13.5352 |  | 0.2624 | 0.0157 |
| 11 | 0.0156 | 0.0147 |  | 17.2791 | 13.6467 |  | 0.2754 | 0.0220 |
| 12 | 0.0125 | 0.0184 |  | 18.8729 | 13.5528 |  | 0.2657 | . |
| Mean | 0.0109 | 0.0139 |  | 16.5854 | 14.6001 |  | 0.2568 | 0.0160 |
| SE | 0.0011 | 0.0011 |  | 0.3639 | 0.5644 |  | 0.0046 | 0.0012 |
| Paired tTest | 0.05 | |  | 2.8E-03 | |  | 2.3E-13 | |

**Figure 12 B. SEP1-4-DB with VRT2 as competitor**

|  | VRN1 | |  | FUL2 | |  | FUL3 | |
| --- | --- | --- | --- | --- | --- | --- | --- | --- |
| Rep | VRT2 - | VRT2+ |  | VRT2 - | VRT2+ |  | VRT2 - | VRT2+ |
| 1 | 0.0033 | 0.0042 |  | 2.0216 | 1.5038 |  | 0.0065 | 0.0121 |
| 2 | 0.0043 | 0.0065 |  | 2.0932 | 1.5072 |  | 0.0033 | 0.0151 |
| 3 | 0.0046 | 0.0033 |  | 2.0898 | 1.5279 |  | 0.0163 | 0.0091 |
| 4 | 0.0066 | 0.0065 |  | 2.1068 | 1.5416 |  | 0.0072 | 0.0103 |
| 5 | 0.0082 | 0.0098 |  | 2.1068 | 1.5244 |  | 0.0104 | 0.0121 |
| 6 | 0.0072 | 0.0196 |  | 2.0352 | 1.5348 |  | 0.0098 | 0.0181 |
| 7 | 0.0131 | 0.0090 |  | 2.0342 | 1.6877 |  | 0.0098 | 0.0060 |
| 8 | 0.0059 | 0.0101 |  | 2.0175 | 1.6946 |  | 0.0131 | 0.0150 |
| 9 | 0.0066 | 0.0117 |  | 2.0208 | 1.7463 |  | 0.0066 | 0.0030 |
| 10 | 0.0230 | 0.0201 |  | 2.0542 | 1.7704 |  | 0.0033 | 0.0090 |
| 11 | 0.0164 | 0.0101 |  | 2.0008 | 1.7600 |  | 0.0125 | 0.0060 |
| 12 | 0.0059 | 0.0034 |  | 2.0609 | 1.7669 |  | 0.0115 | 0.0120 |
| Mean | 0.0087 | 0.0095 |  | 2.0535 | 1.6305 |  | 0.0092 | 0.0106 |
| SE | 0.0017 | 0.0016 |  | 0.0108 | 0.0332 |  | 0.0011 | 0.0013 |
| Paired tTest | 0.5993 | |  | 4.5E-07 | |  | 0.4127 | |

**Figure 12 C. SEP1-6-DB with VRT2 as competitor**

|  | VRN1 | |  | FUL2 | |  | FUL3 | |
| --- | --- | --- | --- | --- | --- | --- | --- | --- |
| Rep. | VRT2- | VRT2+ |  | VRT2- | VRT2+ |  | VRT2- | VRT2+ |
| 1 | 0.0105 | 0.0176 |  | 5.8037 | 4.3918 |  | 0.0093 | 0.0188 |
| 2 | 0.0279 | 0.0141 |  | 5.8572 | 4.3361 |  | 0.0186 | 0.0156 |
| 3 | 0.0070 | 0.0106 |  | 6.0286 | 4.6856 |  | 0.0155 | 0.0125 |
| 4 | 0.0105 | 0.0035 |  | 5.8858 | 4.5557 |  | 0.0217 | 0.0094 |
| 5 | 0.0175 | 0.0247 |  | 5.8394 | 4.5371 |  | 0.0062 | 0.0078 |
| 6 | 0.0210 | 0.0070 |  | 5.7430 | 4.7567 |  | 0.0279 | 0.0063 |
| 7 | 0.0053 | 0.0179 |  | 5.8022 | 4.5294 |  | 0.0120 | 0.0089 |
| 8 | 0.0071 | 0.0071 |  | 5.5252 | 4.2072 |  | 0.0181 | 0.0238 |
| 9 | 0.0213 | 0.0107 |  | 5.6151 | 4.3367 |  | 0.0151 | 0.0209 |
| 10 | 0.0142 | 0.0071 |  | 5.5863 | 4.2767 |  | 0.0090 | 0.0238 |
| 11 | 0.0178 | 0.0214 |  | 5.5791 | 4.5483 |  | 0.0211 | 0.0089 |
| 12 | 0.0036 | 0.0107 |  | 5.4604 | 4.3936 |  | 0.0271 | 0.0268 |
| Mean | 0.0136 | 0.0127 |  | 5.7272 | 4.4629 |  | 0.0168 | 0.0153 |
| SE | 0.0022 | 0.0019 |  | 0.0495 | 0.0480 |  | 0.0020 | 0.0021 |
| Paired tTest | 0.7330 | |  | 1.6E-11 | |  | 0.6211 | |

**Supplemental Statistical Analyses for Supplemental Figure 3**

2 x 2 Factorial ANOVA with *VRT2* or *SVP1* homeologs as factors and WT and mutant alleles as levels.

**Supplemental Figure 3A.** ***VRT-A2* x *VRT-B2.* Days to heading (growth chamber).**

Sum of

Source DF Squares Mean Square F Value Pr > F

Genotype 3 46.10 15.37 13.94 <0.0001

Error 22 24.25 1.10

Corrected Total 25 70.35

R-Square: 0.655276

DF SS Mean Square F Value Pr > F

*VRT-A2* 1 21.09 21.09 19.14 0.0002

*VRT-B2* 1 13.50 13.50 12.25 0.0020

Int. *VRT-A2* x *VRT-B2*  1 6.00 6.00 5.44 0.0292

Dunnett’s Test for days to heading **(growth chamber)**.

Genotype Difference

Comparison Between Means *P* value

vrt-A2 - WT 0.875 ns

vrt-B2 - WT 0.500 ns

vrt2-null - WT 3.375 <0.001

**Supplemental Figure 3B.** *SVP-A1* x *SVP-B1.* **Days to heading (growth chamber).**

Sum of

Source DF Squares Mean Square F Value Pr > F

Model 3 64.75 21.58 3.54 0.0285

Error 26 158.72 6.10

Corrected Total 29 223.47

R-Square: 0.289734

DF SS Mean Square F Value Pr > F

*SVP-A1* 1 14.91 14.91 2.44 0.1302

*SVP-B1* 1 9.96 9.96 1.63 0.2129

*SVP-A1* x *SVP-B1* 1 13.69 13.69 2.24 0.1463

Dunnett’s Test for days to heading **(growth chamber)**.

Genotype Difference

Comparison Between Means *P* value

svp-A1 - WT 0.067 ns

svp-B1 - WT -0.225 ns

svp1-null - WT 2.900 ns

**Supplemental Figure 3C.** *VRT-A2* x *VRT-B2.* **Spikelet number per spike (growth chamber)**.

Sum of

Source DF Squares Mean Square F Value Pr > F

Genotype 3 90.38 30.13 41.21 <.0001

Error 22 16.08 0.73

Corrected Total 25 106.46

R-Square: 0.848928

DF SS Mean Square F Value Pr > F

*VRT-A2* 1 70.04 70.04 95.81 <0.0001

*VRT-B2* 1 12.76 12.76 17.45 0.0004

Int. *VRT-A2* x *VRT-B2* 1 3.76 3.76 5.14 0.0335

Dunnett’s Test for spikelet number per spike **(growth chamber)**.

Genotype Difference

Comparison Between Means *P* value

vrt-A2 - WT 2.625 <0.001

vrt-B2 - WT 0.667 ns

vrt2-null - WT 4.875 <0.001

**Supplemental Figure 3D.** ***SVP-A1* x *SVP-B1.* Spikelet number per spike (growth chamber).**

Sum of

Source DF Squares Mean Square F Value Pr > F

Model 3 80.73 26.91 17.82 <0.0001

Error 26 39.27 1.51

Corrected Total 29 120.00

R-Square: 0.672778

DF SS Mean Square F Value Pr > F

*SVP-A1* 1 15.94 15.94 10.55 0.0032

*SVP-B1* 1 36.10 36.10 23.90 <0.0001

*SVP-A1* x *SVP-B1* 1 0.86 0.86 0.57 0.4572

Dunnett’s Test for spikelet number per spike **(growth chamber)**.

Genotype Difference

Comparison Between Means *P* value

svp-A1 - WT 1.267 ns

svp-B1 - WT 2.100 <0.05

svp1-null - WT 4.133 <0.001

**Supplemental Figure 3E** ***VRT-A2* x *VRT-B2.* Peduncle length (growth chamber).**

Sum of

Source DF Squares Mean Square F Value Pr > F

Model 3 2541.34 847.11 158.16 <0.0001

Error 28 149.97 5.36

Corrected Total 31 2691.31

R-Square: 0.944277

DF SS Mean Square F Value Pr > F

*VRT-A2* 1 980.14 980.14 183.00 <0.0001

*VRT-B2* 1 982.35 982.35 183.41 <0.0001

Int. *VRT-A2* x *VRT-B2* 1 578.85 578.85 108.07 <0.0001

Dunnett’s Test for peduncle length **(growth chamber)**.

Genotype Difference

Comparison Between Means *P* value

vrt-A2 - WT -2.563 ns

vrt-B2 - WT -2.575 ns

vrt2-null - WT -22.150 <0.001

**Supplemental Figure 3F** ***SVP-A1* x *SVP-B1.* Peduncle length (growth chamber).**

Sum of

Source DF Squares Mean Square F Value Pr > F

Model 3 615.63 205.21 33.84 <0.0001

Error 26 157.66 6.06

Corrected Total 29 773.29

R-Square: 0.796117

DF SS Mean Square F Value Pr > F

*SVP-A1* 1 19.48 19.48 3.21 0.0847

*SVP-B1* 1 342.50 342.50 56.48 <0.0001

Int. *SVP-A1* x *SVP-B1* 1 36.41 36.41 6.01 0.0213

Dunnett’s Test for peduncle length **(growth chamber)**.

Genotype Difference

Comparison Between Means *P* value

svp-A1 - WT 0.670 ns

svp-B1 - WT -5.155 <0.05

svp1-null - WT -9.473 <0.001

**Supplemental Figure 3E** ***VRT-A2* x *VRT-B2.* Internode 1 (closest to peduncle).**

Sum of

Source DF Squares Mean Square F Value Pr > F

Model 3 51.001 17.000 23.34 <0.0001

Error 28 20.397 0.728

Corrected Total 31 71.399

R-Square: 0.7143

DF SS Mean Square F Value Pr > F

*VRT-A2* 1 35.701 35.701 49.01 <0.0001

*VRT-B2* 1 0.720 0.720 0.99 0.3287

Int. *VRT-A2* x *VRT-B2* 1 14.580 14.580 20.01 <0.0001

Dunnett’s Test for internode 1 (closest to peduncle).

Genotype Difference

Comparison Between Means *P* value

vrt-A2 - WT 0.762 ns

vrt-B2 - WT -1.050 ns

vrt2-null - WT 2.412 <0.001

**Supplemental Figure 3F** ***SVP-A1* x *SVP-B1.* Internode 1 (closest to peduncle).**

Sum of

Source DF Squares Mean Square F Value Pr > F

Model 3 21.972 7.324 4.95 0.0075

Error 26 38.483 1.480

Corrected Total 29 60.455

R-Square: 0.3634

DF SS Mean Square F Value Pr > F

*SVP-A1* 1 6.639 6.639 4.49 0.0439

*SVP-B1* 1 10.122 10.122 6.84 0.0147

Int. *SVP-A1* x *SVP-B1* 1 0.618 0.618 0.42 0.5237

Dunnett’s Test for internode 1 (closest to peduncle).

Genotype Difference

Comparison Between Means *P* value

svp-A1 - WT 1.390 ns

svp-B1 - WT 1.640 ns

svp1-null - WT 2.380 <0.01

**Supplemental Figure 3E** ***VRT-A2* x *VRT-B2.* Internode 2 (from top).**

Sum of

Source DF Squares Mean Square F Value Pr > F

Model 3 5.953 1.984 7.71 0.0007

Error 28 7.208 0.257

Corrected Total 31 13.160

R-Square: 0.4523

DF SS Mean Square F Value Pr > F

*VRT-A2* 1 0.180 0.180 0.70 0.4101

*VRT-B2* 1 1.711 1.711 6.65 0.0155

Int. *VRT-A2* x *VRT-B2* 1 4.061 4.061 15.78 0.0005

Dunnett’s Test for internode 2 (from top).

Genotype Difference

Comparison Between Means *P* value

vrt-A2 - WT -0.562 ns

vrt-B2 - WT -1.175 <0.001

vrt2-null - WT -0.312 ns

**Supplemental Figure 3F** ***SVP-A1* x *SVP-B1.* Internode 2 (from top).**

Sum of

Source DF Squares Mean Square F Value Pr > F

Model 3 5.112 1.704 2.93 0.0522

Error 26 15.109 0.581

Corrected Total 29 20.222

R-Square: 0.2528

DF SS Mean Square F Value Pr > F

*SVP-A1* 1 0.074 0.074 0.13 0.7239

*SVP-B1* 1 2.505 2.505 4.31 0.0479

Int. *SVP-A1* x *SVP-B1* 1 0.634 0.634 1.09 0.3058

Dunnett’s Test for internode 2 (from top).

Genotype Difference

Comparison Between Means *P* value

svp-A1 - WT -0.217 ns

svp-B1 - WT 0.325 ns

svp1-null - WT 0.767 ns

**Supplemental Figure 3E** ***VRT-A2* x *VRT-B2.* Internode 3 (from top).**

Sum of

Source DF Squares Mean Square F Value Pr > F

Model 3 1.754 0.585 1.86 0.1606

Error 28 8.835 0.316

Corrected Total 31 10.588

R-Square: 0.1656

DF SS Mean Square F Value Pr > F

*VRT-A2* 1 0.151 0.151 0.48 0.4944

*VRT-B2* 1 0.551 0.551 1.75 0.1969

Int. *VRT-A2* x *VRT-B2* 1 1.051 1.051 3.33 0.0786

Dunnett’s Test for internode 3 (from top).

Genotype Difference

Comparison Between Means *P* value

vrt-A2 - WT -0.500 ns

vrt-B2 - WT -0.625 ns

vrt2-null - WT -0.400 ns

**Supplemental Figure 3F** ***SVP-A1* x *SVP-B1.* Internode 3 (from top).**

Sum of

Source DF Squares Mean Square F Value Pr > F

Model 3 2.979 0.993 1.01 0.4028

Error 26 25.487 0.980

Corrected Total 29 28.467

R-Square: 0.1046

DF SS Mean Square F Value Pr > F

*SVP-A1* 1 0.264 0.264 0.27 0.6080

*SVP-B1* 1 0.045 0.045 0.05 0.8324

Int. *SVP-A1* x *SVP-B1* 1 2.767 2.767 2.82 0.1049

Dunnett’s Test for internode 3 (from top).

Genotype Difference

Comparison Between Means *P* value

svp-A1 - WT -0.900 ns

svp-B1 - WT -0.775 ns

svp1-null - WT -0.300 ns

**Supplemental Figure 3E** ***VRT-A2* x *VRT-B2.* Internode 4 (from top).**

Sum of

Source DF Squares Mean Square F Value Pr > F

Model 3 3.062 1.021 0.88 0.4627

Error 28 32.437 1.158

Corrected Total 31 35.500

R-Square: 0.0863

DF SS Mean Square F Value Pr > F

*VRT-A2* 1 0.281 0.281 0.24 0.6261

*VRT-B2* 1 0.781 0.781 0.67 0.4185

Int. *VRT-A2* x *VRT-B2* 1 2.000 2.000 1.73 0.1995

Dunnett’s Test for internode 4 (from top).

Genotype Difference

Comparison Between Means *P* value

vrt-A2 - WT -0.687 ns

vrt-B2 - WT -0.812 ns

vrt2-null - WT -0.500 ns

**Supplemental Figure 3F** ***SVP-A1* x *SVP-B1.* Internode 4 (from top).**

Sum of

Source DF Squares Mean Square F Value Pr > F

Model 3 18.808 6.269 4.81 0.0085

Error 26 33.867 1.303

Corrected Total 29 52.675

R-Square: 0.3571

DF SS Mean Square F Value Pr > F

*SVP-A1* 1 4.919 4.919 3.78 0.0629

*SVP-B1* 1 9.146 9.146 7.02 0.0135

Int. *SVP-A1* x *SVP-B1* 1 5.283 5.283 4.06 0.0545

Dunnett’s Test for internode 4 (from top).

Genotype Difference

Comparison Between Means *P* value

svp-A1 - WT -1.867 <0.05

svp-B1 - WT -2.200 <0.01

svp1-null - WT -2.167 <0.05

**Supplemental Figure 3G** ***VRT-A2* x *VRT-B2.* Spikelet number per spike (field).**

Sum of

Source DF Squares Mean Square F Value Pr > F

Genotype 3 11.63 3.88 8.97 <.0001

Error 56 24.20 0.43

Corrected Total 59 35.82

R-Square: 0.324546

DF SS Mean Square F Value Pr > F

*VRT-A2* 1 3.96 3.96 9.17 0.0037

*VRT-B2* 1 3.54 3.54 8.18 0.0059

Int. *VRT-A2* x *VRT-B2* 1 0.39 0.39 0.89 0.3490

Dunnett’s Test for spikelet number per spike (field).

Genotype Difference

Comparison Between Means *P* value

vrt-A2 - WT 0.375 ns

vrt-B2 - WT 0.345 ns

vrt2-null - WT 1.060 <0.001

**Supplemental Figure 3H** ***VRT-A2* x *VRT-B2*. Plant height. Field.**

Sum of

Source DF Squares Mean Square F Value Pr > F

Genotype 3 10065.21 3355.07 698.44 <.0001

Error 56 269.01 4.80

Corrected Total 59 10334.22

R-Square: 0.973969

DF SS Mean Square F Value Pr > F

*VRT-A2* 1 2672.46 2672.46 556.34 <.0001

*VRT-B2* 1 2714.15 2714.15 565.01 <.0001

Int. *VRT-A2* x *VRT-B2* 1 1985.35 1985.35 413.30 <.0001

Dunnett’s Test for plant height (field).

Genotype Difference

Comparison Between Means *P* value

vrt-A2 - WT -1.955 ns

vrt-B2 - WT -2.065 ns

vrt2-null - WT -28.425 <0.001

**Supplemental Statistical Analyses for Supplemental Figure 4**

One-way ANOVA for transcript levels of flowering genes *FT1*, *VRN1*, and *VRN2* in the fifth leaf of single mutants *vrt2* and *svp1*, combined mutant *vrt2 svp1* and WT control, relative to *ACTIN*.

**Supplemental Figure 4A.** ***FT1*** relative transcript levels.

Sum of

Source DF Squares Mean Square F Value Pr > F

Genotype 3 7.981 2.660 3.70 1 0.0461

Error 11 7.904 0.719

Corrected Total 14 15.886

R-Square: 0.5024

Dunnett’s Test for ***FT1*** relative transcript levels.

Genotype Difference

Comparison Between Means *P* value

vrt2svp1 - WT -1.962 <0.05

vrt2 - WT -0.653 ns

svp1 - WT -0.876 ns

**Supplemental Figure 4B.** ***VRN1*** relative transcript levels.

Sum of

Source DF Squares Mean Square F Value Pr > F

Genotype 3 1.886 0.629 4.1 0.0351

Error 11 1.686 0.153

Corrected Total 14 3.572

R-Square: 0.5280

Dunnett’s Test for ***VRN1*** relative transcript levels.

Genotype Difference

Comparison Between Means *P* value

vrt2svp1 - WT -0.807 <0.05

vrt2 - WT 0.009 ns

svp1 - WT -0.034 ns

**Supplemental Figure 4C.** ***VRN2*** relative transcript levels Power transformation (^0.5) to restore normality of residuals and homogeneity of variances. ANOVA *P* values are the same in the transformed and untransformed data.

Sum of

Source DF Squares Mean Square F Value Pr > F

Genotype 3 1.339 0.446 69.66 <0.0001

Error 11 0.070 0.006

Corrected Total 14 1.410

R-Square: 0.9500

Dunnett’s Test for Days to heading. *P* values are from the transformed data but differences between means are untransformed to facilitate visualization.

Genotype Difference

Comparison Between Means *P* value

vrt2svp1 - WT 0.870 <0.001

vrt2 - WT 0.139 <0.01

svp1 - WT 0.230 <0.001

**Supplemental Statistical Analyses for Supplemental Figure 8**

One-way ANOVA for complementation of the *vrt2* mutation by the weak *UBI_pro_:VRT2* transgenic line T#8. Means are compared by Tukey tests (*P* < 0.05).

**Supplemental Figure 8A.** Days to heading.

Sum of

Source DF Squares Mean Square F Value Pr > F

Genotype 3 168.469 56.157 62.23 <0.0001

Error 42 37.900 0.902

Corrected Total 45 206.370

R-Square: 0.8163

DF SS Mean Square F Value Pr > F

*vrt2-null* 1 26.870 26.870 29.78 <0.0001

*UBI_pro_:VRT2-T#8* 1 100.584 100.584 111.47 <0.0001

Interaction 1 33.029 33.029 36.60 <0.0001

**Tukey Test** for days to heading.

Genotype Difference

Comparison Between Means *P <* 0.05

mutNT - WTNT 3.2333 *

mutNT - WTT 4.5000 *

mutNT - mutT 4.6667 *

WTNT - mutNT -3.2333 *

WTNT - WTT 1.2667 *

WTNT - mutT 1.4333 *

WTT - mutNT -4.5000 *

WTT - WTNT -1.2667 *

WTT - mutT 0.1667 ns

mutT - mutNT -4.6667 *

mutT - WTNT -1.4333 *

mutT - WTT -0.1667 ns

Mut= *vrt2-null*, WT= functional *VRT2*, T= transgenic, NT= not transgenic

**Supplemental Figure 8B.** Peduncle length.

Sum of

Source DF Squares Mean Square F Value Pr > F

Genotype 3 3031.48 1010.49 149.77 <0.0001

Error 42 283.38 6.75

Corrected Total 45 3314.86

R-Square: 0.9145

DF SS Mean Square F Value Pr > F

*vrt2-null* 1 2807.77 2807.77 416.14 <0.0001

*UBI_pro_:VRT2-T#8* 1 53.28 53.28 7.90 0.0075

Interaction 1 177.49 177.49 26.31 <0.0001

**Tukey Test** for peduncle length.

Genotype Difference

Comparison Between Means *P <* 0.05

WTNT - WTT 1.782 ns

WTNT - mutT 13.515 *

WTNT - mutNT 19.615 *

WTT - WTNT -1.782 ns

WTT - mutT 11.733 *

WTT - mutNT 17.833 *

mutT - WTNT -13.515 *

mutT - WTT -11.733 *

mutT - mutNT 6.100 *

mutNT - WTNT -19.615 *

mutNT - WTT -17.833 *

mutNT - mutT -6.100 *

Mut= *vrt2-null*, WT= functional *VRT2*, T= transgenic, NT= not transgenic

**Supplemental Figure 8C.** Spikelet number per spike.

Sum of

Source DF Squares Mean Square F Value Pr > F

Genotype 3 142.233 47.411 47.68 <0.0001

Error 42 41.767 0.994

Corrected Total 45 184.000

R-Square: 0.7730

DF SS Mean Square F Value Pr > F

*vrt2-null* 1 141.337 141.337 142.13 <0.0001

*UBI_pro_:VRT2-T#8* 1 0.813 0.813 0.82 0.3711

Interaction 1 2.146 2.146 2.16 0.1493

Tukey Test for spikelet number per spike.

Genotype Difference

Comparison Between Means *P <* 0.05

mutNT - mutT 0.1667 ns

mutNT - WTT 3.2500 *

mutNT - WTNT 3.9500 *

mutT - mutNT -0.1667 ns

mutT - WTT 3.0833 *

mutT - WTNT 3.7833 *

WTT - mutNT -3.2500 *

WTT - mutT -3.0833 *

WTT - WTNT 0.7000 ns

WTNT - mutNT -3.9500 *

WTNT - mutT -3.7833 *

WTNT - WTT -0.7000 ns

Mut= *vrt2-null*, WT= functional *VRT2*, T= transgenic, NT= not transgenic

**Supplemental Statistical Analyses for Supplemental Figure 11.**

*t-*tests for the effect of *vrt2* mutation in partial mutant *Vrn1 ful2* (underline indicates heterozygous *Vrn-A1 vrn-A1* and homozygous *vrn-B1 vrn-B1*). All lines in this table are in the *Vrn1 ful2* genetic background.

**11.A**: days to heading (DTH), **11.B**: stem length, **11.C**: spikelet number per pike (SNS)

|  | 11.A: DTH | | |  | | 11.B: Stem length | | |  | | 11.C: SNS | | |
| --- | --- | --- | --- | --- | --- | --- | --- | --- | --- | --- | --- | --- | --- |
| Rep. | *Vrt2* | *vrt2* | *Vrt2* | | | | | *vrt2* | | *Vrt2* | | | *vrt2* |
| 1 | 50 | 55 |  | | 37.0 | | 34.0 | |  | | 30 | 33 | |
| 2 | 52 | 56 |  | | 33.5 | | 36.0 | |  | | 29 | 30 | |
| 3 | 49 | 56 |  | | 35.5 | | 38.0 | |  | | 28 | 31 | |
| 4 | 51 | 55 |  | | 39.2 | | 36.5 | |  | | 24 | 30 | |
| 5 | 48 | 52 |  | | 32.2 | | 29.0 | |  | | 24 | 28 | |
| 6 | 47 | 55 |  | | 36.0 | | 35.0 | |  | | 22 | 30 | |
| 7 | 50 | 49 |  | | 39.5 | | 33.5 | |  | | 24 | 30 | |
| 8 | 54 | 50 |  | | 38.0 | | 31.2 | |  | | 24 | 30 | |
| 9 | 49 |  |  | | 36.6 | |  | |  | | 25 |  | |
| Mean | 50.0 | 53.5 |  | | 36.4 | | 34.2 | |  | | 25.6 | 30.3 | |
| SE | 0.71 | 0.98 |  | | 0.81 | | 1.04 | |  | | 0.91 | 0.49 | |
| ***t*-Test** | **0.010** | | |  | | **0.106** | | |  | | **0.0006** | | |

**11.D**: glume one length, **11.E**: lemma one length, **11.F**: % spikelets transformed into branches

|  | 11.D: Glume one length (cm) | |  | 11.E: Lemma one length (cm) | |  | 11.F: % branch | |
| --- | --- | --- | --- | --- | --- | --- | --- | --- |
| Rep. | *Vrt2* | *vrt2* |  | *Vrt2* | *vrt2* |  | *Vrt2* | *vrt2* |
| 1 | 2.41 | 1.51 |  | 5.06 | 3.59 |  | 85.7 | 28.6 |
| 2 | 2.42 | 1.61 |  | 3.75 | 2.30 |  | 85.7 | 57.1 |
| 3 | 2.01 | 1.46 |  | 3.93 | 2.82 |  | 100 | 25.0 |
| 4 | 2.05 | 1.52 |  | 3.02 | 3.15 |  | 71.4 | 42.9 |
| 5 | 2.33 | 1.63 |  | 5.41 | 2.61 |  | 71.4 | 28.6 |
| 6 | 2.30 | 1.57 |  | 3.66 | 5.24 |  | 71.4 | 25.0 |
| 7 | 2.01 | 1.50 |  | 2.81 | 3.40 |  | 100 | 14.3 |
| 8 | 1.94 | 1.66 |  | 3.58 | 6.34 |  | 57.1 |  |
| 9 | 1.94 | 1.80 |  | 3.25 | 2.74 |  |  |  |
| 10 | 2.56 | 1.78 |  | 4.32 | 3.86 |  |  |  |
| 11 | 1.86 | 1.73 |  | 2.84 | 2.22 |  |  |  |
| 12 | 2.19 | 1.58 |  | 3.56 | 2.78 |  |  |  |
| 13 | 2.09 | 1.69 |  | 3.52 | 2.46 |  |  |  |
| 14 | 1.76 | 1.64 |  | 4.23 | 4.57 |  |  |  |
| 15 | 2.35 | 1.51 |  | 5.08 | 2.16 |  |  |  |
| 16 | 1.99 | 1.61 |  | 5.82 | 4.24 |  |  |  |
| 17 | 1.86 | 1.63 |  | 5.83 | 3.02 |  |  |  |
| 18 | 2.38 | 1.43 |  | 4.24 | 3.36 |  |  |  |
| 19 | 3.04 | 1.82 |  | 4.45 | 1.94 |  |  |  |
| 20 | 2.44 | 1.89 |  | 4.43 | 1.77 |  |  |  |
| 21 | 1.90 | 1.66 |  | 4.30 | 2.31 |  |  |  |
| 22 | 2.74 | 1.46 |  | 4.58 | 4.32 |  |  |  |
| 23 | 2.48 | 1.65 |  | 2.80 | 1.92 |  |  |  |
| 24 | 2.77 | 1.51 |  | 7.03 | 1.90 |  |  |  |
| 25 | 2.46 | 1.75 |  | 6.37 | 4.40 |  |  |  |
| 26 | 2.10 | 1.78 |  | 3.40 | 3.66 |  |  |  |
| 27 | 2.37 |  |  | 5.21 | 2.65 |  |  |  |
| 28 | 1.67 |  |  | 4.47 | 1.63 |  |  |  |
| 29 | 2.03 |  |  | 4.30 |  |  |  |  |
| 30 | 1.69 |  |  | 4.97 |  |  |  |  |
| 31 | 2.06 |  |  |  |  |  |  |  |
| 32 | 2.55 |  |  |  |  |  |  |  |
| 33 | 3.03 |  |  |  |  |  |  |  |
| Mean | 2.2 | 1.6 |  | 4.3 | 3.1 |  | 80.3 | 31.6 |
| SE | 0.06 | 0.02 |  | 0.19 | 0.22 |  | 5.06 | 4.96 |
| *t*-Test | **2.2E-11** | |  | **9.2E-05** | |  | **2E-05** | |

**Supplemental Statistical Analyses for Supplemental Figure 12.**

Effect of *VRT2* and *SVP1* on the transcript levels of *SEPALLATA*, *CEN* and *TB1-2* genes (relative to *ACTIN*) in developing spikes.

**Supplemental Figure 12.A**. *t-tests* for transcript levels between *Vrn1 ful2* (*Vrt2*) and *vrt2 Vrn1 ful2* (*vrt2*) at the post double ridge (PDR) and terminal spikelet (TS) stages. All lines in this table are in the *Vrn1 ful2* genetic background.

|  |  |  |  |  |  |  |  |  |  |  |  |
| --- | --- | --- | --- | --- | --- | --- | --- | --- | --- | --- | --- |
|  | *OsMADS34 = SEP1-6* | | | | |  | *OsMADS5 = SEP1-4* | | | | |
|  | PDR | |  | TS | |  | PDR | |  | TS | |
| Rep | *VRT2* | *vrt2* |  | *VRT2* | *vrt2* |  | *VRT2* | *vrt2* |  | *VRT2* | *vrt2* |
| 1 | 31.220 | 15.559 |  | 26.630 | 20.240 |  | 0.523 | 4.183 |  | 1.004 | 0.804 |
| 2 | 19.939 | 18.059 |  | 9.617 | 22.562 |  | 0.354 | 1.805 |  | 4.056 | 1.103 |
| 3 | 14.002 | 17.015 |  | 13.247 | 12.486 |  | 1.232 | 0.213 |  | 8.080 | 7.322 |
| 4 | 15.317 | 14.498 |  | 28.449 | 8.162 |  | 0.718 | 0.646 |  | 0.742 | 3.707 |
| Mean | 20.12 | 16.28 |  | 19.49 | 15.86 |  | 0.71 | 1.71 |  | 3.47 | 3.23 |
| SE | 1.56 | 0.92 |  | 5.00 | 3.69 |  | 0.22 | 0.41 |  | 1.84 | 1.56 |
| *t-*test | 0.3735 | |  | 0.5546 | |  | 0.3117 | |  | 0.9208 | |

|  | *OsMADS1 = SEP1-2* | | | | |  | *OsMADS7 = SEP3-1* | | | | | | |
| --- | --- | --- | --- | --- | --- | --- | --- | --- | --- | --- | --- | --- | --- |
|  | PDR | |  | TS | |  | PDR | | |  | | TS | |
| Rep | *VRT2* | *vrt2* |  | *VRT2* | *vrt2* |  | *VRT2* | *vrt2* | |  | | *VRT2* | *vrt2* |
| 1 | 0.154 | 0.634 |  | 1.408 | 4.439 |  | 0.001 | 0.025 | |  | | 1.870 | 1.937 |
| 2 | 0.035 | 0.223 |  | 0.979 | 2.560 |  | 0.000 | 0.032 | |  | | 1.383 | 1.490 |
| 3 | 0.070 | 0.030 |  | 1.932 | 3.952 |  | 0.002 | 0.020 | |  | | 2.924 | 3.102 |
| 4 | 0.032 | 0.118 |  | 1.532 | 2.446 |  | 0.003 | 0.008 | |  | | 1.762 | 1.364 |
| Mean | 0.07 | 0.25 |  | 1.46 | 3.35 |  | 0.001 | 0.021 | |  | | 1.98 | 1.97 |
| SE | 0.03 | 0.13 |  | 0.20 | 0.50 |  | 0.0001 | 0.01 | |  | | 0.33 | 0.40 |
| *t-*test | 0.240 | |  | 0.0126 | |  | 0.0087 | |  | | 0.9831 | | |

|  | *CEN2* | | | | |  | *CEN4* | | | | |
| --- | --- | --- | --- | --- | --- | --- | --- | --- | --- | --- | --- |
|  | PDR | |  | TS | |  | PDR | |  | TS | |
| Rep | *VRT2* | *vrt2* |  | *VRT2* | *vrt2* |  | *VRT2* | *vrt2* |  | *VRT2* | *vrt2* |
| 1 | 1.312 | 0.418 |  | 1.113 | 0.828 |  | 0.870 | 0.812 |  | 1.038 | 1.090 |
| 2 | 2.291 | 0.439 |  | 1.546 | 0.576 |  | 0.657 | 0.576 |  | 1.607 | 1.182 |
| 3 | 1.361 | 0.775 |  | 1.631 | 1.226 |  | 0.749 | 0.399 |  | 1.965 | 2.138 |
| 4 | 2.624 | 0.959 |  | 1.712 | 0.733 |  | 0.800 | 0.646 |  | 1.501 | 1.341 |
| Mean | 1.90 | 0.65 |  | 1.50 | 0.84 |  | 0.77 | 0.61 |  | 1.53 | 1.44 |
| SE | 0.33 | 0.13 |  | 0.13 | 0.14 |  | 0.04 | 0.09 |  | 0.19 | 0.24 |
| *t-*test | 0.0127 | |  | 0.014 | |  | 0.148 | |  | 0.779 | |

|  | *CEN5* | | | | |  | *TB1-2* | | | | |
| --- | --- | --- | --- | --- | --- | --- | --- | --- | --- | --- | --- |
|  | PDR | |  | TS | |  | PDR | |  | TS | |
| Rep | *VRT2* | *vrt2* |  | *VRT2* | *vrt2* |  | *VRT2* | *vrt2* |  | *VRT2* | *vrt2* |
| 1 | 1.275 | 0.232 |  | 5.612 | 1.761 |  | 0.296 | 0.112 |  | 0.703 | 0.538 |
| 2 | 1.289 | 0.206 |  | 1.299 | 3.621 |  | 0.199 | 0.089 |  | 0.372 | 0.375 |
| 3 | 0.509 | 0.231 |  | 1.561 | 0.997 |  | 0.164 | 0.103 |  | 0.514 | 0.651 |
| 4 | 0.901 | 0.299 |  | 5.221 | 0.500 |  | 0.185 | 0.151 |  | 0.656 | 0.191 |
| Mean | 0.99 | 0.24 |  | 3.42 | 1.72 |  | 0.21 | 0.11 |  | 0.56 | 0.44 |
| SE | 0.18 | 0.02 |  | 1.15 | 0.68 |  | 0.03 | 0.01 |  | 0.07 | 0.10 |
| *t-*test | 0.0068 | |  | 0.2515 | |  | 0.0231 | |  | 0.3654 | |

**Supplemental Figure 12B.** 2 x 2 factorial ANOVA *VRT2* x *SVP1*. Comparison of *CEN2*, *CEN4*, *CEN5*, *TB1-2* transcript levels among WT, *vrt2*, *svp1* and *vrt2 svp1* lines at developing spikes (PDR). Transcript levels were determined by qRT-PCR using *ACTIN* as endogenous control.

**Figure 12B (1^st^ left). *CEN2***. Power transformation (^0.01) to restore normality of residuals and homogeneity of variances. ANOVA *P* values are the same in the transformed and untransformed data.

Sum of

Source DF Squares Mean Square F Value Pr > F

Genotype 3 0.00353 0.00118 24.76 <0.0001

Error 12 0.00057 0.00004

Corrected Total 15 0.00410

R-Square: 0.8609

DF SS Mean Square F Value Pr > F

vrt2 1 0.00174 0.00174 36.53 <0.0001

svp1 1 0.00112 0.00112 23.61 0.0004

Interaction vrt2 x svp1 1 0.00067 0.00067 14.13 0.0027

Dunnett’s Test for *CEN2*. *P* values are from the transformed data but differences between means are untransformed to facilitate visualization.

Genotype Difference

Comparison Between Means *P* value

vrt2svp1 - WT -1.2406 <0.001

vrt2 - WT -0.7966 ns

svp1 - WT -0.5452 ns

**Figure 12B (2^nd^ from left). *CEN4***. Power transformation (^0.001) to restore normality of residuals and homogeneity of variances. ANOVA *P* values are the same in the transformed and untransformed data.

Sum of

Source DF Squares Mean Square F Value Pr > F

Genotype 3 0.000058 0.000019 16.51 <0.0001

Error 12 0.000014 0.000001

Corrected Total 15 0.000073

R-Square: 0.8049

DF SS Mean Square F Value Pr > F

vrt2 1 0.000031 0.000031 25.93 0.0003

svp1 1 0.000018 0.000018 15.00 0.0022

Interaction vrt2 x svp1 1 0.000010 0.000010 8.59 0.0126

Dunnett’s Test for *CEN4*. *P* values are from the transformed data but differences between means are untransformed to facilitate visualization.

Genotype Difference

Comparison Between Means *P* value

vrt2svp1 - WT -1.5120 <0.001

vrt2 - WT -1.1650 ns

svp1 - WT -0.8814 ns

**Figure 12B (3^rd^ from left). *CEN5***. Power transformation (^-0.5) to restore normality of residuals and homogeneity of variances. ANOVA *P* values are the same in the transformed and untransformed data.

Sum of

Source DF Squares Mean Square F Value Pr > F

Genotype 3 2.201 0.734 20.60 <0.0001

Error 12 0.427 0.036

Corrected Total 15 2.628

R-Square: 0.8374

DF SS Mean Square F Value Pr > F

vrt2 1 1.259 1.259 35.35 >0.0001

svp1 1 0.480 0.480 13.48 0.0032

Interaction vrt2 x svp1 1 0.462 0.462 12.96 0.0036

Dunnett’s Test *CEN5*. *P* values are from the transformed data but differences between means are untransformed to facilitate visualization.

Genotype Difference

Comparison Between Means *P* value

vrt2svp1 - WT -0.7715 <0.001

vrt2 - WT -0.3847 ns

svp1 - WT 0.0026 ns

**Figure 12B (4^th^ from left). *TB1-2***. Power transformation (^0.05) to restore normality of residuals and homogeneity of variances. ANOVA *P* values are the same in the transformed and untransformed data.

Sum of

Source DF Squares Mean Square F Value Pr > F

Genotype 3 0.189 0.063 6.88 0.0060

Error 12 0.110 0.009

Corrected Total 15 0.298

R-Square: 0.6323

DF SS Mean Square F Value Pr > F

vrt2 1 0.073 0.073 7.94 0.0155

svp1 1 0.065 0.065 7.10 0.0206

Interaction vrt2 x svp1 1 0.051 0.051 5.59 0.0357

Dunnett’s Test for *TB1-2*. *P* values are from the transformed data but differences between means are untransformed to facilitate visualization.

Genotype Difference

Comparison Between Means *P* value

vrt2svp1 - WT -0.1704 <0.01

vrt2 - WT -0.0299 ns

svp1 - WT -0.0291 ns
