# Supplementary material for: Interactions between SQUAMOSA and SHORT VEGETATIVE PHASE MADS-box proteins regulate meristem transitions during wheat spike development
Source: Plant Cell. 2021 Nov 2;33(12):3621–44. doi: 10.1093/plcell/koab243 (PMC8643710; doi:10.1093/plcell/koab243)
Supplement: koab243_Supplementary_Data [file koab243_supplementary_data.zip › tpc.21.00586_SupplementalFile1.docx]

**Supplemental File 1.** Sequence alignment used to produce the phylogenetic tree in Supplemental Figure 1.

Alignment of *Triticum aestivum* (Ta), *Hordeum vulgare* (Hv), *Oryza sativa* (Os) and *Arabidopsis thaliana* (At) MADS-box proteins from the SVP-clade using MUSCLE in MEGA X. Poorly aligned regions at the beginning and end of the proteins were truncated and were not used in the calculation of the phylogenetic tree. The same alignment in NEXUS format is presented in the second page.

TaVRT-A2 MARERRAIRRIESAAARQVTFSKRRRGLFKKAEELAVLCDADVALVVFSSTGKLSQFASSSMNEIIDKYSTHSKNLGKSD
TaVRT-B2 MARERRAIRRIESAAARQVTFSKRRRGLFKKAEELAVLCDADVALVVFSSTGKLSQFASSSMNEIIDKYSTHSKNLGKSD
HvVRT2 MARERRAIRRIESAAARQVTFSKRRRGLFKKAEELAVLCDADVALVVFSSTGKLSQFASSSMNEIIDKYSTHSKNLGKSD
OsMADS55 MARERREIRRIESAAARQVTFSKRRRGLFKKAEELAVLCDADVALVVFSSTGKLSQFASSNMNEIIDKYTTHSKNLGKTD
TaSVP-A1 MARERREIKRIESAAARQVTFSKRRRGLFKKAEELSVLCDADVALIVFSSTGKLSQFASSSMNEIIDKYSTHSKNLGKT-
TaSVP-B1 MARERREIKRIESAAARQVTFSKRRRGLFKKAEELSVLCDADVALIVFSSTGKLSQFASSSMNEIIDKYSTHSKNLGKT-
HvBM10 MARERREIKRIESAAARQVTFSKRRRGLFKKAEELSVLCDADVALIVFSSTGKLSQFASSSMNEIIDKYSTHSKNLGKT-
OsMADS22 MARERREIKRIESAAARQVTFSKRRRGLFKKAEELSVLCDADVALIVFSSTGKLSHFASSSMNEIIDKYNTHSNNLGKA-
HvBM1 GKRERIAIRRIENLAARQVTFSKRRRGLFKKAEELSILCDAEVGLAVFSATGKLFQFASSSMNQIIDRYNSHSKILKKVD
OsMADS47 GKRERIAIRRIDNLAARQVTFSKRRRGLFKKAEELSILCDAEVGLVVFSATGKLFQFASTSMEQIIDRYNSHSKTLQRA-
TaSVP-A3 GRRERIAIRRIENLAARQVTFSKRRRGLFKKAEELSILCDAEVGLAVFSATGKLFQFASSSMNQIIDRYNSHSKILKKAD
TaSVP-B3 GKRERIAIRRIENLAARQVTFSKRRRGLFKKAGELSILCDAEVGLAVFSATGKLFQFASSSMNQIIDRYNSHSKILKKAD
AtSVP MAREKIQIRKIDNATARQVTFSKRRRGLFKKAEELSVLCDADVALIIFSSTGKLFEFCSSSMKEVLERHNLQSKNLEKL-
AtAGL24 MAREKIRIKKIDNITARQVTFSKRRRGIFKKADELSVLCDADVALIIFSATGKLFEFSSSRMRDILGRYSLHASNINKLM
consensus ..**...*..*....************.****.**..****.*.*..**.****..*.*..*..................


TaVRT-A2 QQPAIDLN-LEHCKYDSLNEQLAEASLRLRHMRGEELDGLSVGELQQMEKNLETGLQRVLCTKDRQFMQQISDLQQKGTQ
TaVRT-B2 QQPAIDLN-LEHCKYDSLNEQLAEASLRLRRMRGEELDGLSVGELQQMEKNLETGLQRVLCTKDRQFMQQINDLQQKGTQ
HvVRT2 QQPAIDLN-LEHCKYDSLNEQLAEASLRLRHMRGEELDGLSVGELQQMEKNLETGLQRVLCTKDRQFMQQISDLQQKGTQ
OsMADS55 KQPSIDLN-LEHSKCSSLNEQLAEASLQLRQMRGEELEGLSVEELQQMEKNLEAGLQRVLCTKDQQFMQEISELQRKGIQ
TaSVP-A1 DQPALDLN-LEHSKYANLNDQLAEASLRLRQMRGEELEGLSVDELQQLEKNLETGLHRVLQTKDQQFLEQINELHRKSSQ
TaSVP-B1 DQPALDLN-LEHSKYANLNDQLAEASLRLRQMRGEELEGLSVDELQQLEKNLETGLHRVLQTKDQQFLEQINELHRKSSQ
HvBM10 DQPTLDLN-LEHSKYANLNDQLAEASLRLRQMRGEELEGLSVDELQQLEKNLETGLHKVLQTKDQQFLEQINELHRKSSQ
OsMADS22 EQPSLDLN-LEHSKYAHLNEQLAEASLRLRQMRGEELEGLSIDELQQLEKNLEAGLHRVMLTKDQQFMEQISELQRKSSQ
HvBM1 EPSQLDLH--EDSNCARLRDELAEASLWLQQMRGEELQSLNVQQLQALEKSLESGLSSVLKTKSQKIMDQISELEKKRVQ
OsMADS47 EPSQLDLQGEDSSTCARLKEELAETSLRLRQMRGEELHRLNVEQLQELEKSLESGLGSVLKTKSKKILDEIDGLERKRMQ
TaSVP-A3 EPSQLDLH--EDSNCARLRDELAEASLWLQQMRGEELQSLNVQQLQALEKSLESGLGSVLKTKSQKIMDQISELERKRVQ
TaSVP-B3 EPSQLDLH--EDSNCARLRDELAEASLWLQQMRGEELQSLNVQQLQALEKSLESGLGSVLKTKSQKIMDQISELERKRVQ
AtSVP DQPSLELQLVENSDHARMSKEIADKSHRLRQMRGEELQGLDIEELQQLEKALETGLTRVIETKSDKIMSEISELQKKGMQ
AtAGL24 DPPSTHLR-LENCNLSRLSKEVEDKTKQLRKLRGEDLDGLNLEELQRLEKLLESGLSRVSEKKGECVMSQIFSLEKRGSE
consensus ... ..*. ...................*...***.*..*....**..**.**.** .*. .*.......*..* .. ..


TaVRT-A2 LAEENMRLKNQMHEVPTA-STVAVAE--AENVVPEDAHSSDSVMTAVHSGS--SQDNDDGSDISLKLALP---WK
TaVRT-B2 LAEENMRLKNQMHEVPTA-SMVAVADADAENVVPDDVHSSDSVMTAVHSAS--SQDNDDGSDISLKLALP---WK
HvVRT2 LAEENMRLKNQMHEVPTA-SMVAVAD-----VVPEDVHSSDSVMTAVHSAS--SQDNDDGSDISLKLALP---WK
OsMADS55 LAEENMRLRDQMPQVPT--AGLAVPD--TENVLTEDGQSSESVMTALNSGS--SQDNDDGSDISLKLGLP-----
TaSVP-A1 LAEENMKLRNQVGQIPTA-GKLVVAD--TENVVAEDGQSSESVMTALHSGS--SQDNDDGSDVSLKLGLPCLPWK
TaSVP-B1 LAEENMKLRNQVGQIPTA-GKLVVAD--TENIVAEDGQSSESVMTALHSGS--SQDNDDGSDVSLKLGLPCLPWK
HvBM10 LAEENKKLRNQVAQVPTA-GKLVVVD--TENVIAEDGQSSESVMTALHSGS--SQDNDDGSDVSLKLALP---WK
OsMADS22 LAEENMQLRNQVSQISPA-EKQVV-D--TENFVTE-GQSSESVMTALHSGSSQSQDNDDGSDVSLKLGLPCGAWK
HvBM1 LIEENARLKEQASKM----EMQVAAD--PLVVVYDEGQSSESVTNTSYPRP--PLDTEDSSDTSLRLGLSLFNSK
OsMADS47 LIEENLRLKEQVSRMSRMEEMQPGPD--S-EIVYEEGQSSESVTNASYPRP--PPDNDYSSDTSLKLGLHS----
TaSVP-A3 LIEENARLKEQASKM----EMQVAAD--S-PVVYEEGQSSESVTNTSYPRP--PLDTEDSSDTSLRLGLPLYNS-
TaSVP-B3 LIEENARLKEQASKM----EMQVAAD--S-PAVYEEGQSSESVTNTSYPRP--PLDTEDSSDTSLRLGLPLYNSK
AtSVP LMDENKRLRQQGTQLTEENERLGMAES-ENAAVYEEGQSSESITNAGNSTG--APVDSESSDTSLRLGLPYGG--
AtAGL24 LVDENKRLRDKLETLER-----------AKLTTLKEALETESVTTNVSSYDS-GTPLEDDSDTSLKLGLPS--WE
consensus *..**..*.... .... ..... ..... .......*......... .......**.**.*.*. ..

NEXUS format

#NEXUS

[ TITLE ]

BEGIN TAXA;

DIMENSIONS NTAX=14;

TAXLABELS

TaVRT-A2

TaVRT-B2

HvVRT2

OsMADS55

TaSVP-A1

TaSVP-B1

HvBM10

OsMADS22

HvBM1

OsMADS47

TaSVP-A3

TaSVP-B3

AtSVP

AtAGL24

;

END;

BEGIN CHARACTERS;

DIMENSIONS NCHAR=236;

FORMAT MISSING=? GAP=- MATCHCHAR=. datatype=protein;

MATRIX

TaVRT-A2 MARERRAIRRIESAAARQVTFSKRRRGLFKKAEELAVLCDADVALVVFSSTGKLSQFASSSMNEIIDKYSTHSKNLGKSDQQPAIDLN-LEHCKYDSLNEQLAEASLRLRHMRGEELDGLSVGELQQMEKNLETGLQRVLCTKDRQFMQQISDLQQKGTQLAEENMRLKNQMHEVPTA-STVAVAE--AENVVPEDAHSSDSVMTAVHSGS--SQDNDDGSDISLKLALP---WK

TaVRT-B2 MARERRAIRRIESAAARQVTFSKRRRGLFKKAEELAVLCDADVALVVFSSTGKLSQFASSSMNEIIDKYSTHSKNLGKSDQQPAIDLN-LEHCKYDSLNEQLAEASLRLRRMRGEELDGLSVGELQQMEKNLETGLQRVLCTKDRQFMQQINDLQQKGTQLAEENMRLKNQMHEVPTA-SMVAVADADAENVVPDDVHSSDSVMTAVHSAS--SQDNDDGSDISLKLALP---WK

HvVRT2 MARERRAIRRIESAAARQVTFSKRRRGLFKKAEELAVLCDADVALVVFSSTGKLSQFASSSMNEIIDKYSTHSKNLGKSDQQPAIDLN-LEHCKYDSLNEQLAEASLRLRHMRGEELDGLSVGELQQMEKNLETGLQRVLCTKDRQFMQQISDLQQKGTQLAEENMRLKNQMHEVPTA-SMVAVAD-----VVPEDVHSSDSVMTAVHSAS--SQDNDDGSDISLKLALP---WK

OsMADS55 MARERREIRRIESAAARQVTFSKRRRGLFKKAEELAVLCDADVALVVFSSTGKLSQFASSNMNEIIDKYTTHSKNLGKTDKQPSIDLN-LEHSKCSSLNEQLAEASLQLRQMRGEELEGLSVEELQQMEKNLEAGLQRVLCTKDQQFMQEISELQRKGIQLAEENMRLRDQMPQVPT--AGLAVPD--TENVLTEDGQSSESVMTALNSGS--SQDNDDGSDISLKLGLP-----

TaSVP-A1 MARERREIKRIESAAARQVTFSKRRRGLFKKAEELSVLCDADVALIVFSSTGKLSQFASSSMNEIIDKYSTHSKNLGKT-DQPALDLN-LEHSKYANLNDQLAEASLRLRQMRGEELEGLSVDELQQLEKNLETGLHRVLQTKDQQFLEQINELHRKSSQLAEENMKLRNQVGQIPTA-GKLVVAD--TENVVAEDGQSSESVMTALHSGS--SQDNDDGSDVSLKLGLPCLPWK

TaSVP-B1 MARERREIKRIESAAARQVTFSKRRRGLFKKAEELSVLCDADVALIVFSSTGKLSQFASSSMNEIIDKYSTHSKNLGKT-DQPALDLN-LEHSKYANLNDQLAEASLRLRQMRGEELEGLSVDELQQLEKNLETGLHRVLQTKDQQFLEQINELHRKSSQLAEENMKLRNQVGQIPTA-GKLVVAD--TENIVAEDGQSSESVMTALHSGS--SQDNDDGSDVSLKLGLPCLPWK

HvBM10 MARERREIKRIESAAARQVTFSKRRRGLFKKAEELSVLCDADVALIVFSSTGKLSQFASSSMNEIIDKYSTHSKNLGKT-DQPTLDLN-LEHSKYANLNDQLAEASLRLRQMRGEELEGLSVDELQQLEKNLETGLHKVLQTKDQQFLEQINELHRKSSQLAEENKKLRNQVAQVPTA-GKLVVVD--TENVIAEDGQSSESVMTALHSGS--SQDNDDGSDVSLKLALP---WK

OsMADS22 MARERREIKRIESAAARQVTFSKRRRGLFKKAEELSVLCDADVALIVFSSTGKLSHFASSSMNEIIDKYNTHSNNLGKA-EQPSLDLN-LEHSKYAHLNEQLAEASLRLRQMRGEELEGLSIDELQQLEKNLEAGLHRVMLTKDQQFMEQISELQRKSSQLAEENMQLRNQVSQISPA-EKQVV-D--TENFVTE-GQSSESVMTALHSGSSQSQDNDDGSDVSLKLGLPCGAWK

HvBM1 GKRERIAIRRIENLAARQVTFSKRRRGLFKKAEELSILCDAEVGLAVFSATGKLFQFASSSMNQIIDRYNSHSKILKKVDEPSQLDLH--EDSNCARLRDELAEASLWLQQMRGEELQSLNVQQLQALEKSLESGLSSVLKTKSQKIMDQISELEKKRVQLIEENARLKEQASKM----EMQVAAD--PLVVVYDEGQSSESVTNTSYPRP--PLDTEDSSDTSLRLGLSLFNSK

OsMADS47 GKRERIAIRRIDNLAARQVTFSKRRRGLFKKAEELSILCDAEVGLVVFSATGKLFQFASTSMEQIIDRYNSHSKTLQRA-EPSQLDLQGEDSSTCARLKEELAETSLRLRQMRGEELHRLNVEQLQELEKSLESGLGSVLKTKSKKILDEIDGLERKRMQLIEENLRLKEQVSRMSRMEEMQPGPD--S-EIVYEEGQSSESVTNASYPRP--PPDNDYSSDTSLKLGLHS----

TaSVP-A3 GRRERIAIRRIENLAARQVTFSKRRRGLFKKAEELSILCDAEVGLAVFSATGKLFQFASSSMNQIIDRYNSHSKILKKADEPSQLDLH--EDSNCARLRDELAEASLWLQQMRGEELQSLNVQQLQALEKSLESGLGSVLKTKSQKIMDQISELERKRVQLIEENARLKEQASKM----EMQVAAD--S-PVVYEEGQSSESVTNTSYPRP--PLDTEDSSDTSLRLGLPLYNS-

TaSVP-B3 GKRERIAIRRIENLAARQVTFSKRRRGLFKKAGELSILCDAEVGLAVFSATGKLFQFASSSMNQIIDRYNSHSKILKKADEPSQLDLH--EDSNCARLRDELAEASLWLQQMRGEELQSLNVQQLQALEKSLESGLGSVLKTKSQKIMDQISELERKRVQLIEENARLKEQASKM----EMQVAAD--S-PAVYEEGQSSESVTNTSYPRP--PLDTEDSSDTSLRLGLPLYNSK

AtSVP MAREKIQIRKIDNATARQVTFSKRRRGLFKKAEELSVLCDADVALIIFSSTGKLFEFCSSSMKEVLERHNLQSKNLEKL-DQPSLELQLVENSDHARMSKEIADKSHRLRQMRGEELQGLDIEELQQLEKALETGLTRVIETKSDKIMSEISELQKKGMQLMDENKRLRQQGTQLTEENERLGMAES-ENAAVYEEGQSSESITNAGNSTG--APVDSESSDTSLRLGLPYGG--

AtAGL24 MAREKIRIKKIDNITARQVTFSKRRRGIFKKADELSVLCDADVALIIFSATGKLFEFSSSRMRDILGRYSLHASNINKLMDPPSTHLR-LENCNLSRLSKEVEDKTKQLRKLRGEDLDGLNLEELQRLEKLLESGLSRVSEKKGECVMSQIFSLEKRGSELVDENKRLRDKLETLER-----------AKLTTLKEALETESVTTNVSSYDS-GTPLEDDSDTSLKLGLPS--WE

;

END;
